# Comparative immune responses to *Mycobacterium tuberculosis* in people with latent infection or sterilizing protection

**DOI:** 10.1016/j.isci.2023.107425

**Published:** 2023-07-20

**Authors:** Emilie Jalbert, Cuining Liu, Vidya Mave, Nancy Lang, Anju Kagal, Chhaya Valvi, Mandar Paradkar, Nikhil Gupte, Rahul Lokhande, Renu Bharadwaj, Vandana Kulkarni, Amita Gupta, Adriana Weinberg

**Affiliations:** 1 Department of Pediatrics, University of Colorado-Denver Anschutz Medical Campus, Aurora, CO, USA; 2 Department of Biostatistics and Informatics, Colorado School of Public Health, University of Colorado-Denver Anschutz Medical Campus, Aurora, CO, USA; 3 Byramjee Jeejeebhoy Government Medical College- Johns Hopkins University Clinical Research Site (BJGMC-JHU CRS), Pune, Maharashtra, India; 4 Johns Hopkins Center for Infectious Diseases in India, Pune, Maharashtra, India; 5 School of Medicine, Center for Clinical Global Health Education (CCGHE), Johns Hopkins University, Baltimore, MD, USA; 6 Department of Microbiology, Byramjee Jeejeebhoy Government Medical College and Sassoon General Hospital, Pune, Maharashtra, India; 7 Department of Pediatrics, Byramjee Jeejeebhoy Government Medical College and Sassoon General Hospital, Pune, Maharashtra, India; 8 Department of Pulmonary Medicine, Byramjee Jeejeebhoy Government Medical College and Sassoon General Hospital, Pune, Maharashtra, India; 9 Departments of Pediatrics, Medicine and Pathology, University of Colorado-Denver Anschutz Medical Campus, Aurora, CO, USA

**Keywords:** Molecular biology, Immunology, Virology

## Abstract

There is great need for vaccines against tuberculosis (TB) more efficacious than the licensed BCG. Our goal was to identify new vaccine benchmarks by identifying immune responses that distinguish individuals able to eradicate the infection (TB-resisters) from individuals with latent infection (LTBI-participants). TB-resisters had higher frequencies of circulating CD8^+^ glucose monomycolate (GMM)+ Granzyme-B+ T cells than LTBI-participants and higher proportions of polyfunctional conventional and nonconventional T cells expressing Granzyme-B and/or PD-1 after *ex vivo M. tuberculosis* stimulation of blood mononuclear cells. LTBI-participants had higher expression of activation markers and cytokines, including IL10, and IFNγ. An exploratory analysis of BCG-recipients with minimal exposure to TB showed absence of CD8+GMM+Granzyme-B+ T cells, lower or equal proportions of Granzyme-B+PD-1+ polyfunctional T cells than TB-resisters and higher or equal than LTBI-participants. In conclusion, high Granzyme-B+PD-1+ T cell responses to *M. tuberculosis* and, possibly, of CD8+GMM+Granzyme-B+ T cells may be desirable for new TB vaccines.

## Highlights

• TB-resisters eradicate TB infection providing immunologic goals for new TB vaccines
• TB-resisters have higher CD8+GMM+GzB+ T cells than people with latent TB (LTB)
• TB-resistance is associated with higher GranzB+PD1+ polyfunctional T cells than LTB
• BCG generates less CD8+GMM+GranzB+ or polyfunctional T cells than TB-resistance

## Graphical abstract

**Figure:**
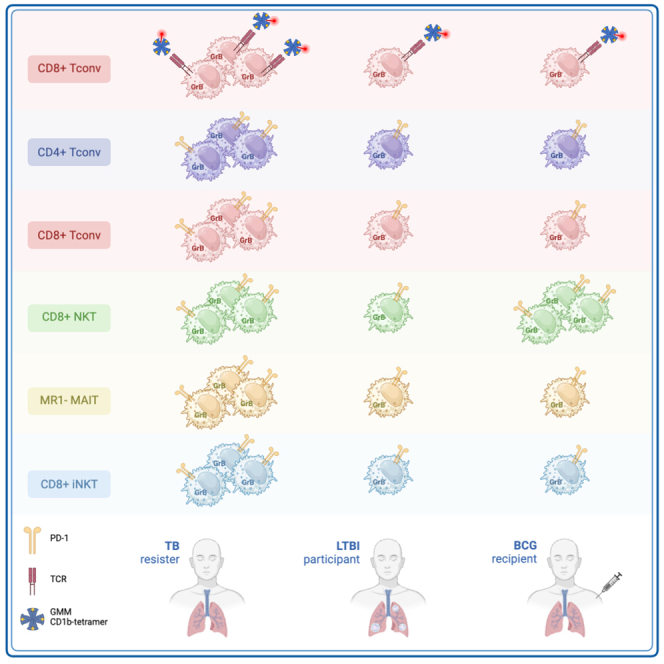

## Introduction

Tuberculosis (TB) is a major global health problem. There are an estimated 2 billion people infected with *M. tuberculosis* (*Mtb*) and 1.5 million annual deaths. Vaccines are the most powerful tools for limiting the morbidity and mortality of many infectious diseases, but Bacillus Calmette-Guérin (BCG)—the only licensed TB vaccine—confers limited protection.^1^^,^^2^^,^^3^^,^^4^^,^^5^^,^^6^^,^^7^ BCG administered to infants at birth is ∼80% effective in preventing disseminated and central nervous system TB. In contrast, BCG has variable efficacy against pulmonary TB when administered to infants or adults, and some studies could not demonstrate any efficacy.^1^^,^^2^^,^^3^^,^^4^^,^^5^^,^^6^^,^^7^^,^^8^ In addition, recent studies showed that BCG revaccination did not prevent acquisition of latent TB infection (LTBI) measured by IFNγ release assays (IGRA) conversion in adolescents despite boosting immune responses, albeit more BCG than placebo recipients reverted IGRA 3 to 6 months after conversion^9^^,^^10^^,^^11^ BCG generates TB-specific Th1 cell-mediated immunity (CMI) that has been considered necessary and sufficient for protection against TB, although this concept has been disputed by recent studies.^12^^,^^13^^,^^14^ Moreover, recent vaccine candidates that generate more robust Th1 CMI than BCG against the *Mtb* antigen 85A, did not have greater efficacy against pulmonary TB than BCG.^12^^,^^15^^,^^16^^,^^17^^,^^18^ Additional evidence that *Mtb* Th1 responses may not predict protection against *Mtb* infection is that 10 to 20% of the individuals with latent TB infection, as defined by the presence of *Mtb* CMI measured by tuberculin skin test (TST) or IGRA, a measure of Th1 immunity, develop active TB disease over time. In contrast, 20% of individuals with household contacts with highly contagious active pulmonary TB infection do not develop clinical disease and have negative TST or IGRA.^19^ These individuals, designated as TB-resisters, have antibodies and limited adaptive CMI responses to *Mtb*, confirming prior *Mtb* infection, but their T cells do not produce IFNγ in response to *Mtb ex vivo* restimulation.^20^^,^^21^ They also have genetic traits that differentiate them from people with LTBI.^22^^,^^23^^,^^24^^,^^25^ TB-resisters are deemed to have sterilizing immunity against *Mtb*.^26^^,^^27^ However, the mechanisms of protection against *Mtb* infection in TB-resisters are incompletely understood.^21^^,^^28^ Identifying these mechanisms would benefit the development of new vaccines that may cf. sterilizing immune protection against *Mtb*.^29^

In addition to adaptive CMI, innate immunity is a critical mechanism of protection against infections. Innate immunity is rapidly deployed and constitutes the first line of immune-mediated defense. Moreover, innate immune responses critically contribute to the development of antigen-specific adaptive CMI and establish a feedback mechanism that allows them to be also boosted by adaptive CMI.^30^^,^^31^^,^^32^^,^^33^^,^^34^^,^^35^^,^^36^ Persistent memory-like innate immune responses have been described against viruses, tumors, and other antigens, including *Mtb*.^32^^,^^36^^,^^37^^,^^38^^,^^39^^,^^40^^,^^41^^,^^42^^,^^43^^,^^44^^,^^45^^,^^46^^,^^47^ Natural killer (NK) cells, γδ T cells, NKT cells, invariant NKT (iNKT) cells, macrophages, monocytes (mono), and dendritic cells (DC) have been shown to undergo clonal expansions and/or epigenetic modifications after exposure to immunogens that can cf. antigen specificity and/or allow them to activate transcription programs that improve their functionality.^47^^,^^48^ Recent studies described a germline encoded, mycolyl-reactive iNKT cell subset (GEMT) that recognizes the lipid glucose monomycolate in the context of CD1b and participates in the elimination of *Mtb* from the host.^44^^,^^46^^,^^49^ Compared to other iNKT, CD1b-restricted GEMT cells have a more diverse T cell receptor repertoire, which permitted the identification of GEMT cell clonal expansions in patients recovering from TB and their persistence in the host for several years after *Mtb* elimination.^44^^,^^46^ Another nonconventional T cell subset of interest is the mucosal-associated invariant T cells (MAIT) that developmentally share some characteristics both with iNKT and γδ T cells. MAIT can recognize microbial riboflavin derivatives presented by the major histocompatibility-related receptor 1 (MR1) and play a critical role in antibacterial, including *Mtb*, pulmonary defenses via cytokine production and cytotoxicity.^50^^,^^51^^,^^52^ MR1+ MAIT were shown to account for most CD8^+^ T cell production of IFNγ in response to BCG.^53^ MR1- MAIT lack the receptor for antigen recognition and are deemed to respond to secreted cytokines.^54^^,^^55^ The role of innate immune responses in sterilizing immunity against *Mtb* infection has not been studied. However, *in vitro*, trained immunity controls mycobacterial outgrowth.^56^ It is important to note that vaccines, including BCG, adjuvanted vaccines, and vectored vaccines, have been recently shown to generate trained immunity.^57^^,^^58^^,^^59^^,^^60^ Thus, information on the association of trained immunity with sterile protection against *Mtb* infection may be used in the development of new and improved TB vaccines.

The overarching goal of our study was to identify innate and adaptive immune responses that differentiate TB-resisters from people with LTBI (LTBI-participants) among household contacts of active pulmonary TB cases that could be targeted by new TB vaccines. We leveraged a longitudinal cohort study of household contacts of individuals with active pulmonary TB disease [Cohort for TB research by The Indo-US Medical Partnership Multicentric Prospective Observational Study (C-TRIUMPH)] in Pune, India, by using advanced spectral flow cytometry technology and high-dimensional analytic tools on peripheral blood mononuclear cells (PBMC) archived from the study participants.^27^^,^^61^^,^^62^ To further understand how the responses to *Mtb* in LTBI-participants and TB-resisters may differ from those induced by BCG, we also included in our analysis a group with documented BCG vaccination and minimal exposure to TB.

## Results

### Characteristics of the study population

The study used PBMC from 13 TB-resisters and 11 LTBI-participants in C-TRIUMPH, who had household contact with adults recently diagnosed with active pulmonary TB (index TB case) and lived in an area of high TB endemicity. TB-resisters were defined by TST and IGRA negative results at entry and during the 2-year C-TRIUMPH follow up. LTBI-participants had IGRA and/or TST positive results at entry in C-TRIUMPH. There were no appreciable differences in the baseline demographic characteristics and TB exposure scores between TB-resisters and LTBI-participants (Table 1). TB exposure scores have been designed to predict development of active and/or latent TB infection in household contacts of TB index cases. In C-TRIUMPH, the exposure score was comprised of 11 items, including presence of cough, pulmonary TB, smear positivity of the index case, if the index case was the household contact’s primary caregiver or mother, sleep location of the household contact, and whether the household contact lived in the same house as the index TB case. High exposure was defined as a score ≥6 for adults and ≥5 for children. Although TB exposure scores were found to be associated with TB-resister versus LTBI status primarily in people 5–15 years of age,^63^ we collected the exposure scores of all the participants in this study as an additional measure of uniformity between groups. Also recruited were 14 BCG-recipients with limited exposure to TB by virtue of having spent most of their lives in the United States or other countries with low incidence of TB. In addition to these differences in upbringing and environmental exposures, we noted that BCG-recipients significantly differed from C-TRIUMPH participants in race/ethnicity, but not in age or BMI.

**Table 1:** Characteristics of the study population

	TB-Resisters	LTBI+ participants	BCG	p-Value^a^(TB-Resisters vs. LTBI+)	p-Value^a^(three-group)
N	13	11	14		
Median years of age (range)	24 (9, 65)	29 (7, 62)	29 (20, 41)	0.81	0.71
N Female sex (%)	6 (46)	4 (36)	10 (71.4)	0.70	0.19
N Race and Ethnicity (%)					
Asian (Indian subcontinent)	13 (100)	11 (100)	0 (0)	na	<0.0001
Asian (not Indian subcontinent)	0 (0)	0 (0)	3 (21.4)		
Black	0 (0)	0 (0)	2 (14.3)		
Hispanic Non-Black	0 (0)	0 (0)	5 (35.7)		
Non-Hispanic White	0 (0)	0 (0)	4 (28.6)		
Median BMI (range)	23.3 (13.1, 30.5)	20.4 (13.5, 29.8)	22.8 (20, 30.6)	0.49	0.49
Median exposure score (range)	7 (4, 9)	7 (4, 9)	na	0.84	na
N with BCG scar (%)	9 (64.3)	5 (35.7)	na	0.24	na

BCG, Bacille de Calmette-Guerin; BMI, Body mass index (kg/m^2^); LTBI, latent tuberculosis infection; N, number; na, not applicable.

a ANOVA for continuous variables and Fisher’s exact for categorical variables.

### Phenotypic and functional characteristics of innate and adaptive immune responses in TB-resisters and LTBI-participants

We used two high-dimensional flow cytometry panels [T cell and antigen-presenting cell (APC)] on PBMC stimulated with *Mtb* antigen or unstimulated controls (Table S1). In the T cell panel, we characterized CD4^+^ and CD8^+^ conventional T cells (Tconv), NKT, γδ T, GEMT, iNKT, MR1+ and MR1-MAIT, and NK cells. Functionality was assessed by expression of CD25 and CD69-activation markers; PD1 immunologic checkpoint receptor; CD107a (lysosome degranulation) and granzyme B (GranzB) cytotoxicity markers; GMCSF, IL2, IL10, IL17, IFNγ, and TNFα cytokines; and Ki67 proliferation marker. In the APC panel, we characterized functionality of CD14^+^ monocytes (Mono), CD123+ plasmacytoid DC (pDC), CD141+ cDC1, and CD1c+ cDC2 subsets using the following functional readouts: CD40, CD80, and CD83 activation markers; PDL1 immunologic checkpoint ligand; and GMCSF, IL1β, IL8, IL10, IL12p40, IL27, and TNFα cytokines. Gating trees are shown in Figures S1–S12.

**Figure 1:**
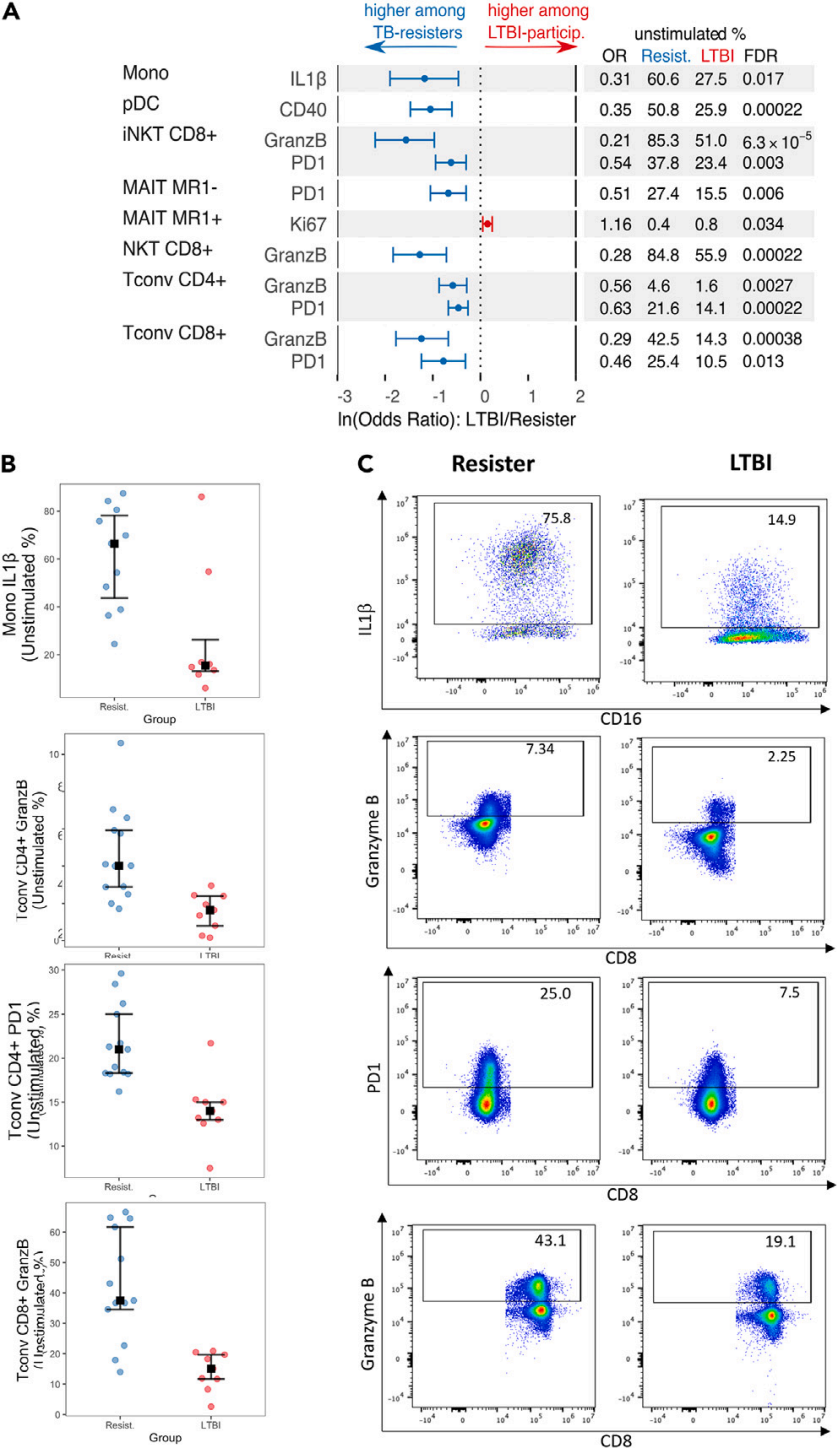
Functional phenotypes differentially expressed in unstimulated PBMC from TB-resisters and LTBI-participants Data were derived from 13 TB-resisters and 11 LTBI-participants. PBMC were analyzed after overnight rest in culture medium. (A) Forest plot displays ln-transformed odds ratios (lnOR) and 95% confidence intervals (CI). The dotted line shows no average effect (lnOR = 0, corresponding to OR = 1). Features on the right side of this line are more highly expressed among LTBI-participants (OR > 1), and features on the left side are more highly expressed among TB-resisters (OR < 1). The table shows the absolute OR and the means of each parameter in TB-resisters and LTBI-participants. (B) Scatterplots showing individual frequencies, medians, upper, and lower quartiles of IL1β+ Mono, CD4+GranzB + Tconv, CD4+PD1+ Tconv, and GranzB+CD8^+^ Tconv in TB-resisters (blue dots) and LTBI-participants (red dots). (C) Typical dot plots exemplifying the data shown in the scatterplots. Remaining scatter and dot plots of significant differences between TB-resisters and LTBI-participants are shown in Figure S2.

In *unstimulated* conditions, 10 functional cell subsets had significantly higher frequencies in TB-resisters compared with LTBI-participants, consisting of GranzB-expressing CD4^+^ Tconv, CD8^+^ Tconv, CD8^+^ iNKT and CD8^+^ NKT; PD1-expressing CD4^+^ Tconv, CD8^+^ Tconv, CD8^+^ iNKT and MR1- MAIT; CD40^+^ pDC; and IL1β+ Monos (Figures 1, S13, and Table S2). In contrast, we only detected a single subset (Ki67+MR1+ MAIT) with significantly higher frequencies in LTBI-participants compared with TB-resisters.

**Figure 2:**
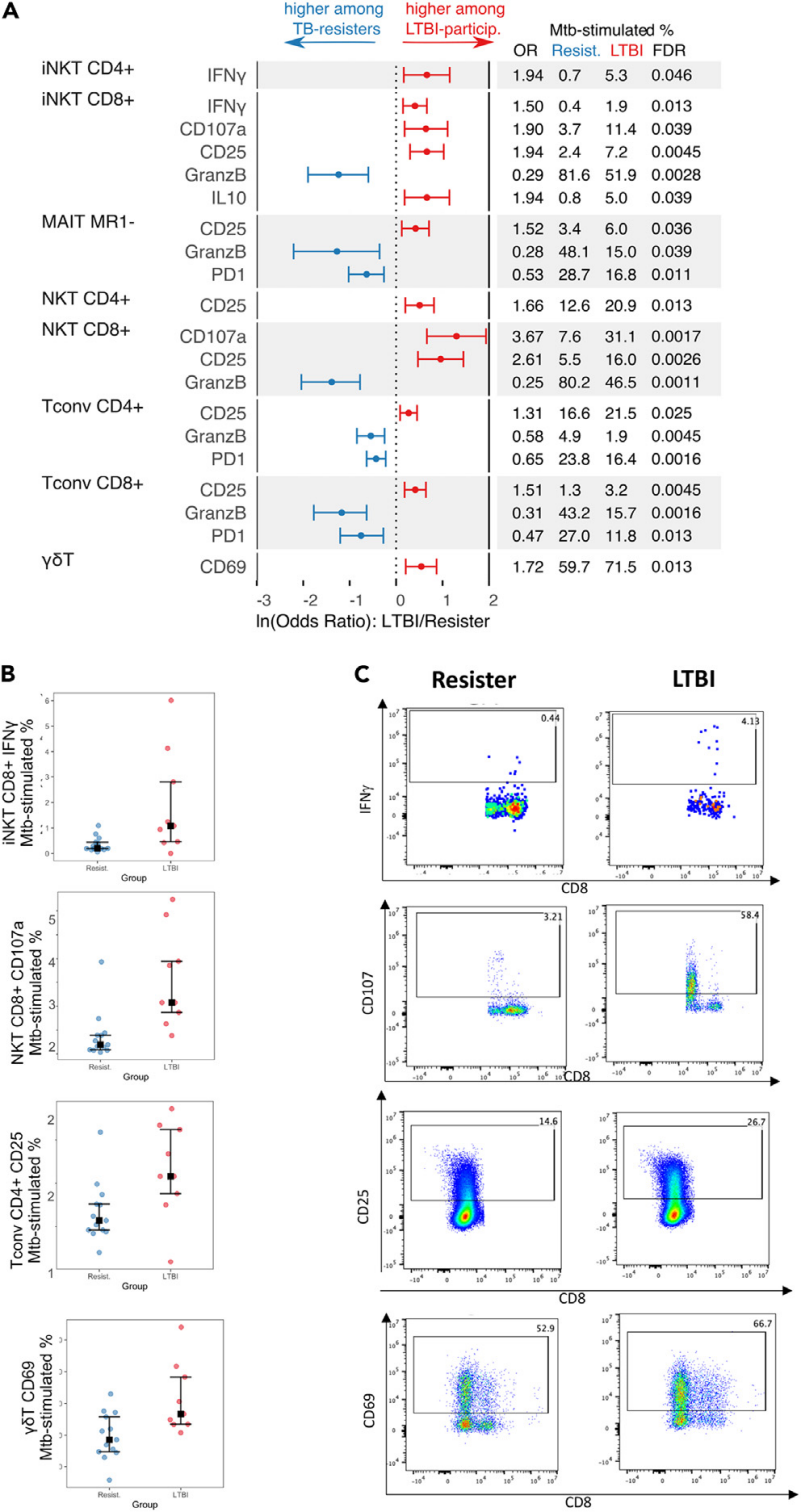
Functional phenotypes differentially expressed in *Mtb*-stimulated PBMC from TB-resisters and LTBI-participants Data were derived from 13 TB-resisters and 9 LTBI-participants that contributed PBMC to the T cell analysis. PBMC were analyzed after pre-optimized overnight stimulation with *Mtb* cell membrane (see STAR Methods). (A) Forest plot displays natural log (ln)-transformed odds ratios (lnOR) and 95% confidence intervals (CI). The dotted line shows no average effect (lnOR = 0, corresponding to OR = 1). Features on the right side of this line are more highly expressed among LTBI-participants (OR > 1), and features on the left side are more highly expressed among TB-resisters (OR < 1). The table shows the absolute OR and the means of each parameter in TB-resisters and LTBI-participants. (B) Scatterplots showing individual frequencies, medians, upper, and lower quartiles of CD8+IFNγ+ and CD8^+^CD107+ iNKT, CD4^+^CD25^+^ Tconv, and CD69^+^ γδ T cells in TB-resisters (blue dots) and LTBI-participants (red dots). (C) Typical dot plots exemplifying the data shown in the companion scatterplots. Remaining scatter and dot plots of significant differences between TB-resisters and LTBI-participants are shown in Figure S3.

Many of the functional differences between TB-resisters and LTBI-participants observed in unstimulated PBMC persisted after *Mtb ex vivo* stimulation (Figures 2, S14, and Table S3). Differences between TB-resisters and LTBI-participants in *Mtb-stimulated* PBMC included higher proportions of (1) GranzB-expressing CD4^+^ Tconv, CD8^+^ Tconv, CD8^+^ iNKT. CD8^+^ NKT and MR1- MAIT cells; and (2) PD1-expressing CD4^+^ Tconv, CD8^+^ Tconv and MR1- MAIT in TB-resisters; and higher proportions of (1) CD25-expressing CD4^+^ and CD8^+^ Tconv and NKT, CD8^+^ iNKT, and MAIT MR1-; (2) CD107+CD8^+^ iNKT and NKT; (3) IFNγ-expressing CD4^+^ and CD8^+^ iNKT; (4) CD8+IL10+ iNKT; and (5) CD69^+^ γδ T cells in LTBI-participants.

**Figure 3:**
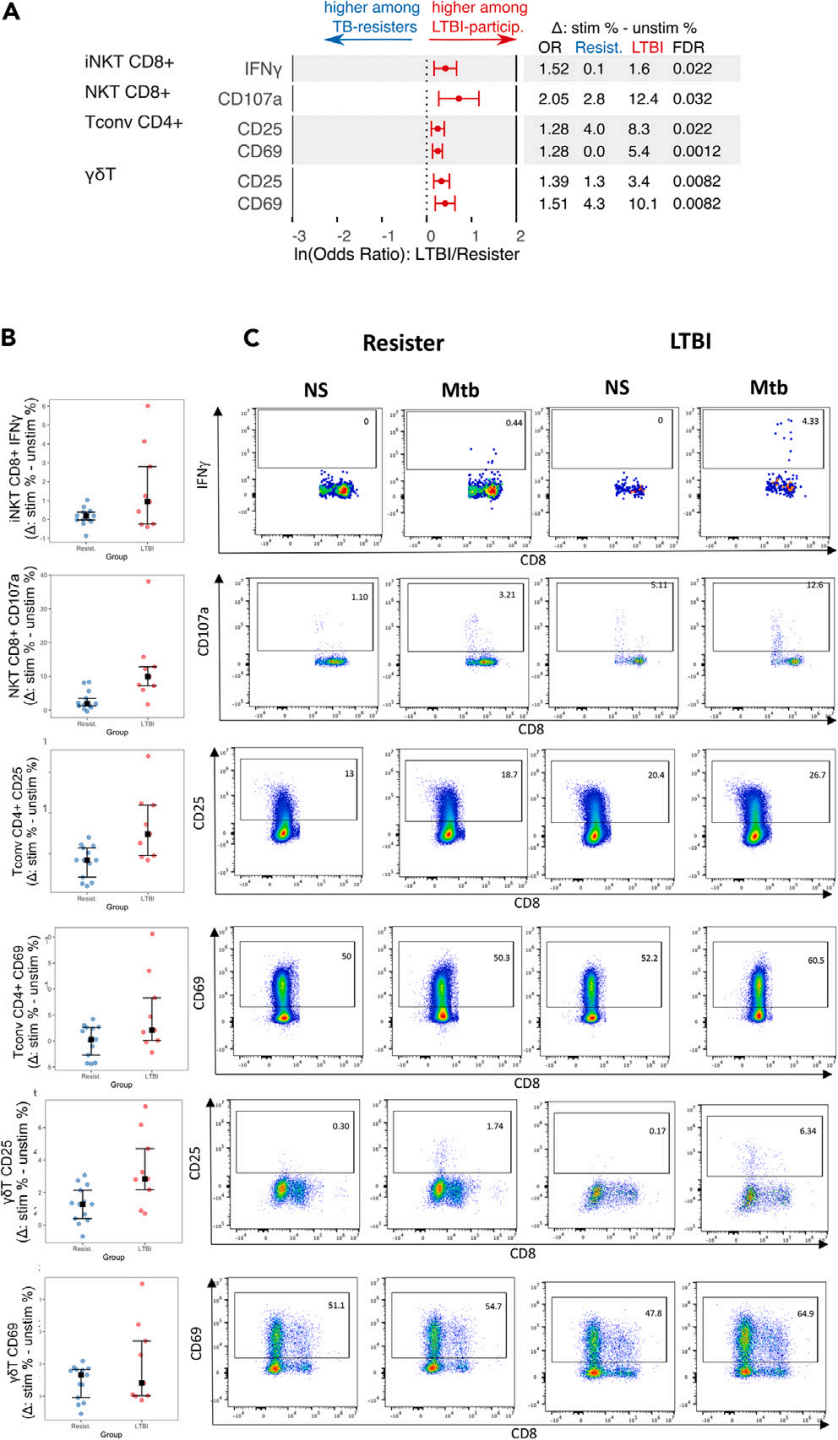
*Mtb*-specific memory responses in TB-resisters and LTBI-participants Data were derived from 13 TB-resisters and 9 LTBI-participants that contributed PBMC to the T cell analysis. *Mtb*-memory was assessed by subtracting the frequency of activated cell subsets in unstimulated PBMC from *Mtb*-stimulated conditions. (A) Forest plot displays ln-transformed odds ratios (lnOR) and 95% confidence intervals (CI) of the differences mentioned above. The dotted line shows no average effect (lnOR = 0, corresponding to OR = 1). Features on the right side of this line are more highly expressed among LTBI-participants (OR > 1), and features on the left side are more highly expressed among TB-resisters (OR < 1). The table shows the absolute OR and the means of each parameter in TB-resisters and LTBI-participants. (B) Scatterplots showing individual frequencies, medians, upper, and lower quartiles of the parameters with significant differences between TB-resisters (blue dots) and LTBI-participants (red dots). (C) Dot plots exemplifying the data shown int the companion scatterplots. Abbreviations: NS = unstimulated conditions; Mtb = Mtb-stimulated conditions.

*Mtb-memory* responses were characterized by subtracting frequencies of *unstimulated* from *Mtb*-*stimulated* corresponding PBMC*.* Using this definition, we identified 6 differentially expressed functional subsets with significantly higher frequencies in LTBI-participants compared with TB-resisters: CD25^+^ and CD69^+^ γδ T cells; CD4^+^CD25^+^ Tconv; CD8+IFNγ+ iNKT; and CD8^+^CD107a+ NKT (Figure 3, and Table S4). There were no *Mtb-memory* subsets with higher frequency in TB-resisters than in LTBI-participants. Of note, cytokine-producing conventional T cell subsets, including CD4^+^ T cells expressing GMCSF, IFNγ, IL2, IL10, IL17, and TNFα and CD8^+^ T cells expressing IFNγ, IL2, IL10, and TNFα were excluded from the analyses based on the pre-specified criteria of a difference <0.1% in ≥33% of participants in each group (see statistical methods).

### Polyfunctionality of the immune cell subsets in TB-resisters and LTBI-participants

**Figure 4:**
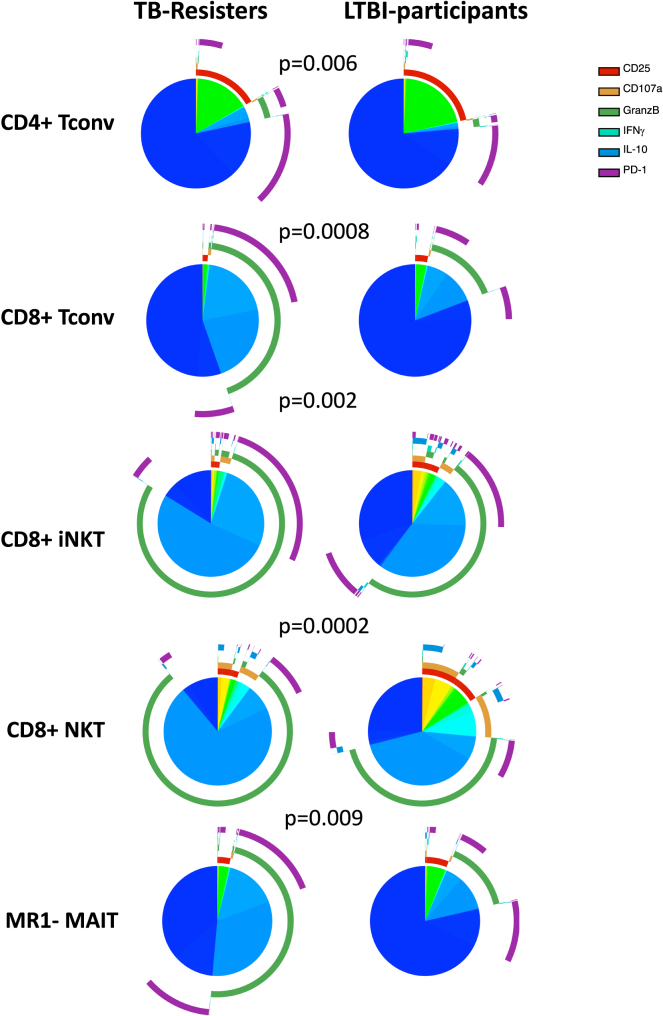
Polyfunctionality of T cell responses in TB-resisters and LTBI-participants Data were derived from *Mtb-stimulated* PBMC from 13 TB-resisters and 9 LTBI-participants. Polyfunctionality was analyzed using Boolean gating of the six most abundantly expressed markers consisting of CD25, CD107a, GranzB, IFNγ, IL10 and PD1. Pie charts show the proportions of cells expressing no markers in dark blue or ≥1 markers in colors that vary with the combination of markers expressed by each cell. Arches show the markers expressed by groups of responding cells. p values of the differences between the two groups were calculated by analysis of permutations using Spice software.

Tconv polyfunctionality has been previously shown to predict protection against TB and other infections.^64^^,^^65^^,^^66^^,^^67^^,^^68^ Here, we expanded the investigation of polyfunctionality to nonconventional T cells and NK subsets. Polyfunctionality was analyzed using Boolean gating of the six markers most abundantly expressed by NK and T cells (CD25, CD107a, GranzB, IFNγ, IL10, and PD1) in *Mtb-stimulated* conditions. Higher marker co-expression was observed in TB-resisters than in LTBI-participants on CD4^+^ and CD8^+^ Tconv and also on CD8^+^ iNKT, MR1- MAIT, and CD8^+^ NKT (Figure 4). Markers most frequently involved in dual expression were Granz B and PD1. There were no differences in polyfunctionality of NK and other T cell subsets in *Mtb*-*stimulated* conditions between TB-resisters and LTBI-participants (not depicted).

### Coordination of the immune responses in TB-resisters and LTBI-participants

**Figure 5:**
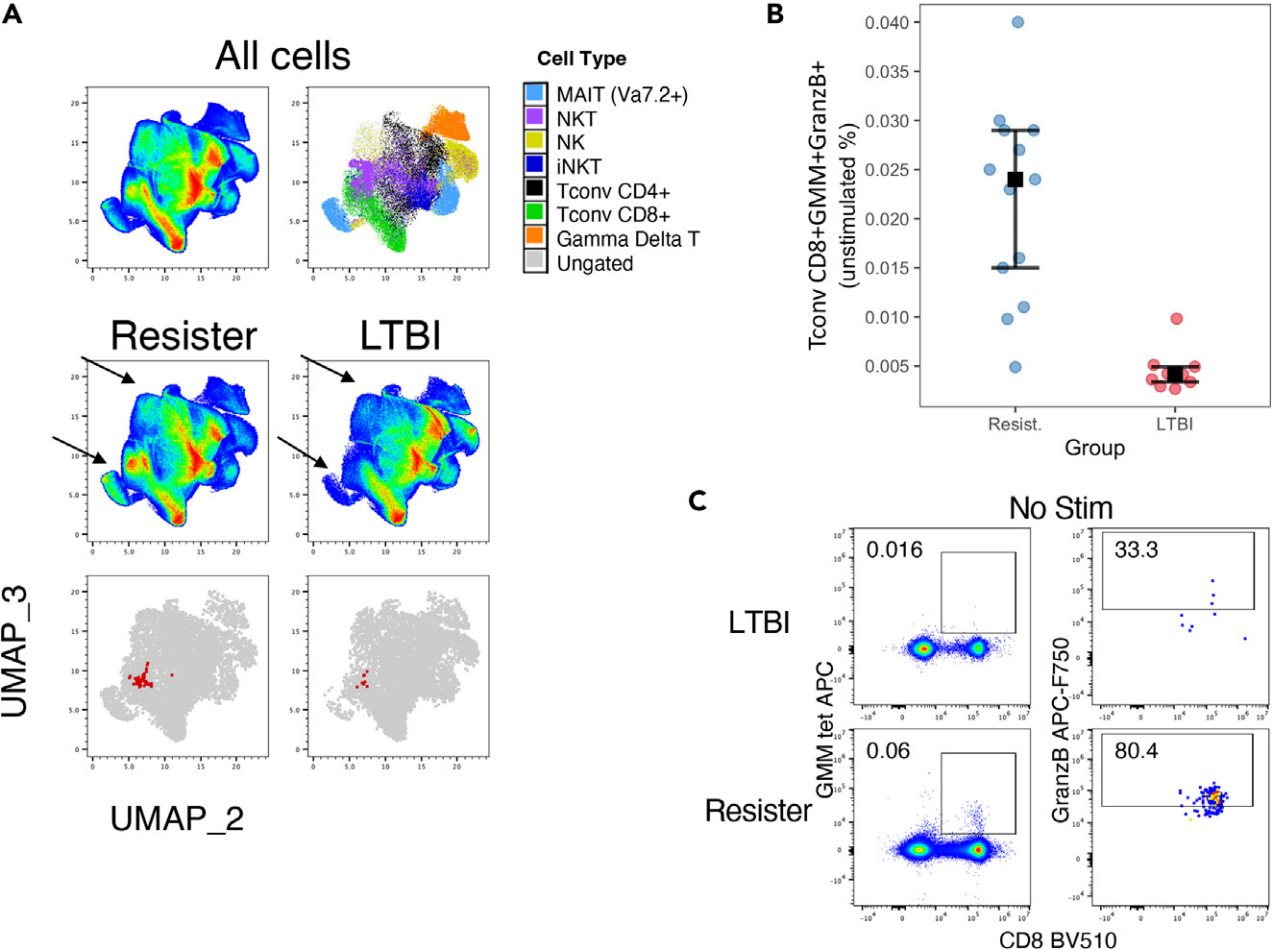
Comparison of CD8+GMM+GranzB+ Tconv subset in TB-resisters and LTBI-participants Data were derived from 13 TB-resisters and 9 LTBI-participants that contributed 2,948,000 events down sampled to 67,000 events in the cleanup gate. (A) T cell UMAPs showing concatenated stimulated and unstimulated cells (upper left corner), distribution of major cell subsets (upper right corner); Distribution of unstimulated T cells in resisters and LTBI-participants (middle row); CD8+GMM+GranzB+ cluster in TB-resisters and LTBI-participants (bottom row). (B) Individual results from each participant with medians, upper, and lower quartiles are identified on the graph. (C) Typical representation of the frequency of CD8+GMM+GranzB+ T cells in LTBI-participants and TB-resisters.

We complemented the analysis of manually gated PBMC subsets with Phenograph unbiased analysis, which identified 8 T cell and 1 APC clusters that significantly differed between TB-resisters and LTBI-participants in *unstimulated* PBMC; 3 T cell, and 1 APC clusters in *Mtb-stimulated* conditions; and 5 T cell and 3 APC *Mtb-memory* clusters (Figure S15). Most clusters confirmed findings already observed in the manually gated analysis, with the following exceptions: (1) Two T cell and one APC *Mtb*-*memory* clusters (Figure S15) with higher relative frequencies in TB-resisters than LTBI-participants, in contrast to the manual gating analysis that did not identify excess *Mtb-memory* subsets in TB-resisters compared with LTBI-participants; and (2) A CD8+glucose monomycolate (GMM)+GranzB+ Tconv subset that had not been included in the prespecified manual gating analysis (Figures 5, and S15). This subset had higher frequency in *unstimulated* PBMC from TB-resisters compared with LTBI-participants (FDR-corrected p = 0.008).

### Immune responses in BCG-recipients

The analysis of functional phenotypes in BCG-recipients showed significant differences with TB-resisters in 54 *unstimulated,* 41 *Mtb-stimulated*, and 41 *Mtb-memory* PBMC subset responses (Figures S16–S18, andTables S5–S7). Compared to LTBI-participants, BCG-recipients showed differences in 56 *unstimulated*, 32 *Mtb-stimulated*, and 24 *Mtb-memory* responses (Figures S19–S21 and Tables S8–S10).

**Figure 6:**
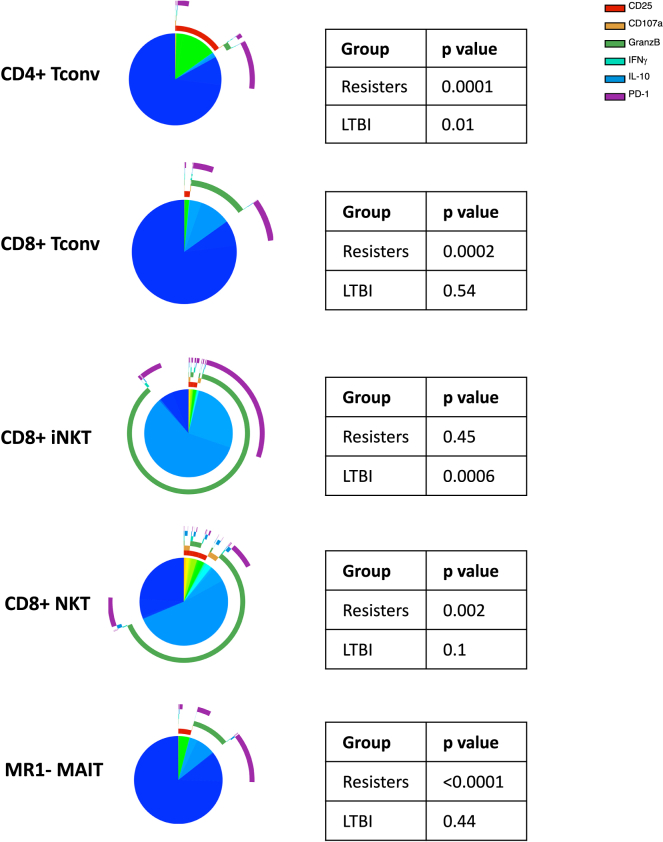
Polyfunctionality of T cell responses in BCG-recipients Data were derived from *Mtb-stimulated* PBMC of 14 BCG-recipients. Polyfunctionality was analyzed using Boolean gating of the six most abundantly expressed markers consisting of CD25, CD107a, GranzB, IFNγ, IL10 and PD1. Pie charts show the proportions of cells expressing no markers in dark blue or ≥1 markers in colors that vary with the combination of markers expressed by each group of cells. Arches show the markers expressed by each group of responding cells. Tables show unadjusted p values for differences with TB-resisters or LTBI-participants calculated by analysis of permutations using Spice software.

To focus the comparisons of BCG-recipients with TB-resisters and LTBI-participants, we analyzed the polyfunctionality of *Mtb-stimulated* T cell subsets with significantly different polyfunctionality in TB-resisters compared with LTBI-participants, including CD4^+^ and CD8^+^ Tconv, CD8^+^ iNKT, CD8^+^ NKT, and MR1- MAIT cells (Figure 6). The results showed significantly higher polyfunctionality in TB-resisters compared with BCG-recipients for all subsets except for CD8^+^ iNKT, which did not differ between the two groups. Notably, polyfunctionality of CD8^+^ iNKT cells was significantly higher in BCG-recipients than in LTBI-participants. Conversely, polyfunctionality of CD4^+^ Tconv was significantly higher in LTBI-participants than in BCG-recipients. CD8^+^ Tconv, CD8^+^ NKT, and MR1- MAIT subsets showed similar polyfunctionality in BCG-recipients and LTBI-participants. These data indicate that BCG and, possibly, other vaccines may generate higher polyfunctional responses than LTBI and reach levels observed in TB-resisters.

One of the two recent descriptions of GMM+ Tconv showed their presence in response to BCG administration in infants, but not in adults.^69^ On comparing the frequency of CD8+GMM+GranzB+ T cells in BCG-recipients with TB-resisters and LTBI-participants in our study, we also found very small frequencies of this cell subset (≤0.005% out of total lymphocytes) in BCG-recipients, significantly lower compared with either of the *Mtb*-exposed groups (FDR-corrected p ≤ 0.00013; Figure S22).

## Discussion

The goal of this study was to characterize responses unique to TB-resisters compared to LTBI-participants that may be targeted by new TB vaccines. A consistent finding was increased GranzB-expressing conventional and nonconventional T cells in *Mtb-stimulated* and *unstimulated* PBMC of TB-resisters. This hallmark was observed in the prespecified manual gating and in the unsupervized cluster analyses and included expression of GranzB by Tconv, NKT, and iNKT cells. The analysis of *Mtb-memory* responses revealed higher cytotoxic responses in TB-resisters using the unbiased cluster analysis but not the manual gating. Collectively, these findings suggest that TB-resisters may mount higher and/or faster cytotoxic responses upon exposure to *Mtb* than LTBI-participants, which may contribute to the clearance of the infectious agent before establishing latency.

Of special interest was a population of CD8+GMM+GranzB+ Tconv that was not a part of the prespecified manual gating but was identified by the Phenograph cluster analysis. This cell subset, which has only recently been described, was found in people with active TB, in whom CD8+GMM+ T cells displayed upregulated cytotoxic transcription profiles.^70^ The authors concluded that this cell subset may contribute to the clearance of *Mtb*-infected host cells. Notably, we found higher proportions of CD8+GMM+GranzB+ T cells in TB-resisters compared with LTBI-participants, suggesting that this cell subset may contribute to the sterilizing immune protection of TB-resisters against *Mtb* infection.

TB-resisters also had higher proportions of PD1-expressing Tconv, iNKT, and MR1- MAIT than LTBI-participants after *ex vivo Mtb stimulation*. PD1 is an immunologic checkpoint inhibitor commonly expressed on activated T cells during acute or chronic infection.^71^ PD1 expression contributes to quenching the immune response in tumors and may play a similar role in infections after removal of the stimulating agent, limiting the inflammatory response and tissue destruction. Recent reports showed an association of increased T cell activation with TB morbidity and with increased risk of active *Mtb* infection in BCG-recipients.^72^^,^^73^^,^^74^ Moreover, PD1 deficient mice as well as people treated with PD1/PDL1-blocking agents have increased risk of *Mtb* infection, morbidity, and reactivation.^75^^,^^76^^,^^77^ Collectively, these observations suggest that the increased expression of PD1 in TB-resisters may limit their susceptibility to *Mtb* tissue destruction and, perhaps, propagation of infection.

A distinguishing characteristic of the immune response in TB-resisters from LTBI-participants was polyfunctionality. TB-resisters had higher proportions of polyfunctional Tconv and nonconventional T cells in *Mtb-stimulated* conditions. Polyfunctional CD4^+^ Tconv, characterized by expression of IL2, IFNγ, and/or TNFα have been previously associated with protection against active TB infection in animal models.^64^^,^^65^ Although the role of polyfunctional CD4^+^ T cell responses in protection against *Mtb* infection in humans remains uncertain after a recent study showed that IL2, IFNγ, and/or TNFα polyfunctional CD4^+^ T cell responses generated by BCG did not correlate with protection against TB disease in vaccinated infants,^14^ there is evidence from other human infections supporting the protective role of polyfunctional T cells.^66^^,^^67^^,^^68^^,^^78^^,^^79^ In our study, cytokine production made a minor contribution to the polyfunctional T cell responses, which predominantly expressed GranzB and PD1, and involved both conventional and nonconventional T cells. Polyfunctionality of nonconventional T cells has not been previously studied. Here, we showed increased polyfunctionality of NKT, iNKT, and MR1- MAIT cells in TB-resisters compared to LTBI-participants, which may contribute to enhanced protection against TB infection in TB-resisters through cytotoxicity.

*Mtb*-*memory* T cell responses identified by the expression of single activation markers were higher in LTBI-participants than in TB-resisters, although two *Mtb-memory* CD4^+^ Tconv clusters revealed by the Phenograph analysis had higher frequencies in TB-resisters than in LTBI-participants. These data confirm that TB-resisters develop *Mtb*-specific T cell memory, but in general *Mtb-memory* responses are higher in LTBI-participants. This finding may be related to the observation that 25% of people with LTBI have evidence of active *Mtb* replication, which continuously exposes the immune system to *Mtb* antigens and maintains high levels of *Mtb*-specific T cells.^80^^,^^81^^,^^82^^,^^83^

APC displayed few differentiating features between TB-resisters and LTBI-participants. However, the Phenograph analysis identified an activated Mono cluster in response to *Mtb stimulation* with higher frequency in TB-resisters than LTBI-participants. Additional studies are needed to further characterize the potential role of APC-trained immunity in protection against TB.

The analysis of *Mtb-memory* responses in BCG-recipients using single markers of activation showed both higher and lower responses in multiple T cell and APC subsets compared with TB-resisters or LTBI-participants. These differences may have been related to the difference in environmental exposures, including *Mtb*, in addition to genetic backgrounds of BCG-recipients compared with TB-resisters or LTBI-participants. The Boolean analysis of polyfunctional responses helped focus the differences between BCG-recipients and LTBI-participants or TB-resisters. Overall, BCG-recipients had lower frequencies of *Mtb-stimulated* polyfunctional T cells than TB-resisters, but similar to LTBI-participants. Notably, CD8^+^ iNKT cells had higher polyfunctionality in BCG-recipients compared to LTBI-participants clearly demonstrating the ability of the vaccine to generate polyfunctional responses higher than the infection. The corollary of this observation is that new TB vaccines may generate immune responses that protect against TB disease better than the immune responses of people with LTBI.

We conclude that both innate and adaptive responses distinguish TB-resisters from LTBI-participants. Unique elements of the cell-mediated immune responses to *Mtb* in TB-resisters are high-cytotoxic potential, expression of immunologic checkpoint inhibitors, and polyfunctionality of Tconv and nonconventional T cells, suggesting that some or all may be key factors in an effective immune response against *Mtb*. Although TB-resisters displayed these characteristics even without *ex vivo Mtb stimulation* in many cases, it is reasonable to propose that a vaccine that increases some or all these responses in the form of *Mtb-memory* may be highly effective in protection against TB.

### Limitations of the study

Our study has several limitations, including the relatively small number of participants, which may have restricted our ability to detect differences with small effect sizes and increased the risk of outliers to bias the results. Cytokine-producing conventional T cell subsets, including CD4^+^ T cells expressing GMCSF, IFNγ, IL2, IL10, IL17, and TNFα and CD8^+^ T cells expressing IFNγ, IL2, IL10, and TNFα were excluded from the analyses of *Mtb* memory responses because the low variability of the results and the tendency to recapitulate results in unstimulated conditions. This may have decreased our ability to identify *Mtb* memory responses in TB-resisters. In addition, BCG-recipients had different demographic characteristics compared with C-TRIUMPH participants (e.g., race/ethnicity). Although our study did not measure antibody responses, which have been recently shown to differentiate TB-resisters from LTBI-participants,^21^^,^^84^ the CMI analysis was comprehensive and revealed findings unique in TB-resisters.

## STAR★Methods

### Key resources table

**Table:** 

REAGENT or RESOURCE	SOURCE	IDENTIFIER
**Antibodies**		

IL-8 BV421	BD Biosciences	Cat# 563310, RRID:AB_2738131
CD16-eFluor450	Thermo Fisher Scientific	Cat# 48-0168-41, RRID:AB_1272122
CD40 BV480	BD Biosciences	Cat# 746330, RRID:AB_2743653
CD80 BV510	Biolegend	Cat# 305233, RRID:AB_2687023
CD1c BV605	Biolegend	Cat# 331537, RRID:AB_2629761
CD123 BV650	Biolegend	Cat# 306019, RRID:AB_11218792
IL-10 BV711	BD Biosciences	Cat# 564050, RRID:AB_2738564
CD141 BV750	BD Biosciences	Cat# 747244, RRID:AB_2871963
CD274 (PD-L1) BV785	Biolegend	Cat# 329735, RRID:AB_2629581
CD11c BB515	BD Biosciences	Cat#564491
HLA-DR Alexa Fluor 488	Biolegend	Cat# 307619, RRID:AB_493176
CD3 PerCP-Cy5.5	Biolegend	Cat# 300327, RRID:AB_1575010
CD56 PerCP-Cy5.5	Biolegend	Cat# 362505, RRID:AB_2563914
CD19 PerCP-Cy5.5	Biolegend	Cat# 302229, RRID:AB_2275547
CD20 PerCP-Cy5.5	Biolegend	Cat# 302325, RRID:AB_893285
CD83 PerCP-eFluor710	Thermo Fisher Scientific	Cat# 46-0839-42, RRID:AB_10609339
IL-1β PE	BD Biosciences	Cat# 340516, RRID:AB_400439
GM-CSF PE-Dazzle 594	Biolegend	Cat# 502317, RRID:AB_2616930
EBI3 (IL-27 subunit) APC	Thermo Fisher Scientific	Cat# 17-7358-42, RRID:AB_2573261
IL12/IL-23 p40 Alexa Fluor 647	Biolegend	Cat# 501818, RRID:AB_2124518
TNFα Alexa Fluor 700	Thermo Fisher Scientific	Cat# 56-7349-41, RRID:AB_10670754
CD14 APC-Fire750	Biolegend	Cat# 301853, RRID:AB_2632659
Zombie NIR Fixable Viability Dye	Biolegend	Cat#423105
MR1 BV421, 5-OP-RU	NIH Tetramer Core Facility	
CD56 BV480	BD Biosciences	Cat# 566162, RRID:AB_2739559
CD8 BV510	Biolegend	Cat# 344731, RRID:AB_2564623
IL-17 BV570	Biolegend	Cat# 512323, RRID:AB_11150589
TCR Vα7.2 BV605	Biolegend	Cat# 351719, RRID:AB_2562595
IL-2 BV650	Biolegend	Cat# 500333, RRID:AB_11147166
CD279 (PD-1) BV711	Biolegend	Cat# 329927, RRID:AB_11218612
IFNγ BV750	BD Biosciences	Cat# 566357, RRID:AB_2739707
CD107a (LAMP-1) BV785	Biolegend	Cat# 328643, RRID:AB_2565967
CD25 BB515	BD Biosciences	Cat#564468
Unloaded CD1b tetramer FITC	In-house	
CD3 Alexa Fluor 532	Thermo Fisher Scientific	Cat# 58-0038-41, RRID:AB_11219069
TNFa PerCP-Cy5.5	Thermo Fisher Scientific	Cat# 45-7349-41, RRID:AB_10733023
Ki67 PerCP-eFluor710	Thermo Fisher Scientific	Cat# 46-5699-41, RRID:AB_10804404
TCR γδ PE	BD Biosciences	Cat# 347907, RRID:AB_400359
IL-10 PE-Dazzle 594	Biolegend	Cat# 506811, RRID:AB_2632782
CD69 PE-Cy5	Biolegend	Cat# 310907, RRID:AB_314842
TCR Vα24-Jα18 PE-Cy7	Biolegend	Cat# 342911, RRID:AB_2562229
GMM-CD1b tetramer APC	In-house	
GM-CSF Alexa Fluor 647	BD Biosciences	Cat# 562257, RRID:AB_11152076
CD161 Alexa Fluor 700	Biolegend	Cat# 339941, RRID:AB_2565869
Granzyme B APC-Fire750	Biolegend	Cat# 372209, RRID:AB_2728376
Human TruStain FcX	Biolegend	Cat# 422302, RRID:AB_2818986
Brilliant Stain Buffer Plus	BD Biosciences	Cat#566385
True-Stain Monocyte blocker	Biolegend	Cat#426103
FoxP3 Staining Buffer Set	Thermo Fisher Scientific	Cat#00-5523-00
Biological samples	C-TRIUMPH	https://www.ncbi.nlm.nih.gov/pmc/articles/PMC4769396/
TB Cell Membrane Fraction	Bei resources	Cat#NR-14832

**Software and algorithms**		

Algorithm	https://github.com/chooliu/Sterile_vs_Latent_TB_Immunity	
Software	R v4.0.2	

**Other**		

Cytek Aurora	Cytek	

### Resource availability

#### Lead contact

Further information and requests for resources and reagents should be directed and will be fulfilled by the lead contact, Adriana Weinberg (Adriana.Weinberg@cuanschutz.edu).

#### Materials availability

This study did not generate new unique reagents.

### Experimental model and study participant details

#### Participants

Cryopreserved PBMC were obtained from 13 TB-resisters and 11 LTBI-participants in C-TRIUMPH.^27^^,^^61^^,^^62^ Both LTBI-participants and TB-resisters were household contacts of adults recently diagnosed with smear- or PCR-positive active pulmonary TB living in an area of high TB endemicity (incidence of TB >200 cases/100,000 population/year). Exclusion criteria from this substudy were diabetes, immune compromising conditions and age >65 years. C-TRIUMPH collected extensive health and social information on each household contact, including exposure scores and receipt of BCG. LTBI-participants were defined by positive IGRA and/or TST at entry. TB-resisters had negative IGRA and TST at entry and over the 2 years of follow up. BCG-recipients whose immunization status was documented by medical history, who lived most of their lives outside of TB endemic areas, and who had negative IGRA were enrolled at the University of Colorado Anschutz Medical Campus (CU-AMC).

#### Declaration

The study was approved by the Colorado Multiple Institutional Review Board (COMIRB) and by the IRBs of the Byramjee JeejeebhoyGovt. Medical College, India and Johns Hopkins Medicine. All participants signed informed consent.

### Method details

#### IGRA testing used quantiferon gold in tube kit (qiagen) performed as per manufacturer’s instructions

##### Flow cytometry

Testing conditions were optimized using a convenience sample of PBMC from LTBI-participants and donors without previous exposure to TB or BCG submitted to stimulation for several time intervals with different concentrations of irradiated *Mtb*, whole cell lysate, heat- killed bacteria, cell membrane, PPD and BCG. *Mtb* cell membrane, PPD, and BCG generated similar IFNγ and IL2 responses in a dual-color FLUOROSPOT using a convenience sample of PBMC from 10 people diagnosed with LTBI by IGRA. Using PBMC from the same people with LTBI and donors without exposure to TB or BCG, *Mtb* cell membrane provided better discrimination of innate immune responses between LTBI-participants and TB-unexposed individuals compared with whole cell lysate, heat-killed, or irradiated *Mtb*. Based on these optimization results, we used *Mtb* cell membrane for this study. PBMC were thawed in serum-free media (AIM V, Gibco) supplemented with benzonase (50 units/mL, Millipore). PBMC were resuspended at 2∗10^6^ cells/ml and stimulated with cell membrane (BEI Resources Strain CDC1551, Cell Membrane Fraction, NR-14832) at 20ug/ml in AIM V or unstimulated overnight. Brefeldin-A and Monensin (SIGMA) at 5ug/ml each were added for the last 4h of culture. For the T-cell panel, CD107a-BV785 (Biolegend) was added for the last 4h of culture. Cells were washed with PBS, stained with Zombie NIR Fixable Viability dye (Biolegend), and washed with PBS+1% albumin. For the APC panel, cells were treated with Human TruStain FcX (Biolegend), surface-stained with CD16-eFluor450, CD83-PerCP-eFluor710 (Thermo Fisher/eBioscience), CD40-BV480, CD141-BV750, CD11c-BB515 (BD Biosciences), CD80-BV510, CD1c-BV605, CD123-BV650, PDL1-BV785, HLA-DR-AlexaFluor488, CD3-, CD19-, CD20- and CD56-PerCP-Cy5.5, CD14-APC-Fire750 (Biolegend), brilliant stain buffer plus (BD Biosciences) and True-stain monocyte blocker (Biolegend). Cells were washed and treated with eBioscience Foxp3 Staining Buffer Set (Thermo Fisher/eBioscience), then stained with IL8-BV421, IL10-BV711, IL1β-PE (BD Biosciences), GMCSF-PE-Dazzle594, IL12p40-AlexaFluor647 (Biolegend), IL27-APC, TNFα-AlexaFluor700 (Thermo Fisher/eBioscience) and brilliant stain buffer plus. For the T-cell panel, cells were treated with 50% human AB serum (Gemini) in PBS+1% albumin, washed and surface-stained with tetramers: CD1b-unloaded-FITC, CD1b-GMM-APC, and MR1-BV421. Cells were washed, stained with TCR Vα7.2-BV605, TCR Vα24-Jα18-PE-Cy7 (Biolegend) and TCR γδ-PE (BD Biosciences) followed by CD16-eFluor450, CD3-AlexaFluor532 (Thermo Fisher/eBioscience), CD56-BV480, CD25-BB515 (BD Biosciences), CD8-BV510, PD1-BV711, CD69-PE-Cy5, CD161-AlexaFluor700 (Biolegend) and brilliant stain buffer plus. Cells were washed, treated with Foxp3 Staining Buffer Set, then stained with IL17-BV570, IL2-BV650, IL10-PE-Dazzle594, GranzymeB-APC-Fire750 (Biolegend), IFNγ-BV750, GMCSF-AlexaFluor647 (BD Biosciences), Ki67-PerCP-eFluor710, TNFα-PerCP-Cy5.5 (Thermo Fisher/eBioscience) and brilliant stain buffer plus. Cells from both panels were washed and fixed with PBS+1% paraformaldehyde, acquired on the Cytek Aurora™ (Cytek Biosciences), and analyzed with FlowJo (Becton Dickinson). Two leucopack controls were used in each run to ensure inter-run reproducibility and to examine technical effects.

##### Boolean analysis

Boolean combinatorial gates were created using the 6 cytokines/activation markers, generating 64 distinct activation phenotypes. Graphical representation was performed using SPICE 6.0 software.^85^ The data were analyzed using permutations (10,000 iterations) included in the software.

##### UMAP analysis

For T cell clustering, we used 67,000 down-sampled events from 13 TB-resisters and 9 LTBI-participants for a total of 2,948,000 events in the concatenated file. For APC clustering, we used data from 11 TB-resisters and 8 LTBI-participants for a total of 502,479 events in the concatenated file. Parameters were rescaled to ArcSinh prior to running UMAP with phenograph in FlowJo on the concatenated file using parameters, Euclidean distance, 15 nearest neighbors and minimum distance of 0.5.^86^

### Quantification and statistical analysis

Statistical analyses were performed on each cell subset in stimulated and unstimulated conditions. The number of samples per comparison is indicated in the figure legends. No power analysis was performed.

To compare the average cell lineage frequencies obtained from manual and data-driven gating between groups, we used beta regression (*betareg* R package, v3.1-3) with a logit-link to model cell lineage percent (regression outcome; between 0 and 100% of events) on group (primary explanatory variable of interest; LTBI-participant versus TB-resisters), adjusting for participant age in years as a covariate.^87^ Similarly, to test for functional differences among *unstimulated* conditions, we modeled percent positivity (outcome; between 0 and 100% of cells in given lineage expressing functional marker) on group, correcting for age. “Rare” outcomes (i.e., outcomes of 0% in over 1/3^rd^ of participants) were excluded from testing due to their low variability among our participants. For each set of statistical tests, we defined significant between-group differences as a non-zero group regression coefficient (Wald test, multiple testing adjustment using the Benjamini-Hochberg false discovery rate < 0.05).^88^ We confirmed the statistical approach following exploratory data analyses (e.g., raw marker frequencies by group) and regression goodness-of-fit metrics (e.g., residual plots). We report effect sizes in terms of odds ratios (OR, ± 95% confidence intervals) and the median ± interquartile range for percent positivity.

To examine differences in functional markers under *Mtb-stimulated* conditions, we repeated this modeling procedure but with the stimulated percent positivity values as the regression outcome variable, adjusting for age. We also examined differences in *Mtb-memory* by additionally adjusting for each participant’s corresponding baseline values (unstimulated percent positivity values in their paired control sample) as a covariate. The *Mtb-memory* analysis thus emphasizes markers that were not already differentially abundant between groups prior to stimulation, or stimulation effects potentially masked by unstimulated differences. Here, adjusting for baseline levels is conceptually similar to “subtracting” the unstimulated values from the Mtb-stimulated values; as a consequence, we visualize *Mtb-memory* with Δ-values, defined by stimulated frequencies minus unstimulated frequencies. Functional markers with limited changes following Mtb-stimulation (i.e., Δ-values of magnitude ≤ 0.1% in over 1/3^rd^ of participants) were excluded from testing due to their low variability among our participants and tendency to recapitulate unstimulated tests. Significant between-group differences in Mtb-memory were based on FDR < 0.05.

These modeling procedures was also repeated for the TB-resister versus BCG recipient comparisons and the LTBI-participants versus BCG recipient comparisons. All analyses were performed in the R statistical package v4.0.2 unless otherwise specified. Additional methodical details and analysis code is publicly available at https://github.com/chooliu/Sterile_vs_Latent_TB_Immunity.

## Data Availability

• Data: the published article and supplemental information include all data generated and analyzed during this study. • Any additional information required to reanalyze the data reported in this paper is available from the lead contact upon request. • Code is available at https://github.com/chooliu/Sterile_vs_Latent_TB_Immunity.

## Supplemental information

Document S1. Figures S1–S22 and Tabels S1–S10
