# Supplementary material for: Comparative immune responses to Mycobacterium tuberculosis in people with latent infection or sterilizing protection
Source: iScience. 2023 Jul 20;26(8):107425. doi: 10.1016/j.isci.2023.107425 (PMC10410524; doi:10.1016/j.isci.2023.107425)
Supplement: Document S1. Figures S1–S22 and Tabels S1–S10 [file mmc1.pdf]

iScience, Volume *26*

Supplemental information

Comparative immune responses to *Mycobacterium tuberculosis* in people with latent infection or sterilizing protection

Emilie Jalbert, Cuining Liu, Vidya Mave, Nancy Lang, Anju Kagal, Chhaya Valvi, Mandar Paradkar, Nikhil Gupte, Rahul Lokhande, Renu Bharadwaj, Vandana Kulkarni, Amita Gupta, and Adriana Weinberg

Table S1. Flow cytometry panels related to Fig 1, 2, 3, 4, 5, 6, S1, S2, S3, S4, S5 and S6 and Tables S2, S3, S4, S5, and S6.

Panel	Cell type	Lineage markers	Functional markers
T cells	Tconv	CD3^{+}TCRγδ^{-} Vα24-Jα18^{-}Vα7.2^{-} CD56^{-}	CD25, CD69, CD107a, GranzB, GMCSF, IL2, IL10, IL17, IFNγ, TNFα, Ki67, and PD1
	NK	CD3^{-}CD16^{+} and/or CD56^{+}	
	γδT	CD3^{+}TCRγδ^{+}	
	iNKT	CD3^{+}Vα24-Jα18^{+}	
	NKT	CD3^{+}TCRγδ^{-} Vα24-Jα18^{-}Vα7.2^{-}CD56^{+}	
	MAIT	CD3^{+}TCR Vα7.2^{+}MR1-tetramer$^{+/-}$	
	GEMT	CD3^{+}Vα7.2^{+}GMM-CD1b-tetramer^{+}	
APC	*Monocyte*	Lin^{-}HLADR^{+}CD14^{+}CD16$^{-/+}$	CD40, CD80, CD83, IL1β, IL8, IL10, IL12p40, IL27, TNFα, ΠDL1
	cDC1	Lin^{-}HLADR^{+}CD14^{-}CD123^{-}CD11c^{+}CD141^{+}CD1c^{-}	
	cDC2	Lin^{-}HLADR^{+}CD14^{-}CD123^{-}CD11c^{+}CD141^{-}CD1c^{+}	
	pDC	Lin^{-}HLADR^{+}CD14^{-}CD123^{-}CD11c^{-}CD123^{+}	

Abbreviations: cDC= conventional dendritic cells; pDC= plasmacytoid DC; mono= monocytes; GEMT= germ-line encoded, mycoyl-reactive T cells; iNKT= invariant natural killer T cells; MAIT=mucosal-associated invariant T cells; NK= natural killer cells; NKT=natural killer T cells; Tconv=conventional T cells.

Table S2. Frequency of cell subsets in LTBI-participants and TB-resisters in unstimulated conditions related to Fig 1 and S2

Cell Subset	Marker	LTBI	Resister	Odds Ratio	p value	FDR p
cDC1	CD40	74.80 (54.99-76.98)*	82.30 (71.90-89.40)	0.55 (0.20 - 1.51)**	0.25	0.45
cDC1	CD80	30.20 (22.12-40.85)	12.70 (5.48-19.40)	2.82 (1.16 - 6.82)	0.022	0.13
cDC1	CD83	10.63 (1.97-23.82)	39.60 (14.95-44.35)	0.53 (0.23 - 1.20)	0.13	0.31
cDC1	IL10	2.10 (0.00-4.09)	3.17 (1.90-4.16)	0.81 (0.53 - 1.23)	0.32	0.53
cDC1	IL1β	5.52 (3.94-11.47)	15.40 (7.62-32.95)	0.47 (0.24 - 0.93)	0.029	0.15
cDC1	IL27	6.88 (0.00-17.77)	20.60 (8.08-35.80)	0.60 (0.27 - 1.34)	0.21	0.41
cDC1	IL8	27.60 (20.42-43.88)	39.20 (25.95-58.20)	0.54 (0.26 - 1.12)	0.096	0.26
cDC1	PDL1	88.45 (64.15-94.30)	87.50 (80.75-93.15)	0.66 (0.26 - 1.69)	0.39	0.60
cDC2	CD40	48.55 (42.05-63.88)	77.90 (67.55-89.85)	0.35 (0.17 - 0.75)	0.0071	0.065
cDC2	CD80	3.47 (1.41-6.19)	3.44 (1.81-3.89)	1.24 (0.78 - 1.96)	0.36	0.56
cDC2	CD83	12.55 (4.43-23.78)	26.60 (19.80-43.95)	0.54 (0.26 - 1.12)	0.10	0.27
cDC2	IL1β	3.60 (3.20-9.98)	9.46 (8.43-21.40)	0.61 (0.27 - 1.37)	0.23	0.44
cDC2	IL27	8.65 (2.79-11.67)	19.10 (16.00-35.85)	0.47 (0.23 - 0.99)	0.047	0.19
cDC2	IL8	32.75 (22.40-35.73)	37.80 (31.70-51.05)	0.62 (0.32 - 1.22)	0.17	0.37
cDC2	PDL1	73.40 (54.15-80.90)	84.00 (79.30-91.95)	0.43 (0.23 - 0.81)	0.0087	0.067
Mono	CD40	27.60 (23.95-37.15)	50.60 (44.35-65.40)	0.60 (0.30 - 1.21)	0.15	0.35
Mono	CD80	8.91 (4.77-11.88)	10.20 (6.51-12.20)	0.79 (0.56 - 1.12)	0.18	0.38
Mono	CD83	1.48 (1.24-5.40)	1.99 (1.78-4.54)	0.95 (0.44 - 2.07)	0.90	0.99
Mono	GMCSF	0.15 (0.11-0.25)	0.14 (0.11-0.17)	0.91 (0.75 - 1.11)	0.36	0.56
Mono	IL10	0.30 (0.14-0.48)	0.30 (0.14-0.80)	0.82 (0.54 - 1.25)	0.36	0.56
Mono	IL12p40	0.03 (0.00-0.24)	0.20 (0.08-0.35)	0.94 (0.88 - 1.01)	0.11	0.29
Mono	IL1β	15.50 (13.15-26.35)	66.40 (43.65-78.15)	0.31 (0.15 - 0.63)	0.0013	0.017
Mono	IL27	0.71 (0.32-1.16)	0.50 (0.26-1.06)	0.92 (0.70 - 1.21)	0.56	0.76
Mono	IL8	67.30 (62.27-79.88)	86.60 (80.20-88.40)	0.55 (0.29 - 1.03)	0.061	0.19
Mono	PDL1	52.75 (42.25-67.42)	81.50 (67.25-83.50)	0.45 (0.25 - 0.82)	0.0086	0.067
Mono	TNFα	0.15 (0.05-0.21)	0.04 (0.03-0.69)	0.91 (0.76 - 1.09)	0.32	0.53
pDC	CD40	29.90 (25.00-31.85)	51.50 (39.85-59.45)	0.35 (0.23 - 0.55)	5e-06	0.00022
pDC	CD80	1.05 (0.00-2.12)	1.20 (0.56-1.84)	1.01 (0.77 - 1.31)	0.96	1.00
pDC	CD83	15.95 (6.11-23.85)	20.60 (15.90-29.40)	0.56 (0.32 - 0.98)	0.042	0.19
pDC	IL10	0.81 (0.43-5.25)	1.25 (0.78-2.64)	1.00 (0.74 - 1.34)	1.00	1.00
pDC	IL1β	1.50 (0.00-2.83)	0.71 (0.49-2.54)	1.07 (0.79 - 1.45)	0.67	0.83
pDC	IL27	8.07 (6.45-14.87)	10.90 (7.19-12.70)	0.83 (0.55 - 1.25)	0.37	0.57
pDC	IL8	2.72 (2.14-6.49)	4.00 (3.65-8.07)	0.93 (0.58 - 1.50)	0.77	0.91
pDC	PDL1	15.55 (9.29-23.77)	22.20 (16.80-30.35)	0.60 (0.25 - 1.41)	0.24	0.45
iNKT CD4+	CD107a	3.01 (1.77-8.87)	4.49 (3.76-5.02)	1.04 (0.67 - 1.62)	0.85	0.97
iNKT CD4+	CD25	5.92 (2.67-6.13)	5.16 (3.98-6.43)	0.92 (0.65 - 1.28)	0.61	0.80
iNKT CD4+	CD69	39.90 (36.30-54.70)	45.70 (37.00-59.30)	0.91 (0.57 - 1.44)	0.69	0.85
iNKT CD4+	GranzB	20.10 (18.70-22.20)	30.50 (20.70-37.60)	0.61 (0.41 - 0.90)	0.012	0.09
iNKT CD4+	IL2	0.07 (0.00-0.12)	0.26 (0.11-0.38)	0.88 (0.76 - 1.02)	0.081	0.23
iNKT CD4+	Ki67	3.12 (1.72-4.00)	3.83 (3.56-5.40)	0.80 (0.63 - 1.01)	0.057	0.19
iNKT CD4+	PD1	24.40 (19.40-30.60)	35.10 (30.20-46.30)	0.65 (0.41 - 1.03)	0.064	0.20
iNKT CD4+	TNFα	0.18 (0.12-0.63)	0.31 (0.26-0.73)	0.97 (0.84 - 1.11)	0.65	0.82
iNKT CD8+	CD107a	5.22 (2.29-9.22)	2.20 (1.87-2.38)	1.63 (1.02 - 2.61)	0.041	0.19
iNKT CD8+	CD25	3.77 (0.85-4.48)	0.66 (0.49-0.96)	1.52 (1.12 - 2.07)	0.007	0.065
iNKT CD8+	CD69	39.00 (29.10-46.10)	33.70 (20.80-41.20)	1.21 (0.71 - 2.05)	0.48	0.68
iNKT CD8+	GranzB	57.40 (39.20-63.20)	90.60 (83.50-94.20)	0.21 (0.11 - 0.38)	4.8e-07	6.3e-05
iNKT CD8+	IL10	0.30 (0.14-3.17)	0.28 (0.14-0.48)	1.34 (1.00 - 1.79)	0.048	0.19
iNKT CD8+	IL17	0.30 (0.10-0.86)	0.20 (0.14-0.39)	1.23 (1.02 - 1.47)	0.027	0.15
iNKT CD8+	IL2	0.22 (0.00-1.09)	0.28 (0.17-0.41)	1.08 (0.87 - 1.36)	0.49	0.68
iNKT CD8+	Ki67	2.31 (1.78-3.65)	2.75 (1.34-3.59)	1.00 (0.80 - 1.26)	0.99	1.00
iNKT CD8+	PD1	23.10 (22.20-27.30)	34.20 (29.20-50.70)	0.54 (0.39 - 0.74)	0.00016	0.003
iNKT CD8+	TNFα	0.36 (0.00-0.58)	0.16 (0.07-0.24)	1.07 (1.00 - 1.16)	0.061	0.19
MAIT MR1-	CD107a	0.67 (0.46-1.44)	0.29 (0.27-0.45)	1.14 (0.99 - 1.31)	0.067	0.20

**MAIT MR1-**	CD25	4.31 (2.73-4.53)	2.55 (0.57-3.35)	1.32 (1.02 - 1.71)	0.038	0.19
**MAIT MR1-**	CD69	50.80 (37.20-67.70)	58.10 (25.50-58.90)	1.23 (0.61 - 2.50)	0.56	0.76
**MAIT MR1-**	GranzB	2.71 (0.92-9.26)	32.10 (26.70-88.00)	0.29 (0.11 - 0.71)	0.0074	0.065
**MAIT MR1-**	IL10	0.06 (0.02-0.09)	0.16 (0.06-0.24)	0.97 (0.94 - 1.00)	0.061	0.19
**MAIT MR1-**	IL2	0.05 (0.02-0.09)	0.03 (0.01-0.10)	1.01 (0.97 - 1.04)	0.72	0.87
**MAIT MR1-**	Ki67	0.37 (0.19-0.65)	0.65 (0.46-1.09)	0.84 (0.66 - 1.07)	0.17	0.37
**MAIT MR1-**	PD1	9.99 (9.58-19.10)	31.30 (23.60-32.60)	0.51 (0.35 - 0.74)	0.00037	0.006
**MAIT MR1-**	TNFα	0.03 (0.00-0.19)	0.06 (0.04-0.06)	1.01 (0.98 - 1.05)	0.45	0.65
**MAIT MR1+**	CD107a	1.13 (0.56-1.68)	0.33 (0.16-0.75)	1.13 (0.84 - 1.53)	0.42	0.61
**MAIT MR1+**	CD25	1.13 (0.64-1.68)	0.63 (0.29-1.40)	1.01 (0.67 - 1.53)	0.95	1.00
**MAIT MR1+**	CD69	82.90 (77.40-84.40)	90.90 (81.30-92.60)	0.64 (0.40 - 1.01)	0.057	0.19
**MAIT MR1+**	GranzB	0.67 (0.53-0.71)	1.17 (0.60-2.32)	0.82 (0.67 - 1.00)	0.049	0.19
**MAIT MR1+**	IFNγ	0.10 (0.00-0.35)	0.05 (0.01-0.07)	1.01 (0.78 - 1.30)	0.96	1.00
**MAIT MR1+**	IL10	0.44 (0.17-1.12)	0.16 (0.11-0.26)	0.99 (0.68 - 1.43)	0.95	1.00
**MAIT MR1+**	IL2	0.31 (0.05-0.54)	0.04 (0.01-0.11)	1.05 (0.85 - 1.28)	0.66	0.83
**MAIT MR1+**	Ki67	0.70 (0.26-1.35)	0.40 (0.32-0.48)	1.16 (1.05 - 1.28)	0.0029	0.034
**MAIT MR1+**	PD1	1.42 (0.70-4.73)	2.04 (1.60-2.57)	1.00 (0.74 - 1.36)	0.99	1.00
**NK**	CD107a	2.81 (1.91-4.02)	2.06 (1.68-3.98)	0.83 (0.49 - 1.41)	0.49	0.68
**NK**	CD25	2.18 (0.84-3.57)	2.85 (1.75-6.38)	0.83 (0.54 - 1.28)	0.40	0.60
**NK**	CD69	49.60 (37.80-65.40)	65.10 (53.50-76.40)	0.67 (0.37 - 1.22)	0.19	0.38
**NK**	GMCSF	0.10 (0.02-0.16)	0.05 (0.03-0.08)	1.01 (0.97 - 1.05)	0.76	0.91
**NK**	GranzB	82.70 (74.00-84.40)	79.10 (69.80-86.60)	1.13 (0.69 - 1.86)	0.62	0.80
**NK**	IFNγ	0.04 (0.03-0.46)	0.05 (0.03-0.07)	1.00 (0.80 - 1.25)	0.99	1.00
**NK**	IL10	0.06 (0.04-0.10)	0.07 (0.06-0.12)	0.99 (0.97 - 1.01)	0.29	0.53
**NK**	IL2	0.01 (0.00-0.07)	0.02 (0.01-0.03)	1.00 (0.98 - 1.03)	0.82	0.95
**NK**	Ki67	3.41 (2.46-4.83)	4.22 (3.69-5.29)	0.68 (0.49 - 0.95)	0.022	0.13
**NK**	PD1	1.55 (1.17-3.29)	2.31 (1.66-3.05)	0.89 (0.70 - 1.12)	0.30	0.53
**NK**	TNFα	0.05 (0.03-0.11)	0.07 (0.05-0.16)	0.99 (0.95 - 1.02)	0.36	0.56
**NKT CD4+**	CD107a	15.60 (7.31-21.80)	4.80 (2.37-17.20)	1.64 (0.92 - 2.92)	0.093	0.26
**NKT CD4+**	CD25	12.00 (6.53-14.20)	4.57 (4.20-6.81)	1.48 (0.99 - 2.22)	0.055	0.19
**NKT CD4+**	CD69	41.00 (35.60-46.80)	53.30 (26.80-66.00)	0.79 (0.46 - 1.36)	0.40	0.60
**NKT CD4+**	GranzB	31.70 (25.90-48.40)	41.60 (29.20-61.20)	0.62 (0.37 - 1.05)	0.076	0.23
**NKT CD4+**	Ki67	2.28 (1.46-2.70)	2.48 (1.58-2.92)	0.97 (0.75 - 1.27)	0.85	0.96
**NKT CD4+**	PD1	18.20 (9.42-26.40)	22.30 (17.40-25.00)	0.78 (0.55 - 1.12)	0.18	0.38
**NKT CD4+**	TNFα	0.00 (0.00-0.22)	0.08 (0.05-0.12)	1.00 (0.96 - 1.04)	0.90	0.99
**NKT CD8+**	CD107a	9.29 (5.11-29.00)	2.19 (1.09-4.35)	2.48 (1.29 - 4.76)	0.0064	0.065
**NKT CD8+**	CD25	4.13 (1.55-11.00)	0.90 (0.58-3.36)	1.87 (1.14 - 3.07)	0.014	0.094
**NKT CD8+**	CD69	31.30 (25.20-47.60)	24.50 (13.70-37.80)	1.35 (0.84 - 2.16)	0.21	0.41
**NKT CD8+**	GranzB	49.30 (47.10-71.80)	87.40 (82.40-91.10)	0.28 (0.16 - 0.49)	6.4e-06	0.00022
**NKT CD8+**	IL10	0.85 (0.45-5.36)	0.23 (0.10-0.54)	1.70 (0.94 - 3.07)	0.077	0.23
**NKT CD8+**	IL2	0.39 (0.23-4.64)	0.22 (0.12-0.40)	1.67 (0.98 - 2.84)	0.06	0.19
**NKT CD8+**	Ki67	1.32 (0.52-2.14)	1.33 (0.91-1.81)	0.90 (0.73 - 1.11)	0.34	0.56
**NKT CD8+**	PD1	10.10 (8.15-13.60)	9.10 (7.15-12.20)	1.01 (0.76 - 1.35)	0.96	1.00
**NKT CD8+**	TNFα	0.04 (0.00-0.15)	0.03 (0.02-0.05)	1.01 (0.99 - 1.04)	0.27	0.49
**Tconv CD4+**	CD107a	0.97 (0.66-1.34)	0.60 (0.48-0.83)	1.09 (0.97 - 1.21)	0.15	0.35
**Tconv CD4+**	CD25	11.60 (9.83-17.60)	12.90 (10.70-13.30)	1.02 (0.86 - 1.21)	0.84	0.96
**Tconv CD4+**	CD69	53.00 (45.80-67.00)	52.60 (50.00-62.70)	1.08 (0.64 - 1.82)	0.78	0.91
**Tconv CD4+**	GMCSF	0.01 (0.00-0.02)	0.02 (0.00-0.03)	1.00 (0.99 - 1.01)	0.88	0.99
**Tconv CD4+**	GranzB	1.64 (0.80-2.39)	4.02 (2.88-5.92)	0.56 (0.42 - 0.75)	0.00012	0.0027
**Tconv CD4+**	IFNγ	0.02 (0.01-0.03)	0.01 (0.01-0.01)	1.00 (0.99 - 1.01)	0.48	0.68
**Tconv CD4+**	IL10	0.02 (0.02-0.03)	0.03 (0.02-0.03)	1.00 (1.00 - 1.00)	0.19	0.38
**Tconv CD4+**	IL17	0.00 (0.00-0.01)	0.00 (0.00-0.00)	1.00 (1.00 - 1.00)	0.94	1.00
**Tconv CD4+**	IL2	0.01 (0.00-0.01)	0.00 (0.00-0.01)	1.00 (1.00 - 1.00)	0.93	1.00
**Tconv CD4+**	Ki67	0.52 (0.45-0.74)	0.76 (0.68-0.85)	0.89 (0.81 - 0.99)	0.029	0.15
**Tconv CD4+**	PD1	14.00 (13.00-15.00)	21.00 (18.30-25.00)	0.63 (0.51 - 0.77)	6.6e-06	0.00022
**Tconv CD4+**	TNFα	0.01 (0.01-0.01)	0.01 (0.01-0.01)	1.00 (1.00 - 1.00)	0.72	0.87
**Tconv CD8+**	CD107a	0.78 (0.55-1.02)	0.52 (0.39-0.68)	1.08 (0.98 - 1.19)	0.12	0.29

**Tconv CD8+**	CD25	0.71 (0.46-1.80)	0.80 (0.45-0.99)	1.19 (1.00 - 1.43)	0.053	0.19
**Tconv CD8+**	CD69	47.30 (34.30-65.80)	44.60 (33.10-57.70)	1.18 (0.69 - 2.02)	0.54	0.74
**Tconv CD8+**	GMCSF	0.01 (0.01-0.02)	0.01 (0.00-0.01)	1.00 (1.00 - 1.01)	0.022	0.13
**Tconv CD8+**	GranzB	15.10 (11.70-19.70)	37.50 (34.60-61.70)	0.29 (0.17 - 0.51)	1.4e-05	0.00038
**Tconv CD8+**	IFNγ	0.02 (0.02-0.04)	0.01 (0.01-0.01)	1.01 (1.00 - 1.03)	0.18	0.38
**Tconv CD8+**	IL10	0.03 (0.02-0.05)	0.03 (0.03-0.03)	1.00 (1.00 - 1.01)	0.59	0.78
**Tconv CD8+**	IL2	0.03 (0.01-0.06)	0.01 (0.01-0.02)	1.01 (0.99 - 1.02)	0.31	0.53
**Tconv CD8+**	Ki67	0.39 (0.35-0.45)	0.52 (0.49-0.73)	0.88 (0.77 - 1.00)	0.043	0.19
**Tconv CD8+**	PD1	10.10 (9.21-12.10)	18.50 (15.60-39.90)	0.46 (0.29 - 0.73)	0.00091	0.013
**Tconv CD8+**	TNFα	0.01 (0.01-0.01)	0.01 (0.01-0.02)	1.00 (1.00 - 1.00)	0.62	0.80
**γδT**	CD107a	0.84 (0.71-20.00)	0.42 (0.34-0.53)	1.76 (0.88 - 3.53)	0.11	0.28
**γδT**	CD25	0.39 (0.22-15.00)	0.30 (0.22-0.57)	1.41 (0.73 - 2.70)	0.30	0.53
**γδT**	CD69	62.60 (47.80-70.10)	53.20 (48.60-63.90)	1.33 (0.85 - 2.09)	0.22	0.41
**γδT**	GranzB	49.90 (26.70-60.50)	39.80 (35.10-50.90)	1.14 (0.71 - 1.83)	0.58	0.78
**γδT**	IFNγ	0.07 (0.04-7.11)	0.04 (0.02-0.06)	1.49 (0.82 - 2.69)	0.19	0.38
**γδT**	IL10	0.10 (0.06-0.16)	0.10 (0.08-0.16)	1.00 (0.98 - 1.03)	0.72	0.87
**γδT**	IL17	0.09 (0.03-0.22)	0.10 (0.03-0.14)	1.04 (0.99 - 1.10)	0.14	0.35
**γδT**	IL2	0.02 (0.00-0.04)	0.01 (0.01-0.02)	1.00 (0.97 - 1.03)	0.97	1.00
**γδT**	Ki67	0.96 (0.44-1.17)	0.77 (0.59-1.46)	0.87 (0.71 - 1.06)	0.16	0.37
**γδT**	PD1	12.80 (7.70-41.40)	14.40 (12.30-16.50)	1.27 (0.77 - 2.09)	0.35	0.56
**γδT**	TNFα	0.08 (0.03-0.47)	0.03 (0.01-0.10)	1.08 (1.00 - 1.17)	0.04	0.19

* Median (quartiles)
**Point estimate (95% Confidence interval)

Table S3. Frequency of cell subsets in LTBI-participants and TB-resisters in Mtb-stimulated conditions related to Fig 2 and S3

Cell Subset	Marker	LTBI	Resister	Odds Ratio	p value	FDR p
cDC1	CD40	91.50 (85.97, 96.03)*	89.20 (86.25, 93.30)	0.83 (0.32 - 2.14)**	0.70	0.78
cDC1	CD80	7.79 (3.20, 10.23)	13.90 (9.42, 16.30)	0.56 (0.34 - 0.91)	0.02	0.094
cDC1	CD83	48.75 (29.25, 56.68)	43.60 (36.90, 49.15)	0.61 (0.29 - 1.28)	0.19	0.36
cDC1	GMCSF	0.36 (0.00, 1.13)	1.00 (0.41, 1.42)	0.99 (0.68 - 1.44)	0.96	0.98
cDC1	IL10	0.78 (0.00, 3.48)	1.65 (1.05, 3.52)	0.83 (0.54 - 1.26)	0.38	0.53
cDC1	IL1β	72.85 (63.38, 76.58)	57.30 (51.45, 79.50)	0.69 (0.32 - 1.47)	0.34	0.52
cDC1	IL27	55.20 (48.73, 58.43)	49.60 (44.10, 59.65)	1.12 (0.49 - 2.55)	0.79	0.86
cDC1	IL8	83.40 (78.42, 85.00)	70.90 (67.45, 82.25)	0.69 (0.30 - 1.62)	0.40	0.53
cDC1	PDL1	97.75 (88.45, 99.20)	98.60 (96.35, 99.40)	0.62 (0.23 - 1.65)	0.34	0.52
cDC1	TNFα	3.27 (0.00, 8.71)	1.28 (0.66, 2.38)	1.59 (1.04 - 2.43)	0.031	0.12
cDC2	CD40	87.95 (58.80, 88.70)	89.40 (87.45, 92.30)	0.41 (0.16 - 1.06)	0.065	0.19
cDC2	CD80	2.24 (0.80, 8.37)	5.81 (3.33, 7.83)	0.75 (0.51 - 1.11)	0.15	0.32
cDC2	CD83	29.70 (22.80, 35.02)	26.70 (24.90, 30.80)	0.95 (0.59 - 1.54)	0.84	0.87
cDC2	GMCSF	1.32 (0.56, 2.49)	1.09 (0.54, 1.82)	1.04 (0.73 - 1.47)	0.84	0.87
cDC2	IL10	2.58 (0.84, 3.82)	2.25 (0.46, 3.08)	0.89 (0.51 - 1.56)	0.68	0.77
cDC2	IL12p40	0.88 (0.16, 1.92)	0.44 (0.17, 0.98)	1.18 (0.94 - 1.48)	0.16	0.32
cDC2	IL1β	59.90 (55.70, 64.28)	61.50 (54.80, 74.95)	0.87 (0.54 - 1.38)	0.55	0.69
cDC2	IL27	51.75 (47.60, 58.90)	56.10 (51.15, 59.70)	0.71 (0.38 - 1.31)	0.27	0.45
cDC2	IL8	85.85 (71.78, 87.40)	86.10 (81.75, 92.00)	0.65 (0.39 - 1.08)	0.095	0.24
cDC2	PDL1	92.65 (81.80, 95.57)	98.50 (96.00, 98.80)	0.50 (0.24 - 1.06)	0.071	0.21
cDC2	TNFα	3.50 (2.18, 8.02)	2.11 (1.09, 3.88)	1.23 (0.80 - 1.90)	0.34	0.52
Mono	CD40	62.00 (46.83, 76.25)	49.00 (43.15, 66.85)	1.15 (0.60 - 2.23)	0.68	0.77
Mono	CD80	1.06 (0.81, 2.07)	5.89 (2.65, 6.96)	0.59 (0.39 - 0.89)	0.012	0.061
Mono	CD83	15.90 (11.57, 19.38)	10.80 (5.71, 17.20)	1.26 (0.73 - 2.17)	0.40	0.53
Mono	GMCSF	0.54 (0.16, 0.69)	0.57 (0.26, 1.17)	0.87 (0.66 - 1.16)	0.36	0.52
Mono	IL10	1.04 (0.83, 1.39)	1.22 (0.94, 2.41)	0.79 (0.53 - 1.17)	0.24	0.42
Mono	IL1β	97.20 (88.45, 98.35)	98.00 (94.80, 98.20)	0.66 (0.27 - 1.64)	0.37	0.53
Mono	IL27	2.19 (1.44, 4.54)	2.08 (1.41, 3.03)	1.00 (0.71 - 1.41)	0.99	1.00
Mono	IL8	96.20 (89.70, 97.12)	97.10 (95.45, 97.80)	0.66 (0.28 - 1.57)	0.35	0.52
Mono	PDL1	98.30 (86.90, 99.15)	97.00 (94.50, 98.75)	0.80 (0.35 - 1.85)	0.60	0.71
Mono	TNFα	0.76 (0.58, 1.12)	0.62 (0.22, 0.90)	1.05 (0.91 - 1.22)	0.49	0.62
pDC	CD40	70.30 (49.52, 77.00)	73.10 (65.55, 76.15)	0.60 (0.33 - 1.10)	0.10	0.25
pDC	CD80	4.08 (3.67, 4.56)	2.79 (1.77, 3.27)	1.13 (0.84 - 1.52)	0.41	0.53
pDC	CD83	33.45 (13.24, 49.78)	28.20 (22.20, 34.45)	0.95 (0.54 - 1.66)	0.85	0.88
pDC	IL10	2.02 (0.62, 3.79)	1.14 (0.78, 2.32)	1.03 (0.79 - 1.35)	0.83	0.87
pDC	IL1β	15.90 (12.73, 20.27)	14.70 (9.66, 21.75)	0.92 (0.59 - 1.46)	0.74	0.81
pDC	IL27	25.45 (16.10, 28.27)	24.00 (16.85, 27.90)	0.81 (0.47 - 1.40)	0.45	0.58
pDC	IL8	22.70 (16.88, 26.62)	15.30 (12.80, 20.60)	1.35 (1.02 - 1.78)	0.034	0.12
pDC	PDL1	90.00 (69.10, 93.72)	72.40 (59.80, 78.25)	1.44 (0.64 - 3.20)	0.38	0.53
pDC	TNFα	0.72 (0.31, 0.79)	0.49 (0.00, 1.00)	0.98 (0.85 - 1.14)	0.84	0.87
iNKT CD4+	CD107a	6.15 (3.26, 14.50)	6.07 (3.40, 7.14)	1.28 (0.84 - 1.95)	0.24	0.42
iNKT CD4+	CD25	9.19 (7.37, 19.50)	8.08 (5.74, 9.43)	1.36 (0.88 - 2.12)	0.17	0.34
iNKT CD4+	CD69	50.40 (36.50, 53.80)	43.70 (32.90, 51.20)	1.25 (0.82 - 1.89)	0.30	0.47
iNKT CD4+	GMCSF	0.24 (0.13, 0.34)	0.22 (0.18, 0.66)	0.95 (0.81 - 1.13)	0.59	0.71
iNKT CD4+	GranzB	21.00 (18.90, 24.70)	24.20 (19.30, 40.20)	0.65 (0.42 - 1.00)	0.049	0.17
iNKT CD4+	IFNγ	0.99 (0.77, 11.80)	0.42 (0.13, 0.71)	1.94 (1.18 - 3.17)	0.0086	0.046
iNKT CD4+	Ki67	2.53 (2.25, 5.60)	3.86 (3.00, 4.92)	0.94 (0.73 - 1.20)	0.61	0.72
iNKT CD4+	PD1	29.70 (18.30, 44.80)	31.90 (27.10, 43.90)	0.87 (0.54 - 1.39)	0.55	0.69
iNKT CD4+	TNFα	0.56 (0.32, 0.80)	0.58 (0.14, 0.88)	1.18 (0.97 - 1.44)	0.10	0.25
iNKT CD8+	CD107a	12.00 (3.81, 16.70)	3.13 (2.47, 3.73)	1.90 (1.20 - 3.03)	0.0067	0.039
iNKT CD8+	CD25	7.39 (2.88, 9.45)	1.98 (1.55, 2.65)	1.94 (1.35 - 2.79)	0.00037	0.0045
iNKT CD8+	CD69	42.50 (38.40, 54.70)	34.80 (24.90, 41.70)	1.53 (1.00 - 2.34)	0.053	0.17
iNKT CD8+	GranzB	50.30 (34.50, 66.10)	85.60 (77.40, 92.60)	0.29 (0.15 - 0.55)	0.00016	0.0028

iNKT CD8+	IFNγ	1.08 (0.46, 2.80)	0.20 (0.19, 0.44)	1.50 (1.16 - 1.94)	0.0017	0.013
iNKT CD8+	IL10	2.30 (0.72, 9.09)	0.21 (0.04, 0.80)	1.94 (1.20 - 3.15)	0.0069	0.039
iNKT CD8+	IL17	0.69 (0.13, 2.80)	0.25 (0.19, 0.38)	1.32 (0.95 - 1.82)	0.095	0.24
iNKT CD8+	IL2	0.93 (0.30, 4.96)	0.25 (0.14, 0.57)	1.59 (1.04 - 2.44)	0.033	0.12
iNKT CD8+	Ki67	3.31 (2.10, 4.07)	1.62 (1.35, 2.91)	1.23 (1.00 - 1.52)	0.051	0.17
iNKT CD8+	PD1	29.10 (24.00, 34.80)	30.30 (24.80, 37.90)	0.81 (0.56 - 1.15)	0.24	0.42
MAIT MR1-	CD107a	1.18 (0.87, 1.56)	0.52 (0.33, 0.82)	1.23 (1.03 - 1.48)	0.022	0.096
MAIT MR1-	CD25	6.21 (4.41, 7.93)	3.69 (1.22, 5.11)	1.52 (1.13 - 2.03)	0.0053	0.036
MAIT MR1-	CD69	52.90 (44.90, 64.60)	54.90 (27.30, 61.50)	1.31 (0.69 - 2.49)	0.41	0.53
MAIT MR1-	GranzB	2.39 (1.73, 9.81)	36.60 (27.70, 87.40)	0.28 (0.11 - 0.70)	0.0066	0.039
MAIT MR1-	PD1	13.10 (11.70, 19.90)	31.70 (25.50, 34.70)	0.53 (0.36 - 0.77)	0.00098	0.011
MAIT MR1+	CD107a	3.40 (0.74, 4.77)	0.65 (0.58, 1.28)	1.39 (0.83 - 2.31)	0.21	0.38
MAIT MR1+	CD25	8.27 (3.18, 9.43)	3.48 (1.81, 8.13)	1.29 (0.81 - 2.05)	0.29	0.46
MAIT MR1+	CD69	85.10 (83.30, 86.80)	90.80 (84.20, 94.80)	0.77 (0.52 - 1.14)	0.19	0.36
MAIT MR1+	GranzB	2.55 (1.28, 5.03)	3.10 (0.92, 4.69)	1.12 (0.76 - 1.65)	0.57	0.69
MAIT MR1+	IFNγ	1.85 (0.86, 7.31)	0.68 (0.43, 2.17)	1.59 (0.97 - 2.59)	0.063	0.19
MAIT MR1+	PD1	1.16 (0.77, 6.05)	3.09 (2.54, 4.54)	0.90 (0.64 - 1.28)	0.57	0.69
NK	CD107a	3.99 (3.28, 5.97)	6.26 (3.61, 10.60)	0.63 (0.34 - 1.16)	0.14	0.31
NK	CD25	4.96 (3.58, 7.80)	8.46 (4.58, 12.70)	0.71 (0.46 - 1.10)	0.13	0.29
NK	CD69	71.10 (57.50, 81.20)	78.10 (68.30, 84.00)	0.69 (0.42 - 1.13)	0.14	0.31
NK	GranzB	82.00 (80.20, 83.60)	76.50 (69.20, 83.70)	1.47 (0.85 - 2.54)	0.17	0.34
NK	IFNγ	2.03 (1.13, 3.90)	1.42 (0.52, 3.23)	1.17 (0.81 - 1.70)	0.40	0.53
NK	Ki67	3.29 (2.64, 4.63)	3.59 (3.46, 4.56)	0.78 (0.58 - 1.03)	0.084	0.23
NK	PD1	3.83 (2.93, 4.74)	4.69 (3.33, 6.23)	0.85 (0.68 - 1.05)	0.14	0.31
NKT CD4+	CD107a	18.00 (11.80, 30.90)	17.60 (5.05, 41.20)	1.14 (0.65 - 1.98)	0.65	0.75
NKT CD4+	CD25	23.60 (14.20, 28.10)	13.40 (8.19, 15.60)	1.66 (1.22 - 2.26)	0.0014	0.013
NKT CD4+	CD69	50.00 (44.40, 58.60)	56.20 (35.40, 69.70)	1.00 (0.63 - 1.59)	1.00	1.00
NKT CD4+	GranzB	30.00 (22.20, 40.20)	37.90 (27.50, 56.20)	0.61 (0.36 - 1.02)	0.058	0.18
NKT CD4+	IFNγ	2.04 (1.69, 4.03)	1.10 (0.59, 2.96)	1.28 (0.97 - 1.69)	0.08	0.22
NKT CD4+	Ki67	1.80 (1.62, 2.77)	2.58 (1.79, 3.26)	0.87 (0.71 - 1.06)	0.17	0.34
NKT CD4+	PD1	22.70 (10.10, 24.20)	23.70 (17.40, 26.00)	0.83 (0.59 - 1.16)	0.28	0.46
NKT CD8+	CD107a	21.50 (17.40, 38.90)	3.90 (1.78, 7.85)	3.67 (1.94 - 6.93)	6.3e-05	0.0017
NKT CD8+	CD25	11.90 (8.75, 23.30)	2.46 (1.71, 6.36)	2.61 (1.60 - 4.26)	0.00012	0.0026
NKT CD8+	CD69	33.40 (29.50, 50.20)	31.30 (18.60, 38.20)	1.39 (0.92 - 2.08)	0.11	0.27
NKT CD8+	GranzB	48.60 (24.70, 67.00)	85.00 (74.60, 89.00)	0.25 (0.13 - 0.46)	1.1e-05	0.0011
NKT CD8+	IL10	2.56 (0.74, 10.90)	0.43 (0.28, 0.92)	2.04 (1.11 - 3.74)	0.021	0.095
NKT CD8+	IL2	1.32 (0.45, 10.90)	0.41 (0.24, 0.65)	1.92 (1.08 - 3.40)	0.025	0.10
NKT CD8+	Ki67	1.32 (0.84, 1.69)	1.27 (0.88, 2.03)	0.96 (0.82 - 1.13)	0.65	0.75
NKT CD8+	PD1	9.96 (6.43, 15.60)	9.92 (7.83, 12.00)	0.96 (0.71 - 1.29)	0.78	0.85
Tconv CD4+	CD107a	1.47 (1.17, 1.72)	0.96 (0.84, 1.18)	1.13 (1.02 - 1.26)	0.015	0.076
Tconv CD4+	CD25	20.80 (18.80, 26.20)	15.70 (14.60, 17.60)	1.31 (1.09 - 1.57)	0.0035	0.025
Tconv CD4+	CD69	60.50 (54.80, 67.10)	55.00 (47.90, 62.40)	1.32 (0.87 - 2.03)	0.20	0.36
Tconv CD4+	GranzB	2.10 (0.84, 2.96)	4.34 (3.20, 6.08)	0.58 (0.43 - 0.78)	0.00034	0.0045
Tconv CD4+	PD1	16.40 (16.10, 16.90)	22.70 (20.60, 26.40)	0.65 (0.53 - 0.80)	4.5e-05	0.0016
Tconv CD8+	CD25	2.55 (2.16, 4.05)	1.18 (0.72, 1.58)	1.51 (1.20 - 1.90)	0.00037	0.0045
Tconv CD8+	CD69	52.40 (43.00, 64.90)	43.50 (36.20, 54.50)	1.30 (0.82 - 2.07)	0.26	0.44
Tconv CD8+	GranzB	17.10 (13.20, 20.70)	37.60 (35.00, 62.60)	0.31 (0.17 - 0.53)	3.2e-05	0.0016
Tconv CD8+	PD1	10.80 (10.30, 13.80)	20.60 (16.80, 40.90)	0.47 (0.30 - 0.76)	0.0017	0.013
γδT	CD107a	2.99 (1.15, 24.50)	0.89 (0.62, 0.95)	1.93 (0.93 - 3.98)	0.076	0.22
γδT	CD25	3.64 (2.46, 17.80)	1.74 (1.44, 3.26)	1.62 (0.90 - 2.91)	0.11	0.26
γδT	CD69	66.70 (63.50, 78.30)	58.60 (54.70, 65.80)	1.72 (1.23 - 2.39)	0.0014	0.013
γδT	GranzB	57.30 (30.70, 65.00)	40.50 (36.60, 49.50)	1.25 (0.78 - 2.02)	0.36	0.52
γδT	IFNγ	4.53 (1.94, 10.00)	0.87 (0.34, 1.31)	1.94 (1.09 - 3.47)	0.025	0.10
γδT	Ki67	0.78 (0.65, 1.32)	0.88 (0.71, 1.34)	0.93 (0.77 - 1.11)	0.40	0.53
γδT	PD1	14.40 (14.20, 45.70)	17.70 (15.10, 18.40)	1.34 (0.83 - 2.17)	0.23	0.42

* Median (quartiles)

**Point estimate (95% Confidence interval)

Table S4. Frequency of cell subsets in LTBI-participants and TB-resisters in Mtb-stimulated conditions after background subtraction related to Fig 3 and S4

Cell Subset	Marker	LTBI	Resister	Odds Ratio	p value	FDR p
cDC1	CD40	16.55 (8.55, 20.22)*	10.40 (3.75, 15.95)	1.05 (0.41 - 2.71)**	0.91	0.95
cDC1	CD80	-23.93 (-33.01, -8.94)	1.10 (-5.20, 4.54)	0.65 (0.37 - 1.14)	0.13	0.31
cDC1	CD83	16.20 (0.00, 44.73)	12.10 (1.50, 23.40)	0.81 (0.39 - 1.67)	0.56	0.74
cDC1	GMCSF	0.36 (0.00, 1.13)	0.42 (0.00, 1.17)	1.12 (0.79 - 1.60)	0.52	0.72
cDC1	IL10	-0.74 (-2.16, 0.05)	-0.49 (-2.09, 0.18)	0.91 (0.67 - 1.24)	0.55	0.74
cDC1	IL1β	65.72 (51.88, 70.05)	40.60 (34.80, 63.92)	0.85 (0.39 - 1.87)	0.69	0.84
cDC1	IL27	38.66 (18.08, 51.90)	29.00 (24.70, 39.35)	1.15 (0.49 - 2.69)	0.75	0.88
cDC1	IL8	51.75 (19.32, 62.60)	27.20 (23.45, 51.20)	0.96 (0.44 - 2.08)	0.92	0.95
cDC1	PDL1	6.65 (2.48, 13.68)	12.50 (6.05, 17.45)	0.69 (0.36 - 1.33)	0.27	0.49
cDC1	TNFα	3.27 (0.00, 8.63)	0.49 (0.20, 1.54)	1.72 (1.14 - 2.59)	0.01	0.072
cDC2	CD40	28.10 (11.02, 44.30)	13.60 (5.70, 23.80)	0.54 (0.19 - 1.56)	0.26	0.48
cDC2	CD80	-0.89 (-1.73, 0.58)	2.37 (0.62, 4.53)	0.62 (0.44 - 0.89)	0.01	0.072
cDC2	CD83	19.30 (5.12, 24.84)	-3.30 (-8.45, 6.50)	1.27 (0.79 - 2.03)	0.32	0.55
cDC2	GMCSF	0.88 (0.51, 1.86)	0.40 (0.30, 1.40)	1.10 (0.81 - 1.49)	0.53	0.73
cDC2	IL10	1.13 (0.00, 2.45)	0.52 (0.38, 2.18)	1.10 (0.85 - 1.44)	0.46	0.68
cDC2	IL12p40	0.88 (0.16, 1.92)	0.34 (0.13, 0.98)	1.17 (0.92 - 1.48)	0.19	0.40
cDC2	IL1β	55.20 (43.85, 57.11)	39.96 (33.16, 51.89)	1.04 (0.70 - 1.54)	0.86	0.93
cDC2	IL27	41.64 (25.97, 50.52)	27.60 (22.15, 33.65)	0.94 (0.50 - 1.76)	0.85	0.93
cDC2	IL8	48.25 (29.53, 61.38)	48.50 (35.60, 55.35)	0.73 (0.46 - 1.16)	0.18	0.39
cDC2	PDL1	20.10 (9.13, 25.78)	15.00 (6.85, 17.50)	0.75 (0.44 - 1.31)	0.31	0.55
cDC2	TNFα	2.94 (1.71, 8.02)	0.97 (0.54, 3.40)	1.35 (0.90 - 2.02)	0.15	0.33
Mono	CD40	27.35 (9.18, 31.90)	2.30 (-5.30, 6.00)	2.02 (1.23 - 3.34)	0.0058	0.069
Mono	CD80	-7.81 (-9.62, -2.86)	-4.68 (-7.37, -1.13)	0.61 (0.40 - 0.92)	0.017	0.081
Mono	CD83	10.20 (8.74, 14.19)	3.98 (3.04, 9.22)	1.48 (1.12 - 1.95)	0.0063	0.069
Mono	GMCSF	0.21 (0.04, 0.50)	0.50 (0.13, 1.02)	1.00 (0.86 - 1.16)	0.99	0.99
Mono	IL10	0.86 (0.45, 1.15)	0.82 (0.57, 1.27)	0.93 (0.78 - 1.11)	0.41	0.65
Mono	IL1β	77.00 (32.65, 84.03)	28.50 (18.05, 55.15)	0.77 (0.25 - 2.37)	0.65	0.80
Mono	IL27	1.77 (0.81, 3.31)	1.05 (0.88, 2.40)	1.01 (0.71 - 1.44)	0.94	0.95
Mono	IL8	20.40 (4.38, 28.62)	8.30 (4.90, 15.70)	0.97 (0.47 - 2.02)	0.94	0.95
Mono	PDL1	36.40 (17.62, 45.42)	16.50 (9.60, 31.30)	1.19 (0.46 - 3.05)	0.72	0.87
Mono	TNFα	0.58 (0.34, 1.05)	0.16 (-0.11, 0.44)	1.12 (0.99 - 1.26)	0.073	0.21
pDC	CD40	38.35 (16.57, 49.45)	18.60 (15.40, 26.85)	1.37 (0.62 - 3.02)	0.44	0.68
pDC	CD80	2.99 (1.99, 3.55)	1.58 (0.43, 2.55)	1.15 (0.86 - 1.53)	0.34	0.57
pDC	CD83	12.79 (5.67, 23.53)	8.10 (0.25, 12.00)	1.36 (0.94 - 1.96)	0.099	0.24
pDC	IL10	-0.28 (-1.20, 0.21)	-0.26 (-0.52, 0.21)	0.98 (0.82 - 1.17)	0.83	0.93
pDC	IL1β	13.84 (10.29, 19.84)	14.03 (7.63, 21.55)	0.93 (0.60 - 1.45)	0.74	0.88
pDC	IL27	12.48 (8.84, 15.59)	14.12 (5.83, 17.01)	0.95 (0.62 - 1.47)	0.83	0.93
pDC	IL8	21.02 (11.41, 23.75)	11.30 (6.51, 14.53)	1.37 (1.06 - 1.76)	0.016	0.08
pDC	PDL1	70.81 (40.15, 77.17)	35.20 (25.75, 54.15)	1.85 (0.85 - 4.02)	0.12	0.28
pDC	TNFα	0.09 (-0.08, 0.76)	0.49 (-0.09, 0.95)	0.99 (0.85 - 1.14)	0.84	0.93
iNKT CD4+	CD107a	0.46 (-0.40, 2.82)	0.28 (-0.28, 2.71)	1.12 (0.80 - 1.58)	0.52	0.72
iNKT CD4+	CD25	2.38 (0.10, 16.27)	1.87 (0.45, 4.94)	1.39 (0.90 - 2.17)	0.14	0.31
iNKT CD4+	CD69	0.80 (-1.60, 4.80)	-5.00 (-5.90, 0.00)	1.35 (1.06 - 1.72)	0.013	0.077
iNKT CD4+	GMCSF	0.13 (0.00, 0.19)	0.00 (-0.21, 0.20)	1.06 (0.91 - 1.25)	0.45	0.68
iNKT CD4+	GranzB	0.30 (-2.40, 1.60)	2.40 (-3.60, 3.70)	1.07 (0.85 - 1.36)	0.57	0.74
iNKT CD4+	IFNγ	0.68 (0.24, 11.80)	0.33 (0.00, 0.55)	1.87 (1.16 - 3.03)	0.011	0.072
iNKT CD4+	Ki67	0.80 (0.06, 0.81)	-0.12 (-0.98, 0.54)	1.07 (0.84 - 1.36)	0.57	0.74
iNKT CD4+	PD1	-0.70 (-1.10, 3.49)	-3.20 (-10.70, -1.30)	1.18 (0.86 - 1.61)	0.30	0.53
iNKT CD4+	TNFα	0.25 (0.15, 0.60)	0.04 (-0.17, 0.15)	1.19 (1.01 - 1.40)	0.035	0.12
iNKT CD8+	CD107a	2.01 (0.78, 6.78)	0.91 (0.26, 1.28)	1.40 (1.00 - 1.97)	0.053	0.17
iNKT CD8+	CD25	2.29 (0.50, 5.44)	1.06 (0.89, 1.68)	1.51 (1.04 - 2.19)	0.03	0.11
iNKT CD8+	CD69	5.00 (-1.60, 9.30)	3.20 (-6.20, 4.50)	1.38 (1.09 - 1.75)	0.0085	0.072
iNKT CD8+	GranzB	-4.10 (-5.90, 9.10)	-2.80 (-5.00, -0.50)	1.20 (0.82 - 1.75)	0.35	0.59

iNKT CD8+	IFNγ	0.94 (-0.24, 2.80)	0.19 (-0.03, 0.39)	1.52 (1.18 - 1.95)	0.001	0.022
iNKT CD8+	IL10	1.21 (0.57, 4.61)	0.08 (-0.12, 0.23)	1.19 (0.89 - 1.57)	0.24	0.46
iNKT CD8+	IL17	0.13 (-0.10, 0.55)	0.00 (-0.08, 0.18)	1.15 (0.82 - 1.60)	0.42	0.66
iNKT CD8+	IL2	0.73 (0.30, 2.72)	0.09 (-0.03, 0.12)	1.41 (1.05 - 1.90)	0.021	0.089
iNKT CD8+	Ki67	0.68 (0.37, 1.07)	0.03 (-0.54, 0.16)	1.26 (1.06 - 1.49)	0.008	0.072
iNKT CD8+	PD1	1.90 (-0.30, 9.60)	-3.60 (-6.90, -1.00)	1.39 (1.03 - 1.88)	0.03	0.11
MAIT MR1-	CD107a	0.52 (0.09, 1.01)	0.25 (0.04, 0.46)	1.11 (0.95 - 1.30)	0.19	0.39
MAIT MR1-	CD25	2.38 (1.11, 2.97)	0.57 (0.32, 1.65)	1.22 (1.03 - 1.44)	0.024	0.091
MAIT MR1-	CD69	1.40 (-2.90, 5.10)	-0.20 (-2.10, 1.80)	1.14 (1.01 - 1.29)	0.033	0.12
MAIT MR1-	GranzB	0.56 (-0.07, 1.30)	1.00 (0.40, 1.50)	0.69 (0.52 - 0.93)	0.013	0.077
MAIT MR1-	PD1	1.10 (0.16, 2.12)	1.60 (0.40, 2.70)	1.00 (0.92 - 1.10)	0.93	0.95
MAIT MR1+	CD107a	0.69 (0.28, 3.09)	0.45 (0.22, 0.64)	1.33 (1.05 - 1.67)	0.018	0.081
MAIT MR1+	CD25	2.23 (1.65, 8.62)	2.18 (1.30, 3.14)	1.29 (0.85 - 1.96)	0.23	0.46
MAIT MR1+	CD69	2.40 (0.50, 7.70)	0.60 (-1.00, 3.10)	1.10 (0.83 - 1.45)	0.50	0.72
MAIT MR1+	GranzB	1.23 (0.31, 4.42)	0.79 (0.27, 2.01)	1.20 (0.79 - 1.84)	0.39	0.63
MAIT MR1+	IFNγ	0.69 (0.47, 7.31)	0.46 (0.25, 0.80)	1.59 (0.98 - 2.59)	0.063	0.19
MAIT MR1+	PD1	0.68 (-0.20, 0.81)	1.19 (0.35, 1.39)	0.82 (0.72 - 0.94)	0.0033	0.05
NK	CD107a	1.37 (1.22, 3.16)	4.14 (2.28, 6.79)	0.75 (0.54 - 1.05)	0.094	0.24
NK	CD25	2.97 (2.18, 4.12)	5.61 (2.95, 8.61)	0.76 (0.56 - 1.05)	0.092	0.24
NK	CD69	14.10 (-3.50, 21.50)	11.80 (1.90, 16.60)	0.96 (0.68 - 1.34)	0.79	0.92
NK	GranzB	1.60 (-1.60, 4.80)	-2.70 (-6.30, 0.80)	1.34 (1.04 - 1.72)	0.024	0.091
NK	IFNγ	1.29 (0.85, 3.90)	0.83 (0.29, 2.44)	1.18 (0.81 - 1.70)	0.39	0.63
NK	Ki67	0.10 (-0.11, 0.16)	-0.33 (-0.73, -0.23)	1.02 (0.92 - 1.14)	0.66	0.81
NK	PD1	1.27 (0.45, 1.76)	2.14 (1.37, 2.64)	0.89 (0.73 - 1.09)	0.25	0.48
NKT CD4+	CD107a	6.20 (4.98, 9.00)	6.70 (2.68, 23.20)	0.77 (0.54 - 1.10)	0.15	0.33
NKT CD4+	CD25	7.40 (2.60, 14.90)	5.22 (3.73, 7.89)	1.37 (1.05 - 1.79)	0.02	0.088
NKT CD4+	CD69	11.80 (8.50, 13.00)	3.60 (1.80, 7.40)	1.24 (1.05 - 1.46)	0.012	0.076
NKT CD4+	GranzB	-2.40 (-7.80, -0.80)	-3.70 (-4.90, 1.00)	0.93 (0.78 - 1.12)	0.46	0.68
NKT CD4+	IFNγ	1.82 (0.42, 3.53)	0.74 (0.07, 1.86)	1.27 (0.97 - 1.67)	0.081	0.22
NKT CD4+	Ki67	-0.09 (-0.48, 0.28)	0.15 (0.02, 0.41)	0.86 (0.76 - 0.97)	0.015	0.08
NKT CD4+	PD1	1.20 (-0.10, 2.90)	0.50 (-0.90, 1.60)	1.01 (0.87 - 1.17)	0.93	0.95
NKT CD8+	CD107a	9.90 (7.20, 12.85)	1.87 (1.26, 3.50)	2.05 (1.31 - 3.21)	0.0018	0.032
NKT CD8+	CD25	7.17 (4.17, 10.76)	1.70 (1.13, 3.00)	1.59 (1.12 - 2.27)	0.0099	0.072
NKT CD8+	CD69	0.70 (-0.60, 14.80)	4.80 (1.40, 4.92)	1.16 (0.88 - 1.53)	0.28	0.51
NKT CD8+	GranzB	-7.40 (-10.30, -4.80)	-2.80 (-7.80, 0.10)	0.89 (0.63 - 1.26)	0.51	0.72
NKT CD8+	IL10	1.45 (0.66, 2.88)	0.24 (0.14, 0.43)	1.28 (0.94 - 1.76)	0.12	0.28
NKT CD8+	IL2	1.09 (0.31, 2.50)	0.18 (0.06, 0.30)	1.09 (0.79 - 1.51)	0.60	0.76
NKT CD8+	Ki67	0.04 (-0.45, 0.42)	-0.07 (-0.27, 0.00)	1.01 (0.91 - 1.13)	0.86	0.93
NKT CD8+	PD1	-0.28 (-0.40, 1.81)	0.82 (-0.20, 1.43)	0.95 (0.80 - 1.14)	0.58	0.74
Tconv CD4+	CD107a	0.49 (0.30, 0.53)	0.37 (-0.07, 0.44)	1.08 (0.99 - 1.17)	0.07	0.21
Tconv CD4+	CD25	7.40 (4.77, 10.97)	4.20 (2.10, 5.70)	1.28 (1.11 - 1.48)	0.00088	0.022
Tconv CD4+	CD69	2.10 (0.10, 8.30)	0.30 (-2.70, 2.60)	1.28 (1.14 - 1.42)	1.1e-05	0.0012
Tconv CD4+	GranzB	0.20 (0.04, 0.58)	0.30 (0.14, 0.37)	0.90 (0.81 - 1.01)	0.086	0.23
Tconv CD4+	PD1	1.90 (1.60, 2.90)	2.00 (1.40, 3.10)	1.00 (0.93 - 1.08)	0.92	0.95
Tconv CD8+	CD25	1.73 (0.89, 2.79)	0.26 (0.22, 0.65)	1.25 (1.06 - 1.46)	0.0064	0.069
Tconv CD8+	CD69	1.20 (-2.40, 3.80)	-1.80 (-3.20, 2.50)	1.13 (1.02 - 1.26)	0.018	0.081
Tconv CD8+	GranzB	1.33 (1.00, 2.00)	0.60 (0.40, 1.20)	0.95 (0.79 - 1.14)	0.56	0.74
Tconv CD8+	PD1	1.30 (0.91, 1.80)	1.50 (0.62, 2.20)	0.97 (0.88 - 1.06)	0.46	0.68
γδT	CD107a	2.28 (0.85, 4.50)	0.42 (0.29, 0.59)	1.03 (0.81 - 1.30)	0.84	0.93
γδT	CD25	2.84 (2.17, 4.70)	1.28 (0.40, 2.14)	1.39 (1.17 - 1.66)	0.00018	0.0082
γδT	CD69	4.10 (0.20, 17.10)	6.60 (-0.40, 8.30)	1.51 (1.21 - 1.88)	0.00023	0.0082
γδT	GranzB	2.90 (0.20, 4.50)	0.70 (-0.80, 1.60)	1.08 (0.99 - 1.19)	0.089	0.23
γδT	IFNγ	3.62 (1.93, 6.10)	0.64 (0.29, 1.18)	1.33 (0.99 - 1.79)	0.059	0.19
γδT	Ki67	0.18 (0.05, 0.32)	0.03 (-0.11, 0.12)	1.05 (0.99 - 1.11)	0.077	0.22
γδT	PD1	2.30 (1.68, 4.30)	2.80 (1.10, 3.40)	0.94 (0.85 - 1.04)	0.22	0.45

* Median (quartiles)

**Point estimate (95% Confidence interval)

Table S5. Frequency of cell subsets in TB-resisters and BCG-recipients in unstimulated conditions related to Fig 6

Cell Subset	Marker	Resister	BCG	Odds Ratio	p value	FDR p
cDC1	CD40	67.65 (63.53, 75.23)*	82.30 (71.90, 89.40)	0.80 (1.66 - 0.38)**	0.55	0.63
cDC1	CD80	6.98 (3.75, 9.59)	12.70 (5.48, 19.40)	0.63 (1.03 - 0.38)	0.066	0.12
cDC1	CD83	47.55 (30.75, 59.85)	39.60 (14.95, 44.35)	1.80 (3.36 - 0.96)	0.067	0.12
cDC1	IL10	1.47 (0.16, 3.16)	3.17 (1.90, 4.16)	0.72 (1.02 - 0.51)	0.061	0.12
cDC1	IL1β	1.39 (0.00, 3.09)	15.40 (7.62, 32.95)	0.17 (0.30 - 0.10)	3.8e-10	7.1e-09
cDC1	IL27	33.55 (16.62, 41.25)	20.60 (8.08, 35.80)	1.30 (2.37 - 0.72)	0.39	0.46
cDC1	IL8	7.51 (5.25, 12.45)	39.20 (25.95, 58.20)	0.17 (0.29 - 0.099)	9.8e-11	2.1e-09
cDC1	PDL1	66.50 (33.05, 69.75)	87.50 (80.75, 93.15)	0.32 (0.59 - 0.17)	0.00033	0.0013
cDC2	CD40	55.85 (44.60, 71.30)	77.90 (67.55, 89.85)	0.44 (0.79 - 0.25)	0.006	0.017
cDC2	CD80	1.62 (0.93, 2.04)	3.44 (1.81, 3.89)	0.75 (0.96 - 0.58)	0.025	0.055
cDC2	CD83	17.30 (12.17, 21.93)	26.60 (19.80, 43.95)	0.64 (1.10 - 0.37)	0.10	0.17
cDC2	IL1β	2.82 (2.47, 3.73)	9.46 (8.43, 21.40)	0.29 (0.57 - 0.15)	0.00025	0.001
cDC2	IL27	18.80 (11.55, 23.80)	19.10 (16.00, 35.85)	0.60 (1.04 - 0.34)	0.071	0.12
cDC2	IL8	12.70 (9.10, 18.65)	37.80 (31.70, 51.05)	0.20 (0.36 - 0.11)	1e-07	8.4e-07
cDC2	PDL1	66.35 (45.12, 73.17)	84.00 (79.30, 91.95)	0.29 (0.51 - 0.17)	1.2e-05	7e-05
cDC2	TNFα	0.22 (0.03, 0.33)	0.18 (0.00, 0.90)	0.87 (1.01 - 0.75)	0.064	0.12
Mono	CD40	35.65 (21.43, 43.95)	50.60 (44.35, 65.40)	0.56 (0.98 - 0.32)	0.042	0.089
Mono	CD80	6.19 (4.88, 7.25)	10.20 (6.51, 12.20)	0.70 (0.90 - 0.55)	0.0043	0.012
Mono	CD83	1.00 (0.80, 1.15)	1.99 (1.78, 4.54)	0.48 (0.91 - 0.25)	0.024	0.055
Mono	GMCSF	0.41 (0.24, 0.49)	0.14 (0.11, 0.17)	0.96 (1.13 - 0.82)	0.64	0.69
Mono	IL10	0.04 (0.03, 0.05)	0.30 (0.14, 0.80)	0.68 (0.96 - 0.48)	0.03	0.066
Mono	IL12p40	0.03 (0.02, 0.08)	0.20 (0.08, 0.35)	0.92 (0.98 - 0.86)	0.0095	0.025
Mono	IL1β	3.66 (1.92, 9.72)	66.40 (43.65, 78.15)	0.07 (0.14 - 0.04)	1e-15	1.3e-13
Mono	IL27	0.45 (0.24, 0.72)	0.50 (0.26, 1.06)	0.77 (0.96 - 0.62)	0.022	0.053
Mono	IL8	41.30 (27.00, 54.20)	86.60 (80.20, 88.40)	0.18 (0.32 - 0.11)	1.8e-09	2.6e-08
Mono	PDL1	39.45 (21.73, 58.38)	81.50 (67.25, 83.50)	0.23 (0.41 - 0.13)	6.5e-07	4.3e-06
Mono	TNFα	0.02 (0.01, 0.07)	0.04 (0.03, 0.69)	0.80 (0.92 - 0.69)	0.0023	0.007
pDC	CD40	31.85 (23.50, 35.12)	51.50 (39.85, 59.45)	0.39 (0.55 - 0.27)	1e-07	8.4e-07
pDC	CD80	0.18 (0.00, 1.08)	1.20 (0.56, 1.84)	0.83 (1.06 - 0.65)	0.13	0.20
pDC	CD83	20.05 (11.85, 24.80)	20.60 (15.90, 29.40)	0.78 (1.11 - 0.55)	0.17	0.25
pDC	IL27	10.15 (6.44, 13.25)	10.90 (7.19, 12.70)	0.85 (1.19 - 0.61)	0.34	0.43
pDC	IL8	0.86 (0.60, 1.58)	4.00 (3.65, 8.07)	0.41 (0.57 - 0.30)	3.3e-08	3.3e-07
pDC	PDL1	8.05 (5.20, 11.45)	22.20 (16.80, 30.35)	0.28 (0.56 - 0.14)	0.00034	0.0013
iNKT CD4+	CD107a	3.26 (2.25, 5.57)	4.49 (3.76, 5.02)	0.93 (1.25 - 0.69)	0.63	0.69
iNKT CD4+	CD25	11.80 (6.79, 15.40)	5.16 (3.98, 6.43)	1.74 (2.46 - 1.23)	0.0017	0.0054
iNKT CD4+	CD69	44.20 (32.70, 49.80)	45.70 (37.00, 59.30)	0.74 (1.12 - 0.49)	0.16	0.23
iNKT CD4+	GMCSF	0.53 (0.24, 0.80)	0.42 (0.13, 0.68)	1.03 (1.18 - 0.90)	0.64	0.69
iNKT CD4+	GranzB	37.70 (12.20, 45.60)	30.50 (20.70, 37.60)	1.07 (1.87 - 0.61)	0.82	0.83
iNKT CD4+	IFNγ	0.13 (0.10, 0.16)	0.00 (0.00, 0.26)	0.95 (1.14 - 0.79)	0.57	0.64
iNKT CD4+	IL10	0.69 (0.45, 0.96)	0.00 (0.00, 0.27)	1.30 (1.41 - 1.19)	1.7e-09	2.6e-08
iNKT CD4+	Ki67	15.50 (11.50, 22.20)	3.83 (3.56, 5.40)	3.03 (4.41 - 2.08)	8.3e-09	1.1e-07
iNKT CD4+	PD1	37.60 (32.70, 47.90)	35.10 (30.20, 46.30)	1.13 (1.60 - 0.81)	0.47	0.55
iNKT CD4+	TNFα	0.26 (0.13, 0.33)	0.31 (0.26, 0.73)	0.91 (1.01 - 0.83)	0.078	0.13
iNKT CD8+	CD107a	1.16 (0.77, 1.33)	2.20 (1.87, 2.38)	0.71 (0.87 - 0.57)	0.00099	0.0035
iNKT CD8+	CD25	0.54 (0.30, 0.65)	0.66 (0.49, 0.96)	0.88 (1.06 - 0.72)	0.17	0.25
iNKT CD8+	CD69	17.10 (12.80, 21.50)	33.70 (20.80, 41.20)	0.46 (0.74 - 0.29)	0.0013	0.0044
iNKT CD8+	GMCSF	0.02 (0.00, 0.08)	0.07 (0.06, 0.14)	0.96 (0.99 - 0.94)	0.004	0.012
iNKT CD8+	GranzB	92.70 (86.70, 95.50)	90.60 (83.50, 94.20)	1.31 (2.21 - 0.77)	0.32	0.40
iNKT CD8+	IFNγ	0.04 (0.03, 0.08)	0.06 (0.00, 0.17)	0.94 (1.03 - 0.86)	0.20	0.28
iNKT CD8+	IL10	0.20 (0.14, 0.35)	0.28 (0.14, 0.48)	0.94 (1.07 - 0.82)	0.31	0.40
iNKT CD8+	IL17	2.08 (1.01, 2.82)	0.20 (0.14, 0.39)	1.82 (2.17 - 1.54)	8.3e-12	2.7e-10
iNKT CD8+	IL2	0.03 (0.01, 0.08)	0.28 (0.17, 0.41)	0.85 (0.96 - 0.75)	0.008	0.022
iNKT CD8+	Ki67	3.51 (2.36, 4.75)	2.75 (1.34, 3.59)	1.16 (1.50 - 0.90)	0.26	0.34

**iNKT CD8+**	PD1	27.30 (11.10, 42.20)	34.20 (29.20, 50.70)	0.71 (1.16 - 0.43)	0.17	0.25
**iNKT CD8+**	TNFα	0.10 (0.04, 0.15)	0.16 (0.07, 0.24)	0.96 (1.00 - 0.93)	0.043	0.089
**MAIT MR1-**	CD107a	0.14 (0.10, 0.19)	0.29 (0.27, 0.45)	0.89 (0.98 - 0.81)	0.013	0.034
**MAIT MR1-**	CD25	2.98 (2.34, 3.83)	2.55 (0.57, 3.35)	1.25 (1.58 - 0.99)	0.062	0.12
**MAIT MR1-**	CD69	30.10 (25.10, 38.40)	58.10 (25.50, 58.90)	0.60 (1.09 - 0.33)	0.095	0.15
**MAIT MR1-**	GranzB	7.42 (3.17, 16.20)	32.10 (26.70, 88.00)	0.25 (0.54 - 0.11)	0.00044	0.0016
**MAIT MR1-**	IFNγ	0.03 (0.02, 0.03)	0.00 (0.00, 0.01)	0.98 (1.05 - 0.91)	0.56	0.63
**MAIT MR1-**	IL10	0.38 (0.25, 0.60)	0.16 (0.06, 0.24)	1.13 (1.18 - 1.07)	9.3e-07	5.8e-06
**MAIT MR1-**	IL2	0.02 (0.01, 0.03)	0.03 (0.01, 0.10)	0.98 (1.01 - 0.96)	0.18	0.26
**MAIT MR1-**	Ki67	0.45 (0.39, 0.62)	0.65 (0.46, 1.09)	0.77 (0.94 - 0.63)	0.0095	0.025
**MAIT MR1-**	PD1	10.60 (8.02, 16.10)	31.30 (23.60, 32.60)	0.43 (0.58 - 0.32)	3.9e-08	3.7e-07
**MAIT MR1-**	TNFα	0.04 (0.03, 0.05)	0.06 (0.04, 0.06)	0.99 (1.00 - 0.97)	0.062	0.12
**MAIT MR1+**	CD107a	0.26 (0.12, 0.33)	0.33 (0.16, 0.75)	0.85 (1.11 - 0.64)	0.22	0.31
**MAIT MR1+**	CD25	0.35 (0.29, 0.41)	0.63 (0.29, 1.40)	0.73 (1.04 - 0.51)	0.082	0.13
**MAIT MR1+**	CD69	72.90 (70.30, 74.80)	90.90 (81.30, 92.60)	0.34 (0.57 - 0.20)	6.1e-05	0.00032
**MAIT MR1+**	GranzB	0.15 (0.11, 0.29)	1.17 (0.60, 2.32)	0.72 (0.96 - 0.54)	0.025	0.055
**MAIT MR1+**	IFNγ	0.04 (0.01, 0.11)	0.05 (0.01, 0.07)	0.92 (1.15 - 0.74)	0.46	0.54
**MAIT MR1+**	IL10	0.47 (0.31, 0.62)	0.16 (0.11, 0.26)	0.96 (1.33 - 0.69)	0.79	0.81
**MAIT MR1+**	Ki67	0.30 (0.23, 0.37)	0.40 (0.32, 0.48)	0.97 (1.04 - 0.91)	0.39	0.46
**MAIT MR1+**	PD1	1.10 (0.58, 1.97)	2.04 (1.60, 2.57)	0.72 (0.89 - 0.58)	0.0032	0.0095
**NK**	CD107a	2.43 (2.26, 3.43)	2.06 (1.68, 3.98)	0.95 (1.58 - 0.57)	0.84	0.84
**NK**	CD25	1.08 (0.80, 2.18)	2.85 (1.75, 6.38)	0.73 (1.08 - 0.49)	0.11	0.18
**NK**	CD69	41.10 (38.50, 47.20)	65.10 (53.50, 76.40)	0.32 (0.51 - 0.20)	2e-06	1.2e-05
**NK**	GMCSF	0.01 (0.01, 0.02)	0.05 (0.03, 0.08)	0.97 (1.00 - 0.94)	0.045	0.09
**NK**	GranzB	64.10 (52.90, 74.90)	79.10 (69.80, 86.60)	0.62 (0.99 - 0.39)	0.043	0.089
**NK**	IFNγ	0.16 (0.10, 0.20)	0.05 (0.03, 0.07)	0.96 (1.16 - 0.79)	0.67	0.71
**NK**	IL10	0.14 (0.12, 0.21)	0.07 (0.06, 0.12)	1.05 (1.09 - 1.01)	0.016	0.04
**NK**	IL17	0.01 (0.01, 0.01)	0.01 (0.00, 0.01)	1.00 1.01 - 1.00)	0.28	0.37
**NK**	IL2	0.00 (0.00, 0.01)	0.02 (0.01, 0.03)	0.98 (1.01 - 0.96)	0.14	0.21
**NK**	Ki67	4.28 (3.79, 5.67)	4.22 (3.69, 5.29)	0.84 (1.11 - 0.64)	0.22	0.31
**NK**	PD1	1.22 (1.12, 2.05)	2.31 (1.66, 3.05)	0.79 (1.03 - 0.61)	0.078	0.13
**NK**	TNFα	0.09 (0.08, 0.13)	0.07 (0.05, 0.16)	0.99 (1.02 - 0.96)	0.56	0.63
**NKT CD4+**	CD107a	19.30 (9.48, 23.60)	4.80 (2.37, 17.20)	1.71 (3.04 - 0.96)	0.071	0.12
**NKT CD4+**	CD25	10.40 (8.13, 16.10)	4.57 (4.20, 6.81)	1.62 (2.45 - 1.07)	0.024	0.055
**NKT CD4+**	CD69	39.80 (27.00, 53.60)	53.30 (26.80, 66.00)	0.75 (1.26 - 0.45)	0.28	0.37
**NKT CD4+**	GranzB	18.50 (11.40, 32.80)	41.60 (29.20, 61.20)	0.35 (0.62 - 0.20)	0.00029	0.0012
**NKT CD4+**	Ki67	3.16 (1.82, 3.78)	2.48 (1.58, 2.92)	1.23 (1.74 - 0.87)	0.25	0.34
**NKT CD4+**	PD1	24.40 (14.70, 36.70)	22.30 (17.40, 25.00)	1.47 (2.30 - 0.94)	0.095	0.15
**NKT CD8+**	CD107a	6.48 (3.59, 11.40)	2.19 (1.09, 4.35)	1.59 (2.65 - 0.95)	0.079	0.13
**NKT CD8+**	CD25	4.82 (1.58, 6.65)	0.90 (0.58, 3.36)	1.55 (2.47 - 0.97)	0.069	0.12
**NKT CD8+**	CD69	24.00 (16.90, 34.70)	24.50 (13.70, 37.80)	0.91 (1.40 - 0.59)	0.66	0.70
**NKT CD8+**	GranzB	55.40 (37.20, 70.60)	87.40 (82.40, 91.10)	0.25 (0.42 - 0.15)	1.3e-07	1e-06
**NKT CD8+**	IL10	3.92 (1.48, 7.78)	0.23 (0.10, 0.54)	1.87 (3.14 - 1.11)	0.019	0.047
**NKT CD8+**	IL2	1.17 (0.74, 1.62)	0.22 (0.12, 0.40)	1.17 (1.77 - 0.77)	0.46	0.54
**NKT CD8+**	Ki67	1.94 (1.17, 3.31)	1.33 (0.91, 1.81)	1.18 (1.55 - 0.90)	0.23	0.31
**NKT CD8+**	PD1	10.70 (4.74, 14.20)	9.10 (7.15, 12.20)	1.09 (1.62 - 0.73)	0.68	0.71
**Tconv CD4+**	CD107a	0.26 (0.18, 0.35)	0.60 (0.48, 0.83)	0.83 (0.89 - 0.77)	2.4e-07	1.6e-06
**Tconv CD4+**	CD25	10.50 (8.25, 11.30)	12.90 (10.70, 13.30)	0.78 (0.91 - 0.66)	0.0017	0.0054
**Tconv CD4+**	CD69	36.60 (29.40, 40.20)	52.60 (50.00, 62.70)	0.45 (0.70 - 0.30)	3e-04	0.0012
**Tconv CD4+**	GMCSF	0.00 (0.00, 0.00)	0.02 (0.00, 0.03)	0.99 (1.00 - 0.99)	0.00022	0.00097
**Tconv CD4+**	GranzB	0.92 (0.18, 1.66)	4.02 (2.88, 5.92)	0.52 (0.73 - 0.37)	0.00017	0.00083
**Tconv CD4+**	IFNγ	0.02 (0.02, 0.03)	0.01 (0.01, 0.01)	1.00 (1.01 - 0.99)	0.68	0.71
**Tconv CD4+**	IL10	0.11 (0.07, 0.13)	0.03 (0.02, 0.03)	1.04 (1.05 - 1.03)	1.6e-12	1e-10
**Tconv CD4+**	IL17	0.00 (0.00, 0.00)	0.00 (0.00, 0.00)	1.00 1.00 - 1.00)	0.18	0.26
**Tconv CD4+**	IL2	0.00 (0.00, 0.00)	0.00 (0.00, 0.01)	1.00 (1.00 - 1.00)	0.061	0.12
**Tconv CD4+**	Ki67	0.89 (0.63, 0.98)	0.76 (0.68, 0.85)	0.98 (1.09 - 0.89)	0.72	0.74
**Tconv CD4+**	PD1	13.00 (7.41, 14.90)	21.00 (18.30, 25.00)	0.51 (0.64 - 0.40)	1e-08	1.2e-07

**Tconv CD4+**	TNFα	0.00 (0.00, 0.00)	0.01 (0.01, 0.01)	1.00 (1.00 - 0.99)	0.0021	0.0066
**Tconv CD8+**	CD107a	0.26 (0.17, 0.33)	0.52 (0.39, 0.68)	0.87 (0.93 - 0.82)	1.6e-05	8.5e-05
**Tconv CD8+**	CD25	0.70 (0.50, 1.16)	0.80 (0.45, 0.99)	1.09 (1.29 - 0.92)	0.34	0.42
**Tconv CD8+**	CD69	24.10 (14.40, 34.20)	44.60 (33.10, 57.70)	0.42 (0.66 - 0.26)	0.00024	0.001
**Tconv CD8+**	GranzB	9.16 (6.08, 17.80)	37.50 (34.60, 61.70)	0.25 (0.42 - 0.15)	1.7e-07	1.2e-06
**Tconv CD8+**	IFNγ	0.02 (0.01, 0.03)	0.01 (0.01, 0.01)	1.00 (1.01 - 0.99)	0.84	0.84
**Tconv CD8+**	IL10	0.11 (0.08, 0.17)	0.03 (0.03, 0.03)	1.05 (1.07 - 1.03)	1.5e-08	1.6e-07
**Tconv CD8+**	IL2	0.01 (0.01, 0.02)	0.01 (0.01, 0.02)	1.00 (1.00 - 0.99)	0.32	0.40
**Tconv CD8+**	Ki67	0.42 (0.31, 0.51)	0.52 (0.49, 0.73)	0.89 (1.00 - 0.79)	0.043	0.089
**Tconv CD8+**	PD1	8.88 (6.44, 11.80)	18.50 (15.60, 39.90)	0.44 (0.67 - 0.28)	0.00018	0.00083
**Tconv CD8+**	TNFα	0.00 (0.00, 0.01)	0.01 (0.01, 0.02)	0.99 (1.00 - 0.99)	8.4e-05	0.00043
**γδT**	CD107a	0.45 (0.26, 0.58)	0.42 (0.34, 0.53)	0.80 (1.32 - 0.48)	0.37	0.46
**γδT**	CD25	0.63 (0.49, 0.97)	0.30 (0.22, 0.57)	0.87 (1.48 - 0.51)	0.61	0.68
**γδT**	CD69	30.80 (23.70, 33.60)	53.20 (48.60, 63.90)	0.31 (0.43 - 0.22)	5e-12	2.2e-10
**γδT**	GranzB	32.60 (29.50, 48.80)	39.80 (35.10, 50.90)	0.69 (1.04 - 0.46)	0.08	0.13
**γδT**	IFNγ	0.06 (0.05, 0.08)	0.04 (0.02, 0.06)	0.81 (1.22 - 0.54)	0.31	0.40
**γδT**	IL10	0.49 (0.29, 0.57)	0.10 (0.08, 0.16)	1.17 (1.22 - 1.11)	2.7e-11	7e-10
**γδT**	IL17	0.53 (0.34, 0.75)	0.10 (0.03, 0.14)	1.30 (1.53 - 1.10)	0.002	0.0062
**γδT**	Ki67	1.15 (0.81, 1.29)	0.77 (0.59, 1.46)	0.95 (1.15 - 0.79)	0.61	0.67
**γδT**	PD1	18.10 (13.20, 24.70)	14.40 (12.30, 16.50)	1.17 (1.65 - 0.82)	0.39	0.46
**γδT**	TNFα	0.02 (0.00, 0.03)	0.03 (0.01, 0.10)	0.97 (0.99 - 0.94)	0.02	0.047

* Median (quartiles)

**Point estimate (95% Confidence interval)

Table S6. Frequency of cell subsets in TB-resisters and BCG-recipients in Mtb-stimulated conditions related to Fig 6

Cell Subset	Marker	Resister	BCG	Odds Ratio	p value	FDR p
cDC1	CD40	94.10 (91.72, 95.65)*	89.20 (86.25, 93.30)	1.40 (2.30 - 0.86)**	0.18	0.31
cDC1	CD80	10.18 (8.23, 12.60)	13.90 (9.42, 16.30)	0.78 (1.04 - 0.58)	0.092	0.18
cDC1	CD83	57.35 (49.83, 61.20)	43.60 (36.90, 49.15)	1.69 (2.18 - 1.31)	6.5e-05	0.00042
cDC1	IL10	0.58 (0.41, 0.97)	1.65 (1.05, 3.52)	0.63 (0.86 - 0.46)	0.003	0.0089
cDC1	IL1β	40.05 (25.82, 52.20)	57.30 (51.45, 79.50)	0.39 (0.62 - 0.25)	6.7e-05	0.00042
cDC1	IL27	72.50 (66.68, 77.95)	49.60 (44.10, 59.65)	2.29 (3.33 - 1.57)	1.5e-05	0.00011
cDC1	IL8	49.15 (40.23, 55.58)	70.90 (67.45, 82.25)	0.33 (0.50 - 0.22)	1.2e-07	1.7e-06
cDC1	PDL1	93.65 (92.08, 95.97)	98.60 (96.35, 99.40)	0.60 (0.92 - 0.39)	0.019	0.048
cDC1	TNFα	1.25 (0.66, 2.04)	1.28 (0.66, 2.38)	0.99 (1.27 - 0.77)	0.93	0.99
cDC2	CD40	92.80 (87.98, 94.50)	89.40 (87.45, 92.30)	1.17 (1.71 - 0.80)	0.41	0.60
cDC2	CD80	1.21 (0.62, 1.39)	5.81 (3.33, 7.83)	0.44 (0.57 - 0.34)	1.1e-09	2.8e-08
cDC2	CD83	29.55 (24.55, 34.72)	26.70 (24.90, 30.80)	1.09 (1.47 - 0.81)	0.58	0.76
cDC2	GMCSF	0.57 (0.33, 0.76)	1.09 (0.54, 1.82)	0.73 (0.90 - 0.60)	0.0024	0.0076
cDC2	IL10	1.75 (0.84, 2.18)	2.25 (0.46, 3.08)	0.77 (1.19 - 0.50)	0.24	0.38
cDC2	IL12p40	0.17 (0.11, 0.55)	0.44 (0.17, 0.98)	0.89 (1.00 - 0.79)	0.055	0.12
cDC2	IL1β	72.55 (67.88, 76.55)	61.50 (54.80, 74.95)	1.40 (2.01 - 0.98)	0.064	0.13
cDC2	IL27	78.40 (72.75, 81.92)	56.10 (51.15, 59.70)	2.90 (3.84 - 2.18)	1.6e-13	9.6e-12
cDC2	IL8	82.25 (79.40, 83.85)	86.10 (81.75, 92.00)	0.75 (0.96 - 0.59)	0.024	0.058
cDC2	PDL1	98.65 (98.12, 99.27)	98.50 (96.00, 98.80)	1.60 (2.09 - 1.23)	0.00046	0.002
cDC2	TNFα	2.34 (2.08, 3.47)	2.11 (1.09, 3.88)	1.09 (1.44 - 0.83)	0.54	0.73
Mono	CD40	55.15 (42.12, 67.25)	49.00 (43.15, 66.85)	1.03 (1.62 - 0.66)	0.89	0.96
Mono	CD80	0.79 (0.57, 1.27)	5.89 (2.65, 6.96)	0.44 (0.58 - 0.33)	1.4e-08	3e-07
Mono	CD83	14.25 (7.69, 18.20)	10.80 (5.71, 17.20)	0.89 (1.42 - 0.56)	0.64	0.82
Mono	GMCSF	0.12 (0.09, 0.19)	0.57 (0.26, 1.17)	0.70 (0.86 - 0.57)	0.00061	0.0025
Mono	IL10	3.42 (1.56, 5.16)	1.22 (0.94, 2.41)	1.26 (1.79 - 0.89)	0.20	0.33
Mono	IL1β	97.95 (97.05, 98.75)	98.00 (94.80, 98.20)	1.64 (2.55 - 1.06)	0.026	0.061
Mono	IL27	6.24 (4.41, 7.83)	2.08 (1.41, 3.03)	1.72 (2.41 - 1.23)	0.0015	0.0053
Mono	IL8	95.55 (93.52, 96.10)	97.10 (95.45, 97.80)	1.09 (1.58 - 0.75)	0.64	0.82
Mono	PDL1	99.40 (99.05, 99.50)	97.00 (94.50, 98.75)	2.36 (3.71 - 1.51)	0.00018	0.0011
Mono	TNFα	0.65 (0.43, 1.30)	0.62 (0.22, 0.90)	1.08 (1.28 - 0.91)	0.39	0.59
pDC	CD40	67.45 (65.43, 75.05)	73.10 (65.55, 76.15)	0.98 (1.25 - 0.76)	0.86	0.95
pDC	CD80	2.25 (1.42, 2.98)	2.79 (1.77, 3.27)	0.94 (1.25 - 0.71)	0.69	0.85
pDC	CD83	25.90 (20.15, 29.85)	28.20 (22.20, 34.45)	0.95 (1.36 - 0.66)	0.78	0.91
pDC	IL10	0.04 (0.00, 0.24)	1.14 (0.78, 2.32)	0.60 (0.73 - 0.49)	5.9e-07	5.2e-06
pDC	IL1β	11.70 (10.01, 26.68)	14.70 (9.66, 21.75)	1.09 (1.86 - 0.64)	0.75	0.88
pDC	IL27	30.25 (27.02, 40.03)	24.00 (16.85, 27.90)	1.72 (2.42 - 1.22)	0.002	0.0066
pDC	IL8	8.47 (6.64, 12.25)	15.30 (12.80, 20.60)	0.58 0.74 - 0.45)	1.9e-05	0.00014
pDC	PDL1	73.40 (67.67, 82.15)	72.40 (59.80, 78.25)	1.07 (1.76 - 0.65)	0.80	0.92
pDC	TNFα	1.99 (1.31, 2.65)	0.49 (0.00, 1.00)	1.61 (1.94 - 1.34)	3.2e-07	3.1e-06
iNKT CD4+	CD107a	7.17 (6.61, 10.10)	6.07 (3.40, 7.14)	1.43 (2.00 - 1.02)	0.039	0.089
iNKT CD4+	CD25	24.40 (19.10, 32.60)	8.08 (5.74, 9.43)	3.06 (4.69 - 2.00)	2.6e-07	3.1e-06
iNKT CD4+	CD69	60.80 (45.60, 66.10)	43.70 (32.90, 51.20)	1.84 (2.72 - 1.25)	0.0022	0.0071
iNKT CD4+	GMCSF	1.57 (1.27, 3.08)	0.22 (0.18, 0.66)	1.65 (2.23 - 1.22)	0.0011	0.0042
iNKT CD4+	GranzB	34.50 (12.50, 43.40)	24.20 (19.30, 40.20)	0.96 (1.66 - 0.56)	0.89	0.96
iNKT CD4+	IFNγ	2.25 (1.93, 3.28)	0.42 (0.13, 0.71)	1.79 (2.67 - 1.20)	0.0047	0.013
iNKT CD4+	IL10	1.82 (1.07, 2.41)	0.19 (0.11, 0.26)	1.72 (1.99 - 1.49)	1.8e-13	9.6e-12
iNKT CD4+	IL2	0.38 (0.34, 1.82)	0.20 (0.16, 0.41)	1.22 (1.45 - 1.03)	0.023	0.057
iNKT CD4+	Ki67	10.80 (8.43, 15.90)	3.86 (3.00, 4.92)	2.43 (3.35 - 1.76)	7.4e-08	1.1e-06
iNKT CD4+	PD1	43.70 (35.80, 54.30)	31.90 (27.10, 43.90)	1.48 (2.18 - 1.01)	0.046	0.10
iNKT CD4+	TNFα	0.47 (0.35, 0.82)	0.58 (0.14, 0.88)	1.06 (1.22 - 0.92)	0.43	0.63
iNKT CD8+	CD107a	1.36 (0.79, 2.31)	3.13 (2.47, 3.73)	0.67 (0.87 - 0.52)	0.0027	0.0081
iNKT CD8+	CD25	2.31 (1.02, 3.08)	1.98 (1.55, 2.65)	1.07 (1.44 - 0.80)	0.63	0.82
iNKT CD8+	CD69	33.10 (24.70, 41.90)	34.80 (24.90, 41.70)	0.98 (1.40 - 0.68)	0.90	0.96

**iNKT CD8+**	GranzB	91.50 (81.40, 94.20)	85.60 (77.40, 92.60)	1.30 (2.19 - 0.77)	0.32	0.49
**iNKT CD8+**	IFNγ	0.52 (0.29, 1.74)	0.20 (0.19, 0.44)	1.32 (1.74 - 1.00)	0.052	0.11
**iNKT CD8+**	IL10	0.23 (0.17, 0.57)	0.21 (0.04, 0.80)	1.00 (1.32 - 0.75)	0.98	0.99
**iNKT CD8+**	IL17	1.79 (1.01, 2.65)	0.25 (0.19, 0.38)	1.55 (1.96 - 1.23)	0.00024	0.0013
**iNKT CD8+**	Ki67	3.17 (2.30, 4.56)	1.62 (1.35, 2.91)	1.21 (1.55 - 0.94)	0.15	0.27
**iNKT CD8+**	PD1	26.50 (17.70, 44.10)	30.30 (24.80, 37.90)	0.99 (1.63 - 0.60)	0.97	0.99
**MAIT MR1-**	CD25	4.35 (2.75, 5.31)	3.69 (1.22, 5.11)	1.19 (1.54 - 0.92)	0.18	0.31
**MAIT MR1-**	CD69	36.00 (27.90, 43.20)	54.90 (27.30, 61.50)	0.71 (1.23 - 0.42)	0.22	0.36
**MAIT MR1-**	GranzB	6.91 (3.00, 17.10)	36.60 (27.70, 87.40)	0.24 (0.52 - 0.11)	0.00031	0.0014
**MAIT MR1-**	PD1	12.10 (9.00, 21.10)	31.70 (25.50, 34.70)	0.47 (0.65 - 0.34)	4e-06	3.3e-05
**MAIT MR1+**	CD25	2.73 (2.27, 3.58)	3.48 (1.81, 8.13)	0.74 (1.13 - 0.49)	0.16	0.30
**MAIT MR1+**	CD69	85.50 (83.30, 87.60)	90.80 (84.20, 94.80)	0.68 (0.97 - 0.48)	0.035	0.082
**MAIT MR1+**	GranzB	0.61 (0.45, 0.82)	3.10 (0.92, 4.69)	0.60 (0.81 - 0.44)	0.001	0.0041
**MAIT MR1+**	IFNγ	0.84 (0.74, 2.25)	0.68 (0.43, 2.17)	1.05 (1.41 - 0.78)	0.75	0.88
**MAIT MR1+**	IL10	0.53 (0.42, 0.85)	0.27 (0.13, 0.52)	0.90 (1.56 - 0.52)	0.71	0.86
**MAIT MR1+**	PD1	1.43 (0.66, 2.18)	3.09 (2.54, 4.54)	0.73 (0.95 - 0.55)	0.022	0.055
**NK**	CD107a	5.65 (4.18, 9.21)	6.26 (3.61, 10.60)	0.81 (1.41 - 0.46)	0.46	0.64
**NK**	CD25	9.72 (8.38, 15.90)	8.46 (4.58, 12.70)	1.24 (1.88 - 0.82)	0.30	0.47
**NK**	CD69	80.90 (76.60, 88.90)	78.10 (68.30, 84.00)	1.29 (1.85 - 0.90)	0.17	0.31
**NK**	GranzB	73.40 (69.60, 86.60)	76.50 (69.20, 83.70)	1.11 (1.81 - 0.68)	0.67	0.83
**NK**	IFNγ	3.91 (2.25, 5.71)	1.42 (0.52, 3.23)	1.62 (2.39 - 1.10)	0.014	0.037
**NK**	Ki67	4.04 (3.70, 5.13)	3.59 (3.46, 4.56)	0.91 (1.16 - 0.72)	0.45	0.64
**NK**	PD1	4.17 (3.18, 7.05)	4.69 (3.33, 6.23)	1.00 (1.29 - 0.77)	0.99	0.99
**NKT CD4+**	CD107a	23.80 (13.20, 31.10)	17.60 (5.05, 41.20)	1.13 (1.95 - 0.65)	0.67	0.83
**NKT CD4+**	CD25	18.20 (15.80, 29.90)	13.40 (8.19, 15.60)	1.81 (2.61 - 1.26)	0.0013	0.0049
**NKT CD4+**	CD69	56.80 (47.40, 70.40)	56.20 (35.40, 69.70)	1.19 (1.87 - 0.76)	0.45	0.64
**NKT CD4+**	GranzB	26.40 (19.40, 33.90)	37.90 (27.50, 56.20)	0.49 (0.79 - 0.30)	0.0031	0.0089
**NKT CD4+**	IFNγ	1.93 (1.26, 3.79)	1.10 (0.59, 2.96)	1.35 (1.83 - 0.99)	0.058	0.12
**NKT CD4+**	Ki67	2.46 (2.10, 3.27)	2.58 (1.79, 3.26)	1.06 (1.31 - 0.86)	0.57	0.76
**NKT CD4+**	PD1	20.10 (16.40, 35.90)	23.70 (17.40, 26.00)	1.54 (2.42 - 0.99)	0.057	0.12
**NKT CD8+**	CD107a	6.02 (2.82, 7.36)	3.90 (1.78, 7.85)	1.01 (1.68 - 0.61)	0.97	0.99
**NKT CD8+**	CD25	4.03 (2.81, 12.90)	2.46 (1.71, 6.36)	1.38 (2.25 - 0.84)	0.20	0.34
**NKT CD8+**	CD69	42.40 (22.50, 57.80)	31.30 (18.60, 38.20)	1.40 (2.20 - 0.89)	0.14	0.27
**NKT CD8+**	GranzB	67.20 (51.50, 70.20)	85.00 (74.60, 89.00)	0.41 (0.67 - 0.26)	0.00032	0.0014
**NKT CD8+**	IL10	2.97 (1.57, 7.13)	0.43 (0.28, 0.92)	1.54 (2.61 - 0.91)	0.11	0.22
**NKT CD8+**	IL2	0.71 (0.41, 1.27)	0.41 (0.24, 0.65)	1.00 (1.62 - 0.61)	0.99	0.99
**NKT CD8+**	Ki67	1.53 (1.22, 2.71)	1.27 (0.88, 2.03)	1.09 (1.33 - 0.89)	0.41	0.60
**NKT CD8+**	PD1	12.00 (5.26, 19.60)	9.92 (7.83, 12.00)	1.05 (1.57 - 0.70)	0.82	0.93
**Tconv CD4+**	CD107a	0.43 (0.32, 0.53)	0.96 (0.84, 1.18)	0.81 (0.87 - 0.76)	2.7e-10	9.5e-09
**Tconv CD4+**	CD25	15.90 (13.60, 17.90)	15.70 (14.60, 17.60)	0.88 (1.06 - 0.73)	0.18	0.31
**Tconv CD4+**	CD69	40.40 (32.40, 45.20)	55.00 (47.90, 62.40)	0.58 (0.84 - 0.40)	0.0039	0.011
**Tconv CD4+**	GranzB	0.78 (0.29, 1.42)	4.34 (3.20, 6.08)	0.51 (0.74 - 0.36)	0.00026	0.0013
**Tconv CD4+**	PD1	15.10 (8.18, 16.20)	22.70 (20.60, 26.40)	0.52 (0.66 - 0.42)	2e-08	3.6e-07
**Tconv CD8+**	CD25	1.19 (0.71, 2.27)	1.18 (0.72, 1.58)	1.06 (1.27 - 0.88)	0.52	0.72
**Tconv CD8+**	CD69	24.70 (20.40, 31.90)	43.50 (36.20, 54.50)	0.48 (0.71 - 0.32)	0.00027	0.0013
**Tconv CD8+**	GranzB	9.43 (7.24, 18.70)	37.60 (35.00, 62.60)	0.25 (0.42 - 0.15)	2.9e-07	3.1e-06
**Tconv CD8+**	PD1	10.80 (6.77, 12.00)	20.60 (16.80, 40.90)	0.44 (0.68 - 0.28)	0.00022	0.0013
**γδT**	CD107a	1.19 (1.01, 1.60)	0.89 (0.62, 0.95)	0.91 (1.51 - 0.55)	0.71	0.86
**γδT**	CD25	2.47 (1.97, 3.80)	1.74 (1.44, 3.26)	0.96 (1.49 - 0.61)	0.84	0.95
**γδT**	CD69	53.00 (46.60, 54.80)	58.60 (54.70, 65.80)	0.68 (0.87 - 0.53)	0.0019	0.0065
**γδT**	GranzB	36.40 (33.40, 46.10)	40.50 (36.60, 49.50)	0.75 (1.10 - 0.51)	0.14	0.27
**γδT**	IFNγ	1.12 (0.68, 1.77)	0.87 (0.34, 1.31)	1.02 (1.53 - 0.68)	0.94	0.99
**γδT**	Ki67	1.21 (1.01, 1.41)	0.88 (0.71, 1.34)	0.99 (1.16 - 0.84)	0.89	0.96
**γδT**	PD1	22.10 (15.60, 28.20)	17.70 (15.10, 18.40)	1.21 (1.66 - 0.88)	0.24	0.38

* Median (quartiles)
**Point estimate (95% Confidence interval)

Table S7. Frequency of cell subsets in TB-resisters and BCG-recipients in Mtb-stimulated conditions after background subtraction related to Fig 6

Cell Subset	Marker	Resister	BCG	Odds Ratio	p value	FDR p
cDC1	CD40	23.15 (17.30, 28.95)*	10.40 (3.75, 15.95)	1.80 (2.57 - 1.26)**	0.0013	0.0055
cDC1	CD80	1.51 (0.41, 9.40)	1.10 (-5.20, 4.54)	0.81 (1.12 - 0.58)	0.20	0.34
cDC1	CD83	11.00 (0.13, 19.07)	12.10 (1.50, 23.40)	1.61 (2.10 - 1.24)	4e-04	0.0023
cDC1	IL10	-0.82 (-2.26, -0.09)	-0.49 (-2.09, 0.18)	0.78 (0.97 - 0.64)	0.022	0.055
cDC1	IL1β	39.32 (24.41, 51.62)	40.60 (34.80, 63.92)	0.34 (0.71 - 0.17)	0.0035	0.013
cDC1	IL27	38.75 (31.00, 56.30)	29.00 (24.70, 39.35)	2.22 (3.14 - 1.57)	7.3e-06	9.8e-05
cDC1	IL8	40.20 (30.92, 46.23)	27.20 (23.45, 51.20)	0.61 (1.14 - 0.33)	0.12	0.23
cDC1	PDL1	29.00 (26.00, 49.30)	12.50 (6.05, 17.45)	0.74 (1.24 - 0.44)	0.25	0.38
cDC1	TNFα	-0.66 (-2.19, 0.75)	0.49 (0.20, 1.54)	1.01 (1.34 - 0.76)	0.95	0.97
cDC2	CD40	34.55 (21.75, 44.57)	13.60 (5.70, 23.80)	1.26 (1.91 - 0.83)	0.28	0.41
cDC2	CD80	-0.45 (-1.23, 0.08)	2.37 (0.62, 4.53)	0.48 (0.63 - 0.37)	1.3e-07	4.5e-06
cDC2	CD83	13.41 (8.05, 17.27)	-3.30 (-8.45, 6.50)	1.46 (1.86 - 1.14)	0.0025	0.0099
cDC2	GMCSF	0.52 (0.25, 0.70)	0.40 (0.30, 1.40)	0.80 (0.98 - 0.65)	0.035	0.079
cDC2	IL10	1.32 (0.84, 1.89)	0.52 (0.38, 2.18)	1.11 (1.37 - 0.90)	0.31	0.44
cDC2	IL12p40	0.12 (0.10, 0.55)	0.34 (0.13, 0.98)	0.87 (0.98 - 0.77)	0.021	0.054
cDC2	IL1β	68.48 (65.36, 72.32)	39.96 (33.16, 51.89)	2.24 (3.15 - 1.59)	3.5e-06	6.2e-05
cDC2	IL27	58.97 (57.15, 60.70)	27.60 (22.15, 33.65)	3.52 (4.54 - 2.72)	6e-22	6.4e-20
cDC2	IL8	69.90 (63.95, 74.21)	48.50 (35.60, 55.35)	1.05 (1.50 - 0.74)	0.77	0.84
cDC2	PDL1	31.20 (25.75, 51.92)	15.00 (6.85, 17.50)	1.81 (2.60 - 1.27)	0.0011	0.005
cDC2	TNFα	2.29 (1.91, 3.14)	0.97 (0.54, 3.40)	1.07 (1.45 - 0.79)	0.65	0.76
Mono	CD40	20.85 (5.25, 28.85)	2.30 (-5.30, 6.00)	1.48 (2.20 - 0.99)	0.055	0.11
Mono	CD80	-4.96 (-5.82, -4.02)	-4.68 (-7.37, -1.13)	0.52 (0.71 - 0.38)	3.7e-05	0.00033
Mono	CD83	13.30 (6.93, 17.19)	3.98 (3.04, 9.22)	1.46 (2.11 - 1.01)	0.042	0.089
Mono	GMCSF	-0.24 (-0.33, -0.06)	0.50 (0.13, 1.02)	0.75 (0.82 - 0.69)	2e-11	1e-09
Mono	IL10	3.38 (1.51, 5.12)	0.82 (0.57, 1.27)	1.62 (2.14 - 1.22)	0.00077	0.0036
Mono	IL1β	93.17 (88.75, 95.88)	28.50 (18.05, 55.15)	0.99 (2.46 - 0.40)	0.99	0.99
Mono	IL27	5.53 (3.63, 7.58)	1.05 (0.88, 2.40)	1.77 (2.59 - 1.22)	0.003	0.011
Mono	IL8	52.85 (42.95, 66.25)	8.30 (4.90, 15.70)	1.23 (2.29 - 0.66)	0.51	0.63
Mono	PDL1	60.05 (41.17, 77.50)	16.50 (9.60, 31.30)	2.26 (4.42 - 1.16)	0.017	0.046
Mono	TNFα	0.63 (0.41, 1.26)	0.16 (-0.11, 0.44)	1.19 (1.45 - 0.98)	0.077	0.14
pDC	CD40	35.05 (31.95, 50.28)	18.60 (15.40, 26.85)	0.86 (1.25 - 0.59)	0.42	0.57
pDC	CD80	2.00 (0.42, 2.96)	1.58 (0.43, 2.55)	0.94 (1.26 - 0.70)	0.67	0.76
pDC	CD83	6.85 (-5.52, 16.80)	8.10 (0.25, 12.00)	0.93 (1.33 - 0.64)	0.68	0.76
pDC	IL10	0.00 (-0.39, 0.00)	-0.26 (-0.52, 0.21)	0.78 (0.90 - 0.69)	0.00043	0.0023
pDC	IL1β	11.70 (9.87, 26.68)	14.03 (7.63, 21.55)	0.79 (1.54 - 0.41)	0.50	0.63
pDC	IL27	21.01 (14.35, 32.39)	14.12 (5.83, 17.01)	1.70 (2.40 - 1.21)	0.0025	0.0099
pDC	IL8	7.87 (5.49, 11.15)	11.30 (6.51, 14.53)	0.51 (0.72 - 0.36)	0.00015	0.0011
pDC	PDL1	65.23 (56.88, 71.34)	35.20 (25.75, 54.15)	1.96 (3.33 - 1.15)	0.013	0.038
pDC	TNFα	1.18 (0.49, 1.79)	0.49 (-0.09, 0.95)	1.61 (1.95 - 1.32)	1.9e-06	4.2e-05
iNKT CD4+	CD107a	4.21 (3.57, 4.56)	0.28 (-0.28, 2.71)	1.47 (1.80 - 1.20)	2e-04	0.0013
iNKT CD4+	CD25	14.57 (3.80, 22.10)	1.87 (0.45, 4.94)	2.56 (4.09 - 1.60)	9.1e-05	0.00075
iNKT CD4+	CD69	14.30 (10.70, 18.90)	-5.00 (-5.90, 0.00)	2.25 (3.06 - 1.65)	2.7e-07	7.2e-06
iNKT CD4+	GMCSF	1.06 (0.75, 2.93)	0.00 (-0.21, 0.20)	1.65 (2.13 - 1.28)	0.00012	0.00088
iNKT CD4+	GranzB	-1.26 (-8.60, 0.10)	2.40 (-3.60, 3.70)	0.79 (0.98 - 0.63)	0.034	0.079
iNKT CD4+	IFNγ	1.97 (1.56, 3.03)	0.33 (0.00, 0.55)	1.76 (2.64 - 1.18)	0.006	0.022
iNKT CD4+	IL10	0.82 (0.63, 1.61)	0.10 (0.00, 0.18)	1.35 (1.58 - 1.15)	0.00025	0.0016
iNKT CD4+	IL2	0.38 (0.33, 1.32)	0.00 (-0.08, 0.16)	1.22 (1.47 - 1.02)	0.031	0.074
iNKT CD4+	Ki67	-1.80 (-7.40, -0.83)	-0.12 (-0.98, 0.54)	1.67 (2.49 - 1.12)	0.011	0.034
iNKT CD4+	PD1	3.10 (1.00, 6.20)	-3.20 (-10.70, -1.30)	1.33 (1.69 - 1.05)	0.019	0.05
iNKT CD4+	TNFα	0.20 (0.16, 0.42)	0.04 (-0.17, 0.15)	1.12 (1.28 - 0.99)	0.077	0.14
iNKT CD8+	CD107a	0.18 (-0.05, 0.43)	0.91 (0.26, 1.28)	0.85 (1.07 - 0.68)	0.18	0.32
iNKT CD8+	CD25	1.43 (0.72, 2.54)	1.06 (0.89, 1.68)	1.28 (1.65 - 0.99)	0.058	0.11
iNKT CD8+	CD69	14.58 (8.40, 21.20)	3.20 (-6.20, 4.50)	1.55 (2.11 - 1.13)	0.0062	0.022

**iNKT CD8+**	GranzB	-1.60 (-6.20, -0.70)	-2.80 (-5.00, -0.50)	0.96 (1.32 - 0.70)	0.80	0.86
**iNKT CD8+**	IFNγ	0.47 (0.24, 1.74)	0.19 (-0.03, 0.39)	1.32 (1.77 - 0.99)	0.056	0.11
**iNKT CD8+**	IL10	0.08 (0.01, 0.37)	0.08 (-0.12, 0.23)	1.14 (1.30 - 1.01)	0.037	0.081
**iNKT CD8+**	IL17	-0.11 (-0.64, 0.12)	0.00 (-0.08, 0.18)	0.93 (1.22 - 0.71)	0.61	0.73
**iNKT CD8+**	Ki67	-0.15 (-0.64, 0.11)	0.03 (-0.54, 0.16)	1.10 (1.29 - 0.95)	0.21	0.35
**iNKT CD8+**	PD1	1.90 (1.10, 5.30)	-3.60 (-6.90, -1.00)	1.34 (1.57 - 1.14)	0.00038	0.0023
**MAIT MR1-**	CD25	1.19 (-1.22, 1.78)	0.57 (0.32, 1.65)	1.02 (1.27 - 0.82)	0.83	0.88
**MAIT MR1-**	CD69	0.40 (-0.20, 5.10)	-0.20 (-2.10, 1.80)	1.18 (1.52 - 0.91)	0.21	0.35
**MAIT MR1-**	GranzB	-0.17 (-0.47, 0.30)	1.00 (0.40, 1.50)	0.81 (1.11 - 0.59)	0.19	0.33
**MAIT MR1-**	PD1	1.42 (0.90, 1.80)	1.60 (0.40, 2.70)	1.17 (1.38 - 1.00)	0.05	0.10
**MAIT MR1+**	CD25	2.38 (0.98, 3.25)	2.18 (1.30, 3.14)	0.88 (1.29 - 0.60)	0.51	0.63
**MAIT MR1+**	CD69	12.60 (9.40, 15.10)	0.60 (-1.00, 3.10)	0.86 (1.29 - 0.57)	0.46	0.60
**MAIT MR1+**	GranzB	0.41 (0.08, 0.62)	0.79 (0.27, 2.01)	0.66 (0.84 - 0.53)	6e-04	0.0031
**MAIT MR1+**	IFNγ	0.78 (0.65, 2.25)	0.46 (0.25, 0.80)	1.10 (1.46 - 0.83)	0.49	0.63
**MAIT MR1+**	IL10	0.11 (-0.09, 0.32)	0.13 (-0.02, 0.41)	1.02 (1.11 - 0.93)	0.66	0.76
**MAIT MR1+**	PD1	0.57 (0.23, 0.99)	1.19 (0.35, 1.39)	1.00 (1.17 - 0.86)	0.95	0.97
**NK**	CD107a	3.24 (1.38, 3.46)	4.14 (2.28, 6.79)	0.85 (1.11 - 0.65)	0.22	0.36
**NK**	CD25	8.87 (7.39, 15.10)	5.61 (2.95, 8.61)	1.48 (2.01 - 1.10)	0.0099	0.032
**NK**	CD69	41.10 (30.00, 50.00)	11.80 (1.90, 16.60)	2.45 (3.71 - 1.61)	2.5e-05	0.00024
**NK**	GranzB	7.20 (5.40, 14.30)	-2.70 (-6.30, 0.80)	1.82 (2.38 - 1.39)	1.1e-05	0.00013
**NK**	IFNγ	3.74 (1.87, 5.55)	0.83 (0.29, 2.44)	1.66 (2.45 - 1.12)	0.011	0.034
**NK**	Ki67	-0.24 (-0.50, 0.09)	-0.33 (-0.73, -0.23)	1.12 (1.22 - 1.03)	0.011	0.034
**NK**	PD1	2.52 (1.97, 3.58)	2.14 (1.37, 2.64)	1.12 (1.40 - 0.90)	0.31	0.44
**NKT CD4+**	CD107a	3.72 (2.70, 7.70)	6.70 (2.68, 23.20)	0.80 (1.15 - 0.56)	0.23	0.37
**NKT CD4+**	CD25	7.89 (6.37, 12.50)	5.22 (3.73, 7.89)	1.38 (1.79 - 1.07)	0.013	0.038
**NKT CD4+**	CD69	13.30 (10.70, 24.90)	3.60 (1.80, 7.40)	1.56 (1.90 - 1.27)	1.5e-05	0.00016
**NKT CD4+**	GranzB	5.70 (0.90, 7.50)	-3.70 (-4.90, 1.00)	1.06 (1.30 - 0.86)	0.61	0.73
**NKT CD4+**	IFNγ	1.93 (1.26, 3.79)	0.74 (0.07, 1.86)	1.39 (1.89 - 1.02)	0.037	0.081
**NKT CD4+**	Ki67	-0.37 (-0.88, 0.63)	0.15 (0.02, 0.41)	0.99 (1.18 - 0.82)	0.88	0.91
**NKT CD4+**	PD1	2.10 (-1.50, 4.60)	0.50 (-0.90, 1.60)	1.06 (1.23 - 0.92)	0.42	0.56
**NKT CD8+**	CD107a	-0.47 (-1.90, -0.09)	1.87 (1.26, 3.50)	0.70 (0.95 - 0.52)	0.022	0.055
**NKT CD8+**	CD25	1.60 (0.48, 7.07)	1.70 (1.13, 3.00)	1.07 (1.72 - 0.67)	0.78	0.84
**NKT CD8+**	CD69	15.40 (6.90, 18.40)	4.80 (1.40, 4.92)	1.70 (2.13 - 1.35)	5.9e-06	9e-05
**NKT CD8+**	GranzB	7.50 (-0.60, 12.10)	-2.80 (-7.80, 0.10)	1.19 (1.88 - 0.75)	0.45	0.60
**NKT CD8+**	IL10	-0.34 (-1.87, 0.54)	0.24 (0.14, 0.43)	1.11 (1.32 - 0.93)	0.26	0.39
**NKT CD8+**	IL2	0.10 (-0.49, 0.36)	0.18 (0.06, 0.30)	1.02 (1.24 - 0.83)	0.87	0.91
**NKT CD8+**	Ki67	-0.37 (-0.64, -0.09)	-0.07 (-0.27, 0.00)	0.98 (1.11 - 0.86)	0.71	0.79
**NKT CD8+**	PD1	0.90 (-1.76, 1.50)	0.82 (-0.20, 1.43)	0.91 (1.14 - 0.72)	0.40	0.54
**Tconv CD4+**	CD107a	0.16 (0.07, 0.30)	0.37 (-0.07, 0.44)	0.87 (0.94 - 0.80)	0.00067	0.0033
**Tconv CD4+**	CD25	4.30 (2.70, 7.70)	4.20 (2.10, 5.70)	1.03 (1.24 - 0.85)	0.77	0.84
**Tconv CD4+**	CD69	4.20 (3.50, 7.90)	0.30 (-2.70, 2.60)	1.11 (1.34 - 0.92)	0.26	0.39
**Tconv CD4+**	GranzB	0.01 (-0.14, 0.10)	0.30 (0.14, 0.37)	0.87 (0.97 - 0.77)	0.015	0.042
**Tconv CD4+**	PD1	1.37 (0.98, 1.70)	2.00 (1.40, 3.10)	0.97 (1.07 - 0.88)	0.58	0.72
**Tconv CD8+**	CD25	0.51 (-0.04, 1.04)	0.26 (0.22, 0.65)	1.00 (1.17 - 0.85)	0.98	0.99
**Tconv CD8+**	CD69	1.10 (-0.80, 3.30)	-1.80 (-3.20, 2.50)	0.96 (1.13 - 0.82)	0.65	0.76
**Tconv CD8+**	GranzB	0.06 (-1.21, 0.90)	0.60 (0.40, 1.20)	0.89 (1.12 - 0.70)	0.31	0.44
**Tconv CD8+**	PD1	0.47 (0.10, 1.40)	1.50 (0.62, 2.20)	0.94 (1.06 - 0.82)	0.31	0.44
**γδT**	CD107a	0.69 (0.43, 1.15)	0.42 (0.29, 0.59)	1.14 (1.41 - 0.92)	0.24	0.38
**γδT**	CD25	1.71 (1.29, 2.85)	1.28 (0.40, 2.14)	1.21 (1.45 - 1.00)	0.049	0.10
**γδT**	CD69	24.00 (16.30, 29.40)	6.60 (-0.40, 8.30)	1.26 (1.80 - 0.89)	0.20	0.34
**γδT**	GranzB	1.80 (0.60, 3.10)	0.70 (-0.80, 1.60)	1.03 (1.13 - 0.93)	0.59	0.72
**γδT**	IFNγ	1.00 (0.64, 1.64)	0.64 (0.29, 1.18)	1.28 (1.63 - 1.01)	0.044	0.092
**γδT**	Ki67	0.07 (-0.15, 0.24)	0.03 (-0.11, 0.12)	1.07 (1.13 - 1.02)	0.0087	0.029
**γδT**	PD1	2.40 (1.30, 3.90)	2.80 (1.10, 3.40)	1.05 (1.13 - 0.97)	0.25	0.38

* Median (quartiles)

** Point estimate (95% Confidence interval)

Table S8. Frequency of cell subsets in LTBI-participants and BCG-recipients in unstimulated conditions related to Fig 6

Cell Subset	Marker	LTBI	BCG	Odds Ratio	p value	FDR p
**cDC1**	CD40	67.65 (63.53, 75.23)*	74.80 (54.99, 76.98)	1.85 (4.31 - 0.79)**	0.16	0.25
**cDC1**	CD80	6.98 (3.75, 9.59)	30.20 (22.12, 40.85)	0.23 (0.55 - 0.098)	0.00084	0.004
**cDC1**	CD83	47.55 (30.75, 59.85)	10.63 (1.97, 23.82)	3.57 (7.76 - 1.64)	0.0013	0.0058
**cDC1**	IL10	1.47 (0.16, 3.16)	2.10 (0.00, 4.09)	1.08 (1.52 - 0.77)	0.66	0.72
**cDC1**	IL27	33.55 (16.62, 41.25)	6.88 (0.00, 17.77)	2.56 (5.28 - 1.24)	0.011	0.029
**cDC1**	IL8	7.51 (5.25, 12.45)	27.60 (20.42, 43.88)	0.41 (0.81 - 0.21)	0.011	0.029
**cDC1**	PDL1	66.50 (33.05, 69.75)	88.45 (64.15, 94.30)	0.72 (1.83 - 0.29)	0.49	0.60
**cDC2**	CD40	55.85 (44.60, 71.30)	48.55 (42.05, 63.88)	1.26 (2.43 - 0.65)	0.50	0.60
**cDC2**	CD80	1.62 (0.93, 2.04)	3.47 (1.41, 6.19)	0.71 (1.13 - 0.45)	0.15	0.24
**cDC2**	CD83	17.30 (12.17, 21.93)	12.55 (4.43, 23.78)	1.08 (1.78 - 0.65)	0.77	0.82
**cDC2**	IL1β	2.82 (2.47, 3.73)	3.60 (3.20, 9.98)	0.48 (0.85 - 0.27)	0.011	0.029
**cDC2**	IL27	18.80 (11.55, 23.80)	8.65 (2.79, 11.67)	1.34 (2.27 - 0.79)	0.28	0.41
**cDC2**	IL8	12.70 (9.10, 18.65)	32.75 (22.40, 35.73)	0.27 (0.44 - 0.17)	6.6e-08	1.2e-06
**cDC2**	PDL1	66.35 (45.12, 73.17)	73.40 (54.15, 80.90)	0.69 (1.37 - 0.34)	0.29	0.42
**Mono**	CD40	35.65 (21.43, 43.95)	27.60 (23.95, 37.15)	0.85 (1.69 - 0.43)	0.65	0.71
**Mono**	CD80	6.19 (4.88, 7.25)	8.91 (4.77, 11.88)	0.86 (1.23 - 0.60)	0.40	0.54
**Mono**	CD83	1.00 (0.80, 1.15)	1.48 (1.24, 5.40)	0.45 (0.74 - 0.27)	0.0016	0.0065
**Mono**	GMCSF	0.41 (0.24, 0.49)	0.15 (0.11, 0.25)	1.10 (1.19 - 1.02)	0.0098	0.029
**Mono**	IL10	0.04 (0.03, 0.05)	0.30 (0.14, 0.48)	0.89 (0.94 - 0.83)	0.00013	0.00081
**Mono**	IL12p40	0.03 (0.02, 0.08)	0.03 (0.00, 0.24)	1.00 (1.04 - 0.95)	0.90	0.90
**Mono**	IL1β	3.66 (1.92, 9.72)	15.50 (13.15, 26.35)	0.22 (0.43 - 0.11)	1e-05	8.4e-05
**Mono**	IL27	0.45 (0.24, 0.72)	0.71 (0.32, 1.16)	0.90 (1.01 - 0.79)	0.083	0.15
**Mono**	IL8	41.30 (27.00, 54.20)	67.30 (62.27, 79.88)	0.36 (0.72 - 0.17)	0.0043	0.015
**Mono**	PDL1	39.45 (21.73, 58.38)	52.75 (42.25, 67.42)	0.49 (1.02 - 0.24)	0.057	0.11
**Mono**	TNFα	0.02 (0.01, 0.07)	0.15 (0.05, 0.21)	0.93 (0.98 - 0.88)	0.011	0.029
**pDC**	CD40	31.85 (23.50, 35.12)	29.90 (25.00, 31.85)	1.15 (1.84 - 0.71)	0.57	0.66
**pDC**	CD83	20.05 (11.85, 24.80)	15.95 (6.11, 23.85)	1.75 (3.10 - 0.98)	0.057	0.11
**pDC**	IL27	10.15 (6.44, 13.25)	8.07 (6.45, 14.87)	1.20 (1.95 - 0.74)	0.47	0.59
**pDC**	IL8	0.86 (0.60, 1.58)	2.72 (2.14, 6.49)	0.45 (0.72 - 0.28)	0.00086	0.004
**pDC**	PDL1	8.05 (5.20, 11.45)	15.55 (9.29, 23.77)	0.44 (0.82 - 0.24)	0.01	0.029
**iNKT CD4+**	CD107a	3.26 (2.25, 5.57)	3.01 (1.77, 8.87)	0.89 (1.45 - 0.55)	0.64	0.71
**iNKT CD4+**	CD25	11.80 (6.79, 15.40)	5.92 (2.67, 6.13)	1.81 (2.73 - 1.20)	0.0048	0.016
**iNKT CD4+**	CD69	44.20 (32.70, 49.80)	39.90 (36.30, 54.70)	0.84 (1.32 - 0.54)	0.46	0.59
**iNKT CD4+**	GMCSF	0.53 (0.24, 0.80)	0.00 (0.00, 0.24)	1.18 (1.32 - 1.06)	0.0026	0.01
**iNKT CD4+**	GranzB	37.70 (12.20, 45.60)	20.10 (18.70, 22.20)	1.64 (3.19 - 0.84)	0.15	0.24
**iNKT CD4+**	IFNγ	0.13 (0.10, 0.16)	0.07 (0.00, 0.30)	1.01 (1.09 - 0.93)	0.80	0.83
**iNKT CD4+**	IL10	0.69 (0.45, 0.96)	0.00 (0.00, 0.22)	1.30 (1.42 - 1.18)	2.9e-08	9e-07
**iNKT CD4+**	Ki67	15.50 (11.50, 22.20)	3.12 (1.72, 4.00)	3.55 (5.68 - 2.22)	1.3e-07	2e-06
**iNKT CD4+**	PD1	37.60 (32.70, 47.90)	24.40 (19.40, 30.60)	1.76 (2.91 - 1.07)	0.027	0.057
**iNKT CD4+**	TNFα	0.26 (0.13, 0.33)	0.18 (0.12, 0.63)	0.95 (1.08 - 0.84)	0.46	0.59
**iNKT CD8+**	CD107a	1.16 (0.77, 1.33)	5.22 (2.29, 9.22)	0.47 (0.76 - 0.29)	0.0025	0.0099
**iNKT CD8+**	CD25	0.54 (0.30, 0.65)	3.77 (0.85, 4.48)	0.58 (0.77 - 0.43)	0.00023	0.0014
**iNKT CD8+**	CD69	17.10 (12.80, 21.50)	39.00 (29.10, 46.10)	0.39 (0.61 - 0.25)	3.4e-05	0.00027
**iNKT CD8+**	GranzB	92.70 (86.70, 95.50)	57.40 (39.20, 63.20)	6.54 (11.47 - 3.73)	5.5e-11	6.9e-09
**iNKT CD8+**	IL10	0.20 (0.14, 0.35)	0.30 (0.14, 3.17)	0.73 (0.96 - 0.56)	0.023	0.052
**iNKT CD8+**	IL17	2.08 (1.01, 2.82)	0.30 (0.10, 0.86)	1.48 (1.88 - 1.16)	0.0014	0.006
**iNKT CD8+**	IL2	0.03 (0.01, 0.08)	0.22 (0.00, 1.09)	0.79 (0.96 - 0.64)	0.02	0.046
**iNKT CD8+**	Ki67	3.51 (2.36, 4.75)	2.31 (1.78, 3.65)	1.15 (1.47 - 0.90)	0.27	0.41
**iNKT CD8+**	PD1	27.30 (11.10, 42.20)	23.10 (22.20, 27.30)	1.28 (2.25 - 0.73)	0.39	0.52
**iNKT CD8+**	TNFα	0.10 (0.04, 0.15)	0.36 (0.00, 0.58)	0.89 (0.96 - 0.83)	0.0027	0.01
**MAIT MR1-**	CD107a	0.14 (0.10, 0.19)	0.67 (0.46, 1.44)	0.78 (0.89 - 0.69)	0.00013	0.00081
**MAIT MR1-**	CD25	2.98 (2.34, 3.83)	4.31 (2.73, 4.53)	0.95 (1.15 - 0.78)	0.57	0.66
**MAIT MR1-**	CD69	30.10 (25.10, 38.40)	50.80 (37.20, 67.70)	0.45 (0.70 - 0.28)	0.00049	0.0026

MAIT MR1-	GranzB	7.42 (3.17, 16.20)	2.71 (0.92, 9.26)	0.93 (1.84 - 0.47)	0.83	0.84
MAIT MR1-	IFNγ	0.03 (0.02, 0.03)	0.00 (0.00, 0.02)	0.98 (1.02 - 0.95)	0.32	0.45
MAIT MR1-	IL10	0.38 (0.25, 0.60)	0.06 (0.02, 0.09)	1.15 (1.21 - 1.10)	6.2e-08	1.2e-06
MAIT MR1-	IL2	0.02 (0.01, 0.03)	0.05 (0.02, 0.09)	0.98 (1.00 - 0.96)	0.051	0.10
MAIT MR1-	Ki67	0.45 (0.39, 0.62)	0.37 (0.19, 0.65)	0.94 (1.08 - 0.81)	0.39	0.52
MAIT MR1-	PD1	10.60 (8.02, 16.10)	9.99 (9.58, 19.10)	0.82 (1.19 - 0.56)	0.29	0.42
MAIT MR1-	TNFα	0.04 (0.03, 0.05)	0.03 (0.00, 0.19)	0.98 (1.01 - 0.95)	0.15	0.25
MAIT MR1+	CD107a	0.26 (0.12, 0.33)	1.13 (0.56, 1.68)	0.74 (0.86 - 0.64)	4.7e-05	0.00035
MAIT MR1+	CD25	0.35 (0.29, 0.41)	1.13 (0.64, 1.68)	0.70 (0.90 - 0.54)	0.0054	0.017
MAIT MR1+	CD69	72.90 (70.30, 74.80)	82.90 (77.40, 84.40)	0.53 (0.94 - 0.30)	0.029	0.06
MAIT MR1+	GranzB	0.15 (0.11, 0.29)	0.67 (0.53, 0.71)	0.89 (1.19 - 0.67)	0.44	0.57
MAIT MR1+	IFNγ	0.04 (0.01, 0.11)	0.10 (0.00, 0.35)	0.90 (1.00 - 0.81)	0.043	0.085
MAIT MR1+	IL10	0.47 (0.31, 0.62)	0.44 (0.17, 1.12)	0.97 (1.08 - 0.88)	0.59	0.67
MAIT MR1+	Ki67	0.30 (0.23, 0.37)	0.70 (0.26, 1.35)	0.84 (0.94 - 0.75)	0.0031	0.011
MAIT MR1+	PD1	1.10 (0.58, 1.97)	1.42 (0.70, 4.73)	0.70 (0.93 - 0.52)	0.015	0.036
NK	CD107a	2.43 (2.26, 3.43)	2.81 (1.91, 4.02)	1.13 (1.72 - 0.74)	0.58	0.66
NK	CD25	1.08 (0.80, 2.18)	2.18 (0.84, 3.57)	0.85 (1.34 - 0.54)	0.50	0.60
NK	CD69	41.10 (38.50, 47.20)	49.60 (37.80, 65.40)	0.50 (0.90 - 0.28)	0.021	0.046
NK	GMCSF	0.01 (0.01, 0.02)	0.10 (0.02, 0.16)	0.96 (0.98 - 0.94)	7.4e-07	9.2e-06
NK	GranzB	64.10 (52.90, 74.90)	82.70 (74.00, 84.40)	0.59 (0.95 - 0.37)	0.029	0.06
NK	IFNγ	0.16 (0.10, 0.20)	0.04 (0.03, 0.46)	0.94 (1.03 - 0.86)	0.21	0.33
NK	IL10	0.14 (0.12, 0.21)	0.06 (0.04, 0.10)	1.05 (1.10 - 1.01)	0.011	0.029
NK	Ki67	4.28 (3.79, 5.67)	3.41 (2.46, 4.83)	1.27 (1.65 - 0.98)	0.065	0.12
NK	PD1	1.22 (1.12, 2.05)	1.55 (1.17, 3.29)	0.91 (1.18 - 0.69)	0.47	0.59
NK	TNFα	0.09 (0.08, 0.13)	0.05 (0.03, 0.11)	1.01 (1.03 - 0.98)	0.57	0.66
NKT CD4+	CD107a	19.30 (9.48, 23.60)	15.60 (7.31, 21.80)	1.08 (1.90 - 0.61)	0.80	0.83
NKT CD4+	CD25	10.40 (8.13, 16.10)	12.00 (6.53, 14.20)	1.14 (1.77 - 0.73)	0.56	0.66
NKT CD4+	CD69	39.80 (27.00, 53.60)	41.00 (35.60, 46.80)	0.97 (1.56 - 0.60)	0.90	0.90
NKT CD4+	GranzB	18.50 (11.40, 32.80)	31.70 (25.90, 48.40)	0.52 (0.90 - 0.30)	0.02	0.046
NKT CD4+	IL10	0.50 (0.24, 0.86)	0.06 (0.00, 0.23)	1.21 (1.42 - 1.03)	0.019	0.044
NKT CD4+	Ki67	3.16 (1.82, 3.78)	2.28 (1.46, 2.70)	1.24 (1.84 - 0.83)	0.29	0.42
NKT CD4+	PD1	24.40 (14.70, 36.70)	18.20 (9.42, 26.40)	1.87 (3.23 - 1.09)	0.024	0.052
NKT CD8+	CD107a	6.48 (3.59, 11.40)	9.29 (5.11, 29.00)	0.61 (1.12 - 0.33)	0.11	0.19
NKT CD8+	CD25	4.82 (1.58, 6.65)	4.13 (1.55, 11.00)	0.87 (1.43 - 0.53)	0.58	0.66
NKT CD8+	CD69	24.00 (16.90, 34.70)	31.30 (25.20, 47.60)	0.70 (1.08 - 0.45)	0.11	0.19
NKT CD8+	GranzB	55.40 (37.20, 70.60)	49.30 (47.10, 71.80)	0.91 (1.66 - 0.50)	0.77	0.82
NKT CD8+	IL10	3.92 (1.48, 7.78)	0.85 (0.45, 5.36)	1.20 (2.03 - 0.71)	0.49	0.60
NKT CD8+	IL2	1.17 (0.74, 1.62)	0.39 (0.23, 4.64)	0.77 (1.19 - 0.50)	0.25	0.38
NKT CD8+	Ki67	1.94 (1.17, 3.31)	1.32 (0.52, 2.14)	1.32 (1.81 - 0.96)	0.091	0.16
NKT CD8+	PD1	10.70 (4.74, 14.20)	10.10 (8.15, 13.60)	1.06 (1.72 - 0.66)	0.81	0.83
Tconv CD4+	CD107a	0.26 (0.18, 0.35)	0.97 (0.66, 1.34)	0.77 (0.85 - 0.70)	1.4e-07	2e-06
Tconv CD4+	CD25	10.50 (8.25, 11.30)	11.60 (9.83, 17.60)	0.76 (0.94 - 0.61)	0.012	0.029
Tconv CD4+	CD69	36.60 (29.40, 40.20)	53.00 (45.80, 67.00)	0.42 (0.65 - 0.27)	0.00011	0.00076
Tconv CD4+	GMCSF	0.00 (0.00, 0.00)	0.01 (0.00, 0.02)	0.99 (0.99 - 0.99)	2e-06	2.3e-05
Tconv CD4+	GranzB	0.92 (0.18, 1.66)	1.64 (0.80, 2.39)	0.94 (1.34 - 0.66)	0.75	0.81
Tconv CD4+	IFNγ	0.02 (0.02, 0.03)	0.02 (0.01, 0.03)	1.00 (1.00 - 0.99)	0.33	0.45
Tconv CD4+	IL10	0.11 (0.07, 0.13)	0.02 (0.02, 0.03)	1.04 (1.05 - 1.03)	6e-10	2.5e-08
Tconv CD4+	IL17	0.00 (0.00, 0.00)	0.00 (0.00, 0.01)	1.00 (1.00 - 1.00)	0.26	0.40
Tconv CD4+	IL2	0.00 (0.00, 0.00)	0.01 (0.00, 0.01)	1.00 (1.00 - 1.00)	0.00039	0.0022
Tconv CD4+	Ki67	0.89 (0.63, 0.98)	0.52 (0.45, 0.74)	1.12 (1.21 - 1.04)	0.003	0.011
Tconv CD4+	PD1	13.00 (7.41, 14.90)	14.00 (13.00, 15.00)	0.80 (1.06 - 0.60)	0.12	0.20
Tconv CD4+	TNFα	0.00 (0.00, 0.00)	0.01 (0.01, 0.01)	1.00 (1.00 - 0.99)	0.01	0.029
Tconv CD8+	CD107a	0.26 (0.17, 0.33)	0.78 (0.55, 1.02)	0.81 (0.89 - 0.74)	3.3e-06	3.2e-05
Tconv CD8+	CD25	0.70 (0.50, 1.16)	0.71 (0.46, 1.80)	0.88 (1.11 - 0.70)	0.28	0.41
Tconv CD8+	CD69	24.10 (14.40, 34.20)	47.30 (34.30, 65.80)	0.34 (0.53 - 0.22)	2.7e-06	2.8e-05
Tconv CD8+	GranzB	9.16 (6.08, 17.80)	15.10 (11.70, 19.70)	0.85 (1.37 - 0.53)	0.51	0.61
Tconv CD8+	IFNγ	0.02 (0.01, 0.03)	0.02 (0.02, 0.04)	0.99 (1.00 - 0.98)	0.059	0.11

**Tconv CD8+**	IL10	0.11 (0.08, 0.17)	0.03 (0.02, 0.05)	1.04 (1.06 - 1.02)	6.6e-06	5.9e-05
**Tconv CD8+**	IL2	0.01 (0.01, 0.02)	0.03 (0.01, 0.06)	0.99 (0.99 - 0.98)	0.00087	0.004
**Tconv CD8+**	Ki67	0.42 (0.31, 0.51)	0.39 (0.35, 0.45)	1.03 (1.11 - 0.96)	0.34	0.47
**Tconv CD8+**	PD1	8.88 (6.44, 11.80)	10.10 (9.21, 12.10)	0.96 (1.32 - 0.70)	0.81	0.83
**Tconv CD8+**	TNFα	0.00 (0.00, 0.01)	0.01 (0.01, 0.01)	1.00 (1.00 - 0.99)	0.0065	0.02
**γδT**	CD107a	0.45 (0.26, 0.58)	0.84 (0.71, 20.00)	0.41 (0.76 - 0.22)	0.0045	0.015
**γδT**	CD25	0.63 (0.49, 0.97)	0.39 (0.22, 15.00)	0.53 (0.88 - 0.32)	0.015	0.036
**γδT**	CD69	30.80 (23.70, 33.60)	62.60 (47.80, 70.10)	0.22 (0.36 - 0.14)	3e-10	1.9e-08
**γδT**	GranzB	32.60 (29.50, 48.80)	49.90 (26.70, 60.50)	0.64 (1.12 - 0.36)	0.12	0.20
**γδT**	IFNγ	0.06 (0.05, 0.08)	0.07 (0.04, 7.11)	0.51 (0.85 - 0.30)	0.011	0.029
**γδT**	IL10	0.49 (0.29, 0.57)	0.10 (0.06, 0.16)	1.15 (1.21 - 1.09)	6.9e-08	1.2e-06
**γδT**	IL17	0.53 (0.34, 0.75)	0.09 (0.03, 0.22)	1.23 (1.49 - 1.02)	0.03	0.061
**γδT**	Ki67	1.15 (0.81, 1.29)	0.96 (0.44, 1.17)	1.12 (1.28 - 0.98)	0.11	0.19
**γδT**	PD1	18.10 (13.20, 24.70)	12.80 (7.70, 41.40)	0.88 (1.48 - 0.52)	0.63	0.70
**γδT**	TNFα	0.02 (0.00, 0.03)	0.08 (0.03, 0.47)	0.89 (0.95 - 0.83)	0.00079	0.004

* Median (quartiles)

** Point estimate (95% Confidence interval)

Table S9. Frequency of cell subsets in LTBI-participants and BCG-recipients in Mtb-stimulated conditions related to Fig 6

Cell Subset	Marker	LTBI	BCG	Odds Ratio	p value	FDR p
**cDC1**	CD40	94.10 (91.72, 95.65)*	91.50 (85.97, 96.03)	1.55 (3.89 - 0.62)**	0.35	0.52
**cDC1**	CD80	10.18 (8.23, 12.60)	7.79 (3.20, 10.23)	1.46 (2.36 - 0.91)	0.12	0.23
**cDC1**	CD83	57.35 (49.83, 61.20)	48.75 (29.25, 56.68)	3.21 (6.49 - 1.59)	0.0011	0.0065
**cDC1**	IL10	0.58 (0.41, 0.97)	0.78 (0.00, 3.48)	0.84 (1.13 - 0.63)	0.26	0.41
**cDC1**	IL1β	40.05 (25.82, 52.20)	72.85 (63.38, 76.58)	0.65 (1.37 - 0.31)	0.25	0.41
**cDC1**	IL27	72.50 (66.68, 77.95)	55.20 (48.73, 58.43)	1.67 (3.69 - 0.75)	0.21	0.37
**cDC1**	IL8	49.15 (40.23, 55.58)	83.40 (78.42, 85.00)	0.63 (1.34 - 0.29)	0.23	0.38
**cDC1**	PDL1	93.65 (92.08, 95.97)	97.75 (88.45, 99.20)	1.34 (3.44 - 0.52)	0.55	0.66
**cDC1**	TNFα	1.25 (0.66, 2.04)	3.27 (0.00, 8.71)	0.74 (1.11 - 0.49)	0.14	0.26
**cDC2**	CD40	92.80 (87.98, 94.50)	87.95 (58.80, 88.70)	3.03 (7.58 - 1.21)	0.018	0.055
**cDC2**	CD80	1.21 (0.62, 1.39)	2.24 (0.80, 8.37)	0.73 (1.00 - 0.53)	0.05	0.13
**cDC2**	CD83	29.55 (24.55, 34.72)	29.70 (22.80, 35.02)	1.23 (1.86 - 0.81)	0.33	0.49
**cDC2**	GMCSF	0.57 (0.33, 0.76)	1.32 (0.56, 2.49)	0.81 (1.04 - 0.62)	0.099	0.21
**cDC2**	IL10	1.75 (0.84, 2.18)	2.58 (0.84, 3.82)	0.93 (1.24 - 0.70)	0.63	0.73
**cDC2**	IL12p40	0.17 (0.11, 0.55)	0.88 (0.16, 1.92)	0.82 (1.00 - 0.67)	0.047	0.12
**cDC2**	IL1β	72.55 (67.88, 76.55)	59.90 (55.70, 64.28)	1.64 (2.31 - 1.17)	0.0046	0.02
**cDC2**	IL27	78.40 (72.75, 81.92)	51.75 (47.60, 58.90)	3.68 (6.67 - 2.03)	1.7e-05	0.00017
**cDC2**	IL8	82.25 (79.40, 83.85)	85.85 (71.78, 87.40)	1.15 (1.84 - 0.73)	0.54	0.66
**cDC2**	PDL1	98.65 (98.12, 99.27)	92.65 (81.80, 95.57)	2.63 (5.43 - 1.27)	0.0093	0.032
**cDC2**	TNFα	2.34 (2.08, 3.47)	3.50 (2.18, 8.02)	0.96 (1.42 - 0.64)	0.82	0.84
**Mono**	CD40	55.15 (42.12, 67.25)	62.00 (46.83, 76.25)	0.88 (1.76 - 0.44)	0.72	0.80
**Mono**	CD80	0.79 (0.57, 1.27)	1.06 (0.81, 2.07)	0.75 (1.09 - 0.52)	0.13	0.24
**Mono**	CD83	14.25 (7.69, 18.20)	15.90 (11.57, 19.38)	0.74 (1.05 - 0.52)	0.092	0.21
**Mono**	IL10	3.42 (1.56, 5.16)	1.04 (0.83, 1.39)	1.70 (2.32 - 1.25)	0.00075	0.0057
**Mono**	IL1β	97.95 (97.05, 98.75)	97.20 (88.45, 98.35)	2.03 (4.90 - 0.84)	0.11	0.22
**Mono**	IL27	6.24 (4.41, 7.83)	2.19 (1.44, 4.54)	1.78 (2.65 - 1.19)	0.0049	0.02
**Mono**	IL8	95.55 (93.52, 96.10)	96.20 (89.70, 97.12)	1.68 (3.80 - 0.74)	0.21	0.37
**Mono**	PDL1	99.40 (99.05, 99.50)	98.30 (86.90, 99.15)	2.00 (4.58 - 0.88)	0.10	0.21
**Mono**	TNFα	0.65 (0.43, 1.30)	0.76 (0.58, 1.12)	1.05 (1.28 - 0.87)	0.60	0.71
**pDC**	CD40	67.45 (65.43, 75.05)	70.30 (49.52, 77.00)	1.73 (3.13 - 0.95)	0.072	0.17
**pDC**	CD80	2.25 (1.42, 2.98)	4.08 (3.67, 4.56)	0.88 (1.20 - 0.64)	0.42	0.56
**pDC**	CD83	25.90 (20.15, 29.85)	33.45 (13.24, 49.78)	1.12 (1.97 - 0.64)	0.68	0.78
**pDC**	IL10	11.70 (10.01, 26.68)	15.90 (12.73, 20.27)	1.08 (1.94 - 0.61)	0.78	0.82
**pDC**	IL1β	30.25 (27.02, 40.03)	25.45 (16.10, 28.27)	2.09 (3.49 - 1.25)	0.0049	0.02
**pDC**	IL27	8.47 (6.64, 12.25)	22.70 (16.88, 26.62)	0.44 (0.60 - 0.32)	3.1e-07	6.7e-06
**pDC**	IL8	73.40 (67.67, 82.15)	90.00 (69.10, 93.72)	0.77 (1.62 - 0.37)	0.49	0.64
**pDC**	PDL1	1.99 (1.31, 2.65)	0.72 (0.31, 0.79)	1.57 (1.91 - 1.30)	4.4e-06	6.6e-05
**pDC**	TNFα	2.25 (1.42, 2.98)	4.08 (3.67, 4.56)	0.88 (1.20 - 0.64)	0.42	0.56
**iNKT CD4+**	CD107a	7.17 (6.61, 10.10)	6.15 (3.26, 14.50)	1.08 (1.67 - 0.70)	0.72	0.80
**iNKT CD4+**	CD25	24.40 (19.10, 32.60)	9.19 (7.37, 19.50)	2.23 (3.68 - 1.36)	0.0016	0.0086
**iNKT CD4+**	CD69	60.80 (45.60, 66.10)	50.40 (36.50, 53.80)	1.53 (2.30 - 1.02)	0.039	0.11
**iNKT CD4+**	GMCSF	1.57 (1.27, 3.08)	0.24 (0.13, 0.34)	1.69 (2.38 - 1.20)	0.0026	0.013
**iNKT CD4+**	GranzB	34.50 (12.50, 43.40)	21.00 (18.90, 24.70)	1.41 (2.72 - 0.74)	0.30	0.46
**iNKT CD4+**	IFNγ	2.25 (1.93, 3.28)	0.99 (0.77, 11.80)	0.97 (1.65 - 0.57)	0.91	0.91
**iNKT CD4+**	IL10	1.82 (1.07, 2.41)	0.09 (0.00, 0.13)	1.69 (2.02 - 1.42)	5.4e-09	2.9e-07
**iNKT CD4+**	IL2	0.38 (0.34, 1.82)	0.45 (0.22, 1.74)	1.03 (1.26 - 0.85)	0.74	0.81
**iNKT CD4+**	Ki67	10.80 (8.43, 15.90)	2.53 (2.25, 5.60)	2.51 (3.73 - 1.69)	5.1e-06	6.8e-05
**iNKT CD4+**	PD1	43.70 (35.80, 54.30)	29.70 (18.30, 44.80)	1.72 (2.77 - 1.07)	0.026	0.078
**iNKT CD4+**	TNFα	0.47 (0.35, 0.82)	0.56 (0.32, 0.80)	0.93 (1.12 - 0.77)	0.44	0.57
**iNKT CD8+**	CD107a	1.36 (0.79, 2.31)	12.00 (3.81, 16.70)	0.39 (0.64 - 0.24)	0.00017	0.0015
**iNKT CD8+**	CD25	2.31 (1.02, 3.08)	7.39 (2.88, 9.45)	0.59 (0.87 - 0.40)	0.0082	0.031
**iNKT CD8+**	CD69	33.10 (24.70, 41.90)	42.50 (38.40, 54.70)	0.67 (1.02 - 0.44)	0.065	0.16
**iNKT CD8+**	GranzB	91.50 (81.40, 94.20)	50.30 (34.50, 66.10)	4.42 (8.42 - 2.32)	6.4e-06	7.5e-05

**iNKT CD8+**	IFNγ	0.52 (0.29, 1.74)	1.08 (0.46, 2.80)	0.90 (1.27 - 0.63)	0.54	0.66
**iNKT CD8+**	IL10	1.79 (1.01, 2.65)	0.69 (0.13, 2.80)	1.15 (1.63 - 0.81)	0.43	0.57
**iNKT CD8+**	IL17	3.17 (2.30, 4.56)	3.31 (2.10, 4.07)	0.96 (1.25 - 0.74)	0.77	0.82
**iNKT CD8+**	Ki67	26.50 (17.70, 44.10)	29.10 (24.00, 34.80)	1.21 (2.12 - 0.69)	0.50	0.64
**iNKT CD8+**	PD1	4.35 (2.75, 5.31)	6.21 (4.41, 7.93)	0.79 (1.01 - 0.61)	0.065	0.16
**MAIT MR1-**	CD25	36.00 (27.90, 43.20)	52.90 (44.90, 64.60)	0.51 (0.75 - 0.35)	0.00055	0.0045
**MAIT MR1-**	CD69	6.91 (3.00, 17.10)	2.39 (1.73, 9.81)	0.91 (1.84 - 0.45)	0.80	0.83
**MAIT MR1-**	GranzB	0.38 (0.27, 0.55)	0.13 (0.06, 0.83)	0.99 (1.13 - 0.86)	0.85	0.86
**MAIT MR1-**	PD1	12.10 (9.00, 21.10)	13.10 (11.70, 19.90)	0.84 (1.24 - 0.57)	0.39	0.54
**MAIT MR1+**	CD25	2.73 (2.27, 3.58)	8.27 (3.18, 9.43)	0.56 (0.79 - 0.40)	0.00092	0.0059
**MAIT MR1+**	CD69	85.50 (83.30, 87.60)	85.10 (83.30, 86.80)	0.91 (1.26 - 0.66)	0.59	0.70
**MAIT MR1+**	GranzB	0.61 (0.45, 0.82)	2.55 (1.28, 5.03)	0.57 (0.83 - 0.38)	0.0039	0.019
**MAIT MR1+**	IFNγ	0.84 (0.74, 2.25)	1.85 (0.86, 7.31)	0.67 1.10 - 0.41)	0.11	0.22
**MAIT MR1+**	IL10	0.53 (0.42, 0.85)	0.43 (0.19, 1.79)	0.81 (.14 - 0.57)	0.22	0.37
**MAIT MR1+**	PD1	1.43 (0.66, 2.18)	1.16 (0.77, 6.05)	0.76 (1.05 - 0.55)	0.093	0.21
**NK**	CD107a	5.65 (4.18, 9.21)	3.99 (3.28, 5.97)	1.30 (2.06 - 0.82)	0.26	0.41
**NK**	CD25	9.72 (8.38, 15.90)	4.96 (3.58, 7.80)	1.69 (2.68 - 1.06)	0.027	0.078
**NK**	CD69	80.90 (76.60, 88.90)	71.10 (57.50, 81.20)	1.85 (2.96 - 1.15)	0.01	0.035
**NK**	GranzB	73.40 (69.60, 86.60)	82.00 (80.20, 83.60)	0.82 (1.31 - 0.51)	0.40	0.55
**NK**	IFNγ	3.91 (2.25, 5.71)	2.03 (1.13, 3.90)	1.41 (2.11 - 0.94)	0.096	0.21
**NK**	Ki67	4.04 (3.70, 5.13)	3.29 (2.64, 4.63)	1.21 (1.51 - 0.96)	0.11	0.21
**NK**	PD1	4.17 (3.18, 7.05)	3.83 (2.93, 4.74)	1.18 (1.60 - 0.88)	0.27	0.43
**NKT CD4+**	CD107a	23.80 (13.20, 31.10)	18.00 (11.80, 30.90)	0.92 (1.53 - 0.56)	0.75	0.81
**NKT CD4+**	CD25	18.20 (15.80, 29.90)	23.60 (14.20, 28.10)	1.14 (1.73 - 0.75)	0.53	0.66
**NKT CD4+**	CD69	56.80 (47.40, 70.40)	50.00 (44.40, 58.60)	1.23 (1.90 - 0.79)	0.36	0.52
**NKT CD4+**	GranzB	26.40 (19.40, 33.90)	30.00 (22.20, 40.20)	0.78 (1.17 - 0.51)	0.23	0.38
**NKT CD4+**	IFNγ	1.93 (1.26, 3.79)	2.04 (1.69, 4.03)	1.05 (1.46 - 0.75)	0.77	0.82
**NKT CD4+**	Ki67	1.05 (0.77, 1.95)	0.34 (0.10, 0.49)	1.39 (1.69 - 1.15)	0.00087	0.0059
**NKT CD4+**	PD1	2.46 (2.10, 3.27)	1.80 (1.62, 2.77)	1.23 (1.52 - 0.99)	0.062	0.16
**NKT CD8+**	CD107a	20.10 (16.40, 35.90)	22.70 (10.10, 24.20)	1.84 (3.17 - 1.07)	0.027	0.079
**NKT CD8+**	CD25	6.02 (2.82, 7.36)	21.50 (17.40, 38.90)	0.26 (0.47 - 0.14)	7.3e-06	7.7e-05
**NKT CD8+**	CD69	4.03 (2.81, 12.90)	11.90 (8.75, 23.30)	0.54 (0.85 - 0.34)	0.0087	0.032
**NKT CD8+**	GranzB	42.40 (22.50, 57.80)	33.40 (29.50, 50.20)	1.06 (1.79 - 0.63)	0.81	0.84
**NKT CD8+**	IL10	67.20 (51.50, 70.20)	48.60 (24.70, 67.00)	1.80 (3.20 - 1.01)	0.046	0.12
**NKT CD8+**	IL2	0.58 (0.16, 2.38)	0.30 (0.17, 0.91)	1.17 (1.64 - 0.83)	0.37	0.52
**NKT CD8+**	Ki67	2.97 (1.57, 7.13)	2.56 (0.74, 10.90)	0.79 (1.32 - 0.48)	0.37	0.52
**NKT CD8+**	PD1	0.71 (0.41, 1.27)	1.32 (0.45, 10.90)	0.56 (0.90 - 0.35)	0.016	0.052
**Tconv CD4+**	CD107a	1.53 (1.22, 2.71)	1.32 (0.84, 1.69)	1.12 (1.41 - 0.90)	0.31	0.46
**Tconv CD4+**	CD25	12.00 (5.26, 19.60)	9.96 (6.43, 15.60)	1.09 (1.80 - 0.65)	0.75	0.81
**Tconv CD4+**	CD69	0.43 (0.32, 0.53)	1.47 (1.17, 1.72)	0.72 (0.80 - 0.65)	4.1e-10	4.4e-08
**Tconv CD4+**	GranzB	15.90 (13.60, 17.90)	20.80 (18.80, 26.20)	0.67 (0.85 - 0.53)	0.00095	0.0059
**Tconv CD4+**	PD1	40.40 (32.40, 45.20)	60.50 (54.80, 67.10)	0.43 (0.61 - 0.31)	1.3e-06	2.3e-05
**Tconv CD8+**	CD25	15.10 (8.18, 16.20)	16.40 (16.10, 16.90)	0.79 (1.03 - 0.60)	0.083	0.19
**Tconv CD8+**	CD69	1.19 (0.71, 2.27)	2.55 (2.16, 4.05)	0.67 (0.86 - 0.53)	0.0012	0.0065
**Tconv CD8+**	GranzB	24.70 (20.40, 31.90)	52.40 (43.00, 64.90)	0.36 (0.51 - 0.25)	2.1e-08	7.3e-07
**Tconv CD8+**	PD1	9.43 (7.24, 18.70)	17.10 (13.20, 20.70)	0.82 (1.33 - 0.50)	0.41	0.56
**γδT**	CD107a	10.80 (6.77, 12.00)	10.80 (10.30, 13.80)	0.93 (1.27 - 0.68)	0.64	0.74
**γδT**	CD25	1.19 (1.01, 1.60)	2.99 (1.15, 24.50)	0.42 (0.80 - 0.22)	0.009	0.032
**γδT**	CD69	2.47 (1.97, 3.80)	3.64 (2.46, 17.80)	0.51 (0.82 - 0.32)	0.0052	0.02
**γδT**	GranzB	53.00 (46.60, 54.80)	66.70 (63.50, 78.30)	0.38 (0.55 - 0.27)	9.8e-08	2.6e-06
**γδT**	IFNγ	36.40 (33.40, 46.10)	57.30 (30.70, 65.00)	0.64 (1.10 - 0.37)	0.10	0.21
**γδT**	Ki67	1.12 (0.68, 1.77)	4.53 (1.94, 10.00)	0.47 (0.79 - 0.28)	0.0044	0.02
**γδT**	PD1	22.10 (15.60, 28.20)	14.40 (14.20, 45.70)	1.11 (1.23 - 1.00)	0.06	0.14

* Median (quartiles)
** Point estimate (95% Confidence interval)

Table S10. Frequency of cell subsets in LTBI-participants and BCG-recipients in Mtb-stimulated conditions after background subtraction related to Fig 6

Cell Subset	Marker	LTBI	BCG	Odds Ratio	p value	FDR p
**cDC1**	CD40	23.15 (17.30, 28.95)*	16.55 (8.55, 20.22)	1.37 (3.43 - 0.55)**	0.50	0.63
**cDC1**	CD80	1.51 (0.41, 9.40)	-23.93 (-33.01, -8.94)	1.07 (1.92 - 0.60)	0.82	0.86
**cDC1**	CD83	11.00 (0.13, 19.07)	16.20 (0.00, 44.73)	2.16 (4.64 - 1.00)	0.049	0.13
**cDC1**	IL10	-0.82 (-2.26, -0.09)	-0.74 (-2.16, 0.05)	0.85 (1.13 - 0.64)	0.26	0.41
**cDC1**	IL1β	39.32 (24.41, 51.62)	65.72 (51.88, 70.05)	0.96 (2.31 - 0.40)	0.93	0.96
**cDC1**	IL27	38.75 (31.00, 56.30)	38.66 (18.08, 51.90)	1.54 (3.63 - 0.65)	0.32	0.44
**cDC1**	IL8	40.20 (30.92, 46.23)	51.75 (19.32, 62.60)	1.31 (2.95 - 0.58)	0.52	0.65
**cDC1**	PDL1	29.00 (26.00, 49.30)	6.65 (2.48, 13.68)	4.77 (9.54 - 2.39)	9.7e-06	0.00015
**cDC1**	TNFα	-0.66 (-2.19, 0.75)	3.27 (0.00, 8.63)	0.77 (1.25 - 0.47)	0.28	0.43
**cDC2**	CD40	34.55 (21.75, 44.57)	28.10 (11.02, 44.30)	3.09 (7.65 - 1.25)	0.015	0.06
**cDC2**	CD80	-0.45 (-1.23, 0.08)	-0.89 (-1.73, 0.58)	0.83 (1.09 - 0.63)	0.19	0.32
**cDC2**	CD83	13.41 (8.05, 17.27)	19.30 (5.12, 24.84)	1.22 (1.76 - 0.85)	0.29	0.43
**cDC2**	GMCSF	0.52 (0.25, 0.70)	0.88 (0.51, 1.86)	0.81 (1.04 - 0.62)	0.099	0.19
**cDC2**	IL10	1.32 (0.84, 1.89)	1.13 (0.00, 2.45)	1.00 (1.30 - 0.77)	1.00	1.00
**cDC2**	IL12p40	0.12 (0.10, 0.55)	0.88 (0.16, 1.92)	0.82 (0.99 - 0.67)	0.043	0.13
**cDC2**	IL1β	68.48 (65.36, 72.32)	55.20 (43.85, 57.11)	1.58 (2.34 - 1.06)	0.024	0.086
**cDC2**	IL27	58.97 (57.15, 60.70)	41.64 (25.97, 50.52)	3.51 (6.05 - 2.04)	5.6e-06	9.9e-05
**cDC2**	IL8	69.90 (63.95, 74.21)	48.25 (29.53, 61.38)	1.92 (3.72 - 0.99)	0.054	0.13
**cDC2**	PDL1	31.20 (25.75, 51.92)	20.10 (9.13, 25.78)	7.03 (12.39 - 3.99)	1.5e-11	1.6e-09
**cDC2**	TNFα	2.29 (1.91, 3.14)	2.94 (1.71, 8.02)	0.83 (1.22 - 0.57)	0.35	0.48
**Mono**	CD40	20.85 (5.25, 28.85)	27.35 (9.18, 31.90)	1.01 (1.69 - 0.61)	0.96	0.98
**Mono**	CD80	-4.96 (-5.82, -4.02)	-7.81 (-9.62, -2.86)	0.79 (1.16 - 0.54)	0.22	0.37
**Mono**	CD83	13.30 (6.93, 17.19)	10.20 (8.74, 14.19)	0.93 (1.36 - 0.63)	0.71	0.81
**Mono**	IL10	3.38 (1.51, 5.12)	0.86 (0.45, 1.15)	1.73 (2.60 - 1.14)	0.0092	0.042
**Mono**	IL1β	93.17 (88.75, 95.88)	77.00 (32.65, 84.03)	4.09 (12.69 - 1.32)	0.015	0.06
**Mono**	IL27	5.53 (3.63, 7.58)	1.77 (0.81, 3.31)	1.83 (2.79 - 1.20)	0.0052	0.031
**Mono**	IL8	52.85 (42.95, 66.25)	20.40 (4.38, 28.62)	10.7 (22.6 - 5.06)	5.6e-10	3e-08
**Mono**	PDL1	60.05 (41.17, 77.50)	36.40 (17.62, 45.42)	4.29 (9.92 - 1.86)	0.00066	0.0058
**Mono**	TNFα	0.63 (0.41, 1.26)	0.58 (0.34, 1.05)	1.08 (1.35 - 0.87)	0.49	0.63
**pDC**	CD40	35.05 (31.95, 50.28)	38.35 (16.57, 49.45)	1.72 (3.05 - 0.96)	0.066	0.15
**pDC**	CD80	2.00 (0.42, 2.96)	2.99 (1.99, 3.55)	0.96 (1.31 - 0.71)	0.80	0.86
**pDC**	CD83	6.85 (-5.52, 16.80)	12.79 (5.67, 23.53)	0.97 (1.71 - 0.56)	0.93	0.96
**pDC**	IL1β	11.70 (9.87, 26.68)	13.84 (10.29, 19.84)	0.66 (1.47 - 0.30)	0.31	0.44
**pDC**	IL27	21.01 (14.35, 32.39)	12.48 (8.84, 15.59)	1.91 (3.07 - 1.18)	0.0079	0.039
**pDC**	IL8	7.87 (5.49, 11.15)	21.02 (11.41, 23.75)	0.36 (0.51 - 0.26)	7.2e-09	2.5e-07
**pDC**	PDL1	65.23 (56.88, 71.34)	70.81 (40.15, 77.17)	0.78 (1.87 - 0.32)	0.57	0.70
**pDC**	TNFα	1.18 (0.49, 1.79)	0.09 (-0.08, 0.76)	1.56 (1.90 - 1.27)	1.5e-05	0.00018
**iNKT CD4+**	CD107a	4.21 (3.57, 4.56)	0.46 (-0.40, 2.82)	1.37 (1.87 - 1.01)	0.041	0.12
**iNKT CD4+**	CD25	14.57 (3.80, 22.10)	2.38 (0.10, 16.27)	1.82 (3.19 - 1.04)	0.036	0.12
**iNKT CD4+**	CD69	14.30 (10.70, 18.90)	0.80 (-1.60, 4.80)	1.68 (2.36 - 1.19)	0.0028	0.02
**iNKT CD4+**	GMCSF	1.06 (0.75, 2.93)	0.13 (0.00, 0.19)	1.38 (1.96 - 0.97)	0.073	0.15
**iNKT CD4+**	GranzB	-1.26 (-8.60, 0.10)	0.30 (-2.40, 1.60)	0.80 (1.08 - 0.60)	0.15	0.27
**iNKT CD4+**	IFNγ	1.97 (1.56, 3.03)	0.68 (0.24, 11.80)	0.92 (1.52 - 0.56)	0.75	0.84
**iNKT CD4+**	IL10	0.82 (0.63, 1.61)	0.00 (-0.13, 0.10)	1.33 (1.63 - 1.08)	0.0062	0.034
**iNKT CD4+**	IL2	0.38 (0.33, 1.32)	0.28 (0.10, 1.74)	1.03 (1.26 - 0.85)	0.77	0.84
**iNKT CD4+**	Ki67	-1.80 (-7.40, -0.83)	0.80 (0.06, 0.81)	1.66 (2.68 - 1.03)	0.038	0.12
**iNKT CD4+**	PD1	3.10 (1.00, 6.20)	-0.70 (-1.10, 3.49)	1.15 (1.50 - 0.88)	0.29	0.43
**iNKT CD4+**	TNFα	0.20 (0.16, 0.42)	0.25 (0.15, 0.60)	0.96 (1.14 - 0.80)	0.61	0.73
**iNKT CD8+**	CD107a	0.18 (-0.05, 0.43)	2.01 (0.78, 6.78)	0.56 (0.81 - 0.38)	0.0023	0.018
**iNKT CD8+**	CD25	1.43 (0.72, 2.54)	2.29 (0.50, 5.44)	0.88 (1.37 - 0.57)	0.58	0.70
**iNKT CD8+**	CD69	14.58 (8.40, 21.20)	5.00 (-1.60, 9.30)	1.39 (2.05 - 0.94)	0.095	0.19
**iNKT CD8+**	GranzB	-1.60 (-6.20, -0.70)	-4.10 (-5.90, 9.10)	0.68 (1.19 - 0.39)	0.17	0.31
**iNKT CD8+**	IFNγ	0.47 (0.24, 1.74)	0.94 (-0.24, 2.80)	0.87 (1.25 - 0.60)	0.44	0.58

**iNKT CD8+**	IL17	-0.11 (-0.64, 0.12)	0.13 (-0.10, 0.55)	0.88 (1.24 - 0.62)	0.46	0.60
**iNKT CD8+**	Ki67	-0.15 (-0.64, 0.11)	0.68 (0.37, 1.07)	0.84 (0.98 - 0.72)	0.028	0.098
**iNKT CD8+**	PD1	1.90 (1.10, 5.30)	1.90 (-0.30, 9.60)	0.89 (1.10 - 0.71)	0.27	0.42
**MAIT MR1-**	CD25	1.19 (-1.22, 1.78)	2.38 (1.11, 2.97)	0.80 (0.99 - 0.64)	0.036	0.12
**MAIT MR1-**	CD69	0.40 (-0.20, 5.10)	1.40 (-2.90, 5.10)	0.88 (1.18 - 0.65)	0.38	0.51
**MAIT MR1-**	GranzB	-0.17 (-0.47, 0.30)	0.56 (-0.07, 1.30)	1.45 (1.99 - 1.05)	0.023	0.086
**MAIT MR1-**	IL10	0.11 (-0.17, 0.22)	0.13 (0.02, 0.77)	0.82 (0.99 - 0.68)	0.038	0.12
**MAIT MR1-**	PD1	1.42 (0.90, 1.80)	1.10 (0.16, 2.12)	1.07 (1.21 - 0.94)	0.30	0.44
**MAIT MR1+**	CD25	2.38 (0.98, 3.25)	2.23 (1.65, 8.62)	0.59 (0.87 - 0.40)	0.0075	0.039
**MAIT MR1+**	CD69	12.60 (9.40, 15.10)	2.40 (0.50, 7.70)	0.99 (1.39 - 0.71)	0.98	0.98
**MAIT MR1+**	GranzB	0.41 (0.08, 0.62)	1.23 (0.31, 4.42)	0.59 (0.85 - 0.41)	0.0044	0.029
**MAIT MR1+**	IFNγ	0.78 (0.65, 2.25)	0.69 (0.47, 7.31)	0.62 (1.04 - 0.37)	0.07	0.15
**MAIT MR1+**	IL10	0.11 (-0.09, 0.32)	0.09 (0.02, 0.88)	0.84 (1.17 - 0.61)	0.31	0.44
**MAIT MR1+**	PD1	0.57 (0.23, 0.99)	0.68 (-0.20, 0.81)	1.27 (1.48 - 1.10)	0.0013	0.01
**NK**	CD107a	3.24 (1.38, 3.46)	1.37 (1.22, 3.16)	1.14 (1.45 - 0.90)	0.27	0.42
**NK**	CD25	8.87 (7.39, 15.10)	2.97 (2.18, 4.12)	1.94 (2.68 - 1.41)	5.2e-05	0.00055
**NK**	CD69	41.10 (30.00, 50.00)	14.10 (-3.50, 21.50)	2.74 (4.15 - 1.80)	2.2e-06	4.6e-05
**NK**	GranzB	7.20 (5.40, 14.30)	1.60 (-1.60, 4.80)	1.31 (1.70 - 1.00)	0.049	0.13
**NK**	IFNγ	3.74 (1.87, 5.55)	1.29 (0.85, 3.90)	1.37 (2.08 - 0.90)	0.14	0.25
**NK**	Ki67	-0.24 (-0.50, 0.09)	0.10 (-0.11, 0.16)	0.99 (1.05 - 0.93)	0.78	0.85
**NK**	PD1	2.52 (1.97, 3.58)	1.27 (0.45, 1.76)	1.24 (1.60 - 0.96)	0.098	0.19
**NKT CD4+**	CD107a	3.72 (2.70, 7.70)	6.20 (4.98, 9.00)	0.87 (1.11 - 0.68)	0.26	0.41
**NKT CD4+**	CD25	7.89 (6.37, 12.50)	7.40 (2.60, 14.90)	1.07 (1.45 - 0.79)	0.66	0.77
**NKT CD4+**	CD69	13.30 (10.70, 24.90)	11.80 (8.50, 13.00)	1.26 (1.63 - 0.98)	0.07	0.15
**NKT CD4+**	GranzB	5.70 (0.90, 7.50)	-2.40 (-7.80, -0.80)	1.14 (1.41 - 0.92)	0.22	0.37
**NKT CD4+**	IFNγ	1.93 (1.26, 3.79)	1.82 (0.42, 3.53)	1.07 (1.52 - 0.76)	0.69	0.79
**NKT CD4+**	IL10	0.42 (-0.04, 1.09)	0.14 (0.04, 0.31)	1.26 (1.53 - 1.04)	0.017	0.068
**NKT CD4+**	Ki67	-0.37 (-0.88, 0.63)	-0.09 (-0.48, 0.28)	1.16 (1.41 - 0.96)	0.13	0.24
**NKT CD4+**	PD1	2.10 (-1.50, 4.60)	1.20 (-0.10, 2.90)	1.07 (1.32 - 0.87)	0.50	0.63
**NKT CD8+**	CD107a	-0.47 (-1.90, -0.09)	9.90 (7.20, 12.85)	0.34 (0.50 - 0.22)	1.3e-07	3.3e-06
**NKT CD8+**	CD25	1.60 (0.48, 7.07)	7.17 (4.17, 10.76)	0.54 (0.83 - 0.35)	0.0047	0.029
**NKT CD8+**	CD69	15.40 (6.90, 18.40)	0.70 (-0.60, 14.80)	1.60 (2.25 - 1.13)	0.0081	0.039
**NKT CD8+**	GranzB	7.50 (-0.60, 12.10)	-7.40 (-10.30, -4.80)	2.08 (2.90 - 1.50)	1.3e-05	0.00017
**NKT CD8+**	IFNγ	0.50 (0.16, 2.08)	0.06 (-0.09, 0.91)	1.35 (1.87 - 0.97)	0.079	0.16
**NKT CD8+**	IL10	-0.34 (-1.87, 0.54)	1.45 (0.66, 2.88)	0.83 (1.12 - 0.61)	0.22	0.37
**NKT CD8+**	IL2	0.10 (-0.49, 0.36)	1.09 (0.31, 2.50)	0.74 (1.07 - 0.51)	0.11	0.21
**NKT CD8+**	Ki67	-0.37 (-0.64, -0.09)	0.04 (-0.45, 0.42)	0.99 (1.17 - 0.84)	0.91	0.95
**NKT CD8+**	PD1	0.90 (-1.76, 1.50)	-0.28 (-0.40, 1.81)	0.94 (1.25 - 0.70)	0.66	0.77
**Tconv CD4+**	CD107a	0.16 (0.07, 0.30)	0.49 (0.30, 0.53)	0.87 (0.97 - 0.78)	0.01	0.044
**Tconv CD4+**	CD25	4.30 (2.70, 7.70)	7.40 (4.77, 10.97)	0.78 (1.00 - 0.62)	0.046	0.13
**Tconv CD4+**	CD69	4.20 (3.50, 7.90)	2.10 (0.10, 8.30)	0.78 (0.99 - 0.61)	0.044	0.13
**Tconv CD4+**	PD1	1.37 (0.98, 1.70)	1.90 (1.60, 2.90)	0.94 (1.02 - 0.87)	0.13	0.25
**Tconv CD8+**	CD25	0.51 (-0.04, 1.04)	1.73 (0.89, 2.79)	0.72 (0.86 - 0.61)	0.00033	0.0032
**Tconv CD8+**	CD69	1.10 (-0.80, 3.30)	1.20 (-2.40, 3.80)	0.81 (1.00 - 0.65)	0.05	0.13
**Tconv CD8+**	GranzB	0.06 (-1.21, 0.90)	1.33 (1.00, 2.00)	0.86 (1.01 - 0.73)	0.064	0.15
**Tconv CD8+**	PD1	0.47 (0.10, 1.40)	1.30 (0.91, 1.80)	0.90 (1.00 - 0.81)	0.053	0.13
**γδT**	CD107a	0.69 (0.43, 1.15)	2.28 (0.85, 4.50)	0.85 (1.10 - 0.65)	0.21	0.36
**γδT**	CD25	1.71 (1.29, 2.85)	2.84 (2.17, 4.70)	0.94 (1.20 - 0.73)	0.60	0.72
**γδT**	CD69	24.00 (16.30, 29.40)	4.10 (0.20, 17.10)	0.76 (1.31 - 0.44)	0.32	0.44
**γδT**	GranzB	1.80 (0.60, 3.10)	2.90 (0.20, 4.50)	0.98 (1.11 - 0.86)	0.73	0.82
**γδT**	IFNγ	1.00 (0.64, 1.64)	3.62 (1.93, 6.10)	0.72 (1.01 - 0.52)	0.057	0.14
**γδT**	Ki67	0.07 (-0.15, 0.24)	0.18 (0.05, 0.32)	0.99 (1.05 - 0.93)	0.77	0.84
**γδT**	PD1	2.40 (1.30, 3.90)	2.30 (1.68, 4.30)	1.11 (1.23 - 1.00)	0.06	0.14

* Median (quartiles)
** Point estimate (95% Confidence interval

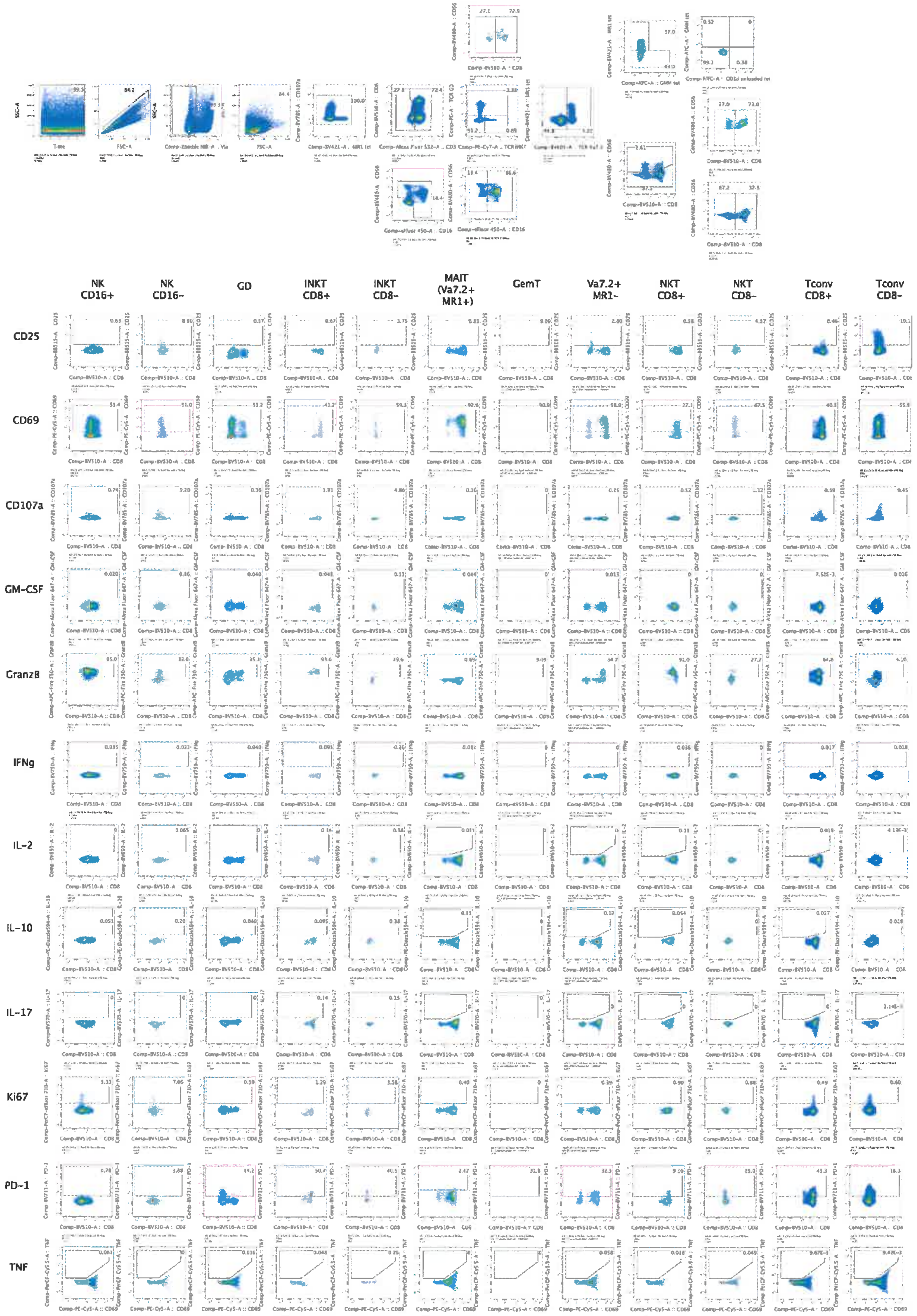

Figure S1. Gating strategy TB-resister unstimulated T cells; related to Fig 1, 2, 3, S2, S3, S5, and S6

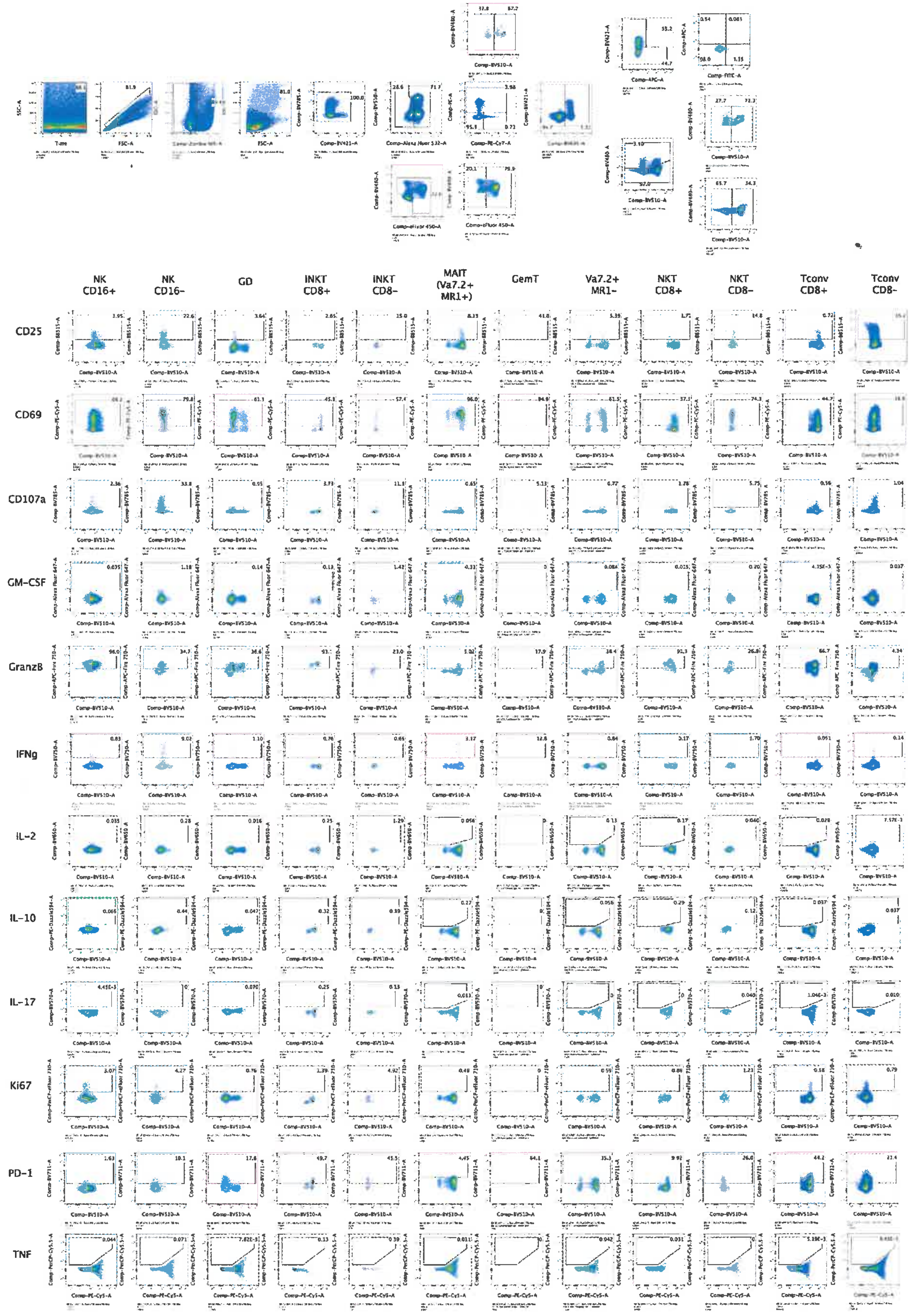

Figure S2. Gating strategy TB-resister *Mtb*-stimulated T cells; related to Fig 1, 2, 3, S2, S3, S5, and S6

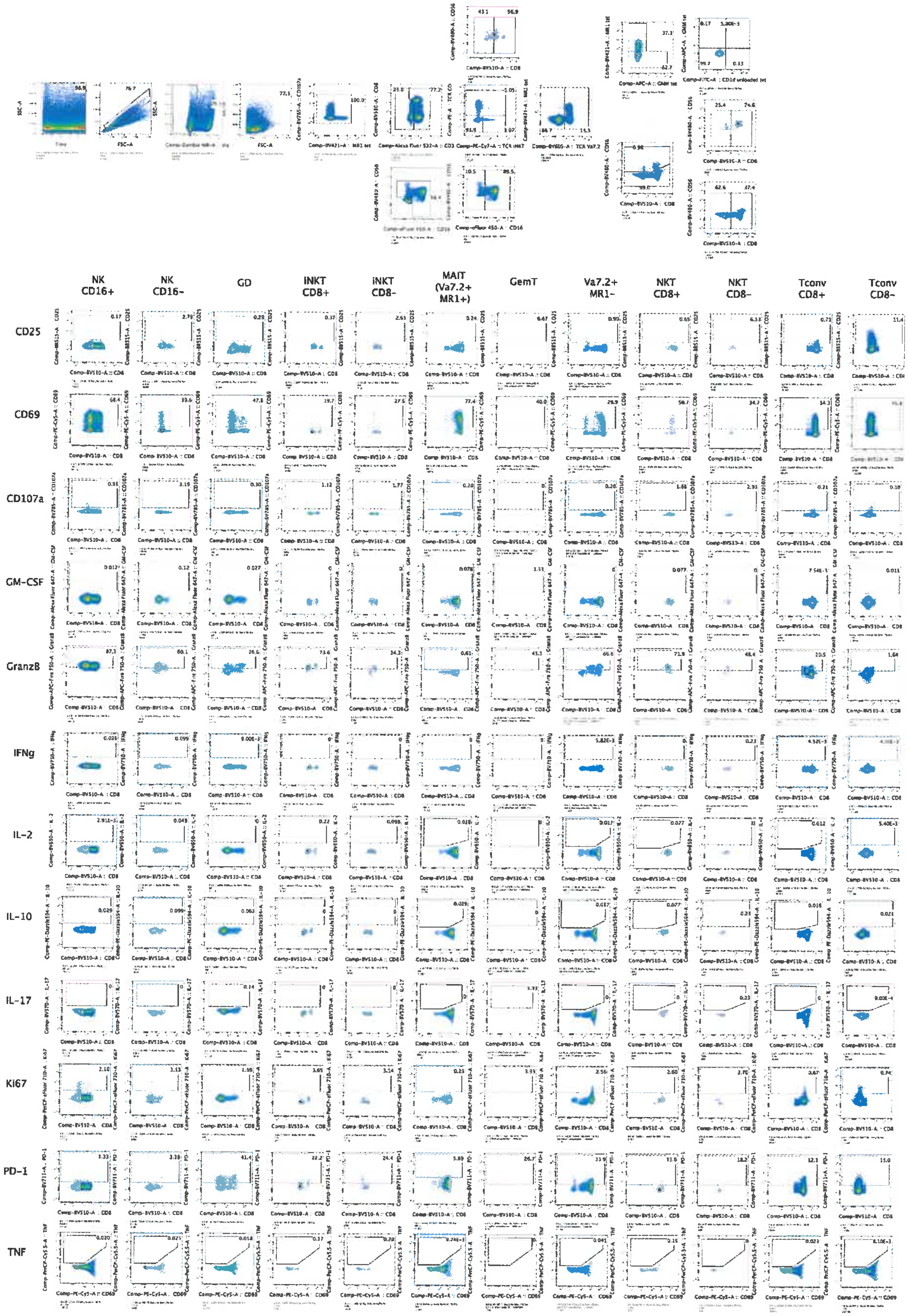

Figure S3. Gating strategy LTBI unstimulated T cells; related to Fig 1, 2, 3, S2, S3, S5, and S6

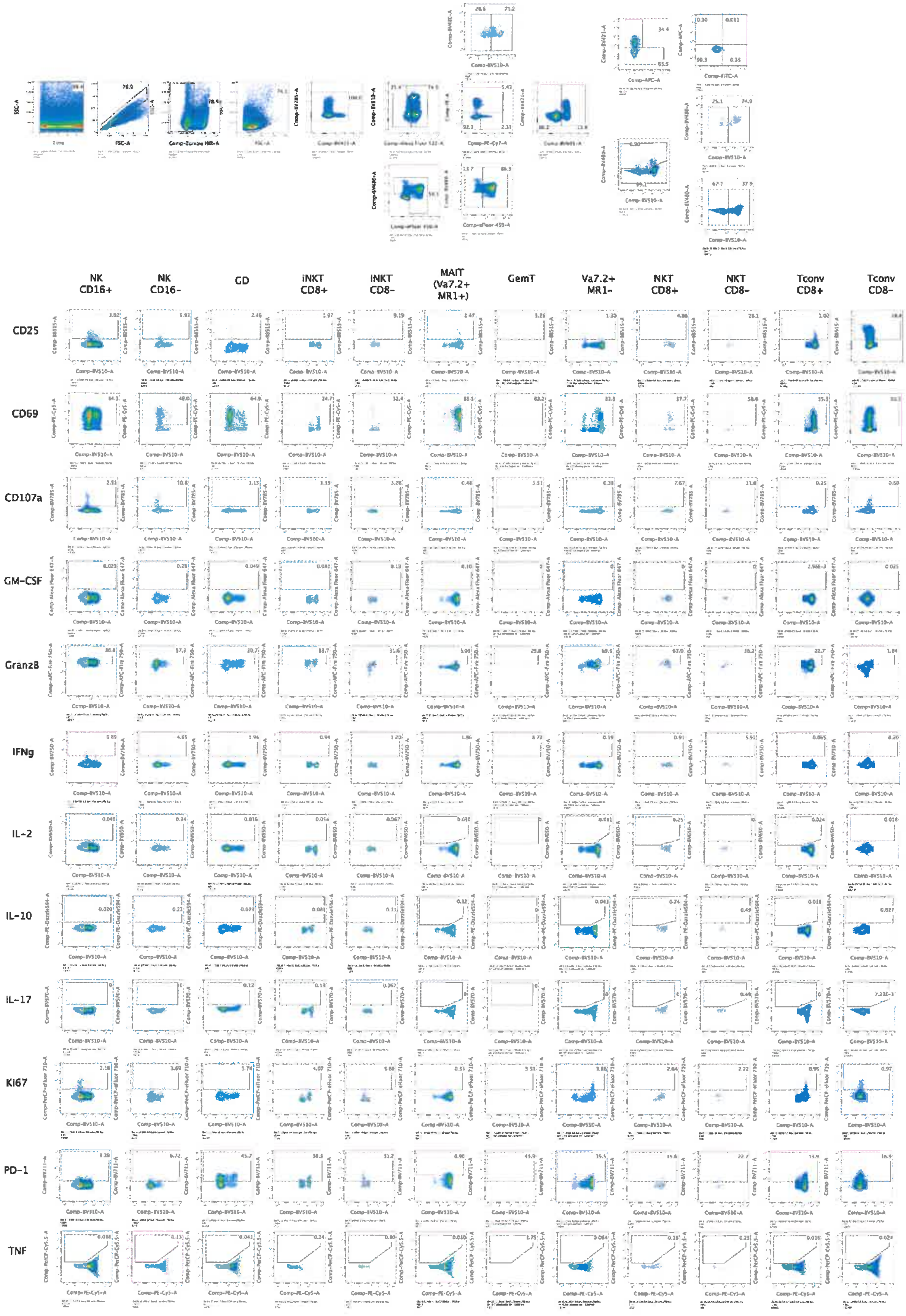

Figure S4. Gating strategy LTBI *Mtb*-stimulated T cells; related to Fig 1, 2, 3, S2, S3, S5, and S6

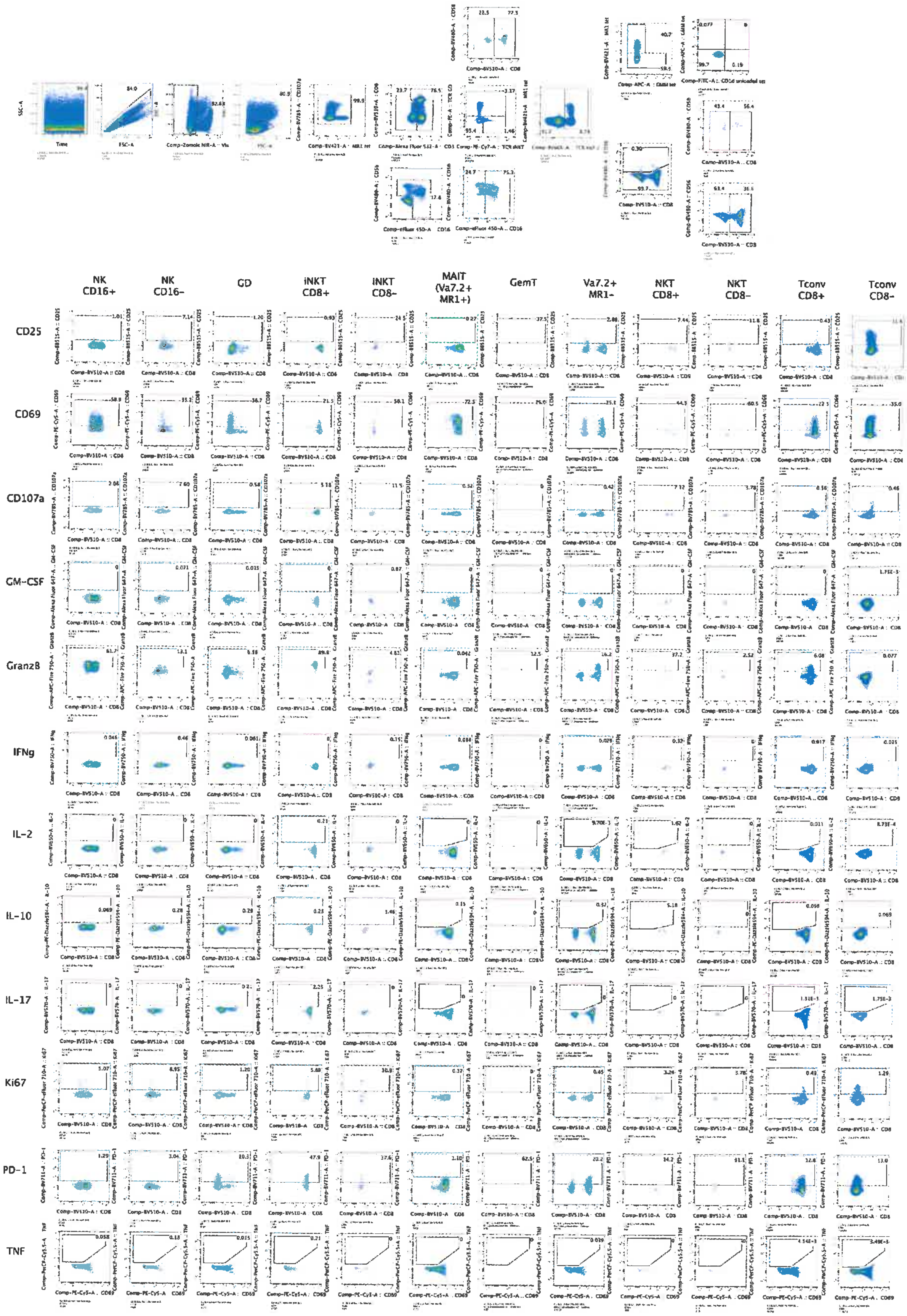

Figure S5. Gating strategy BCG unstimulated T cells; related to Fig 1, 2, 3, S2, S3, S5, and S6

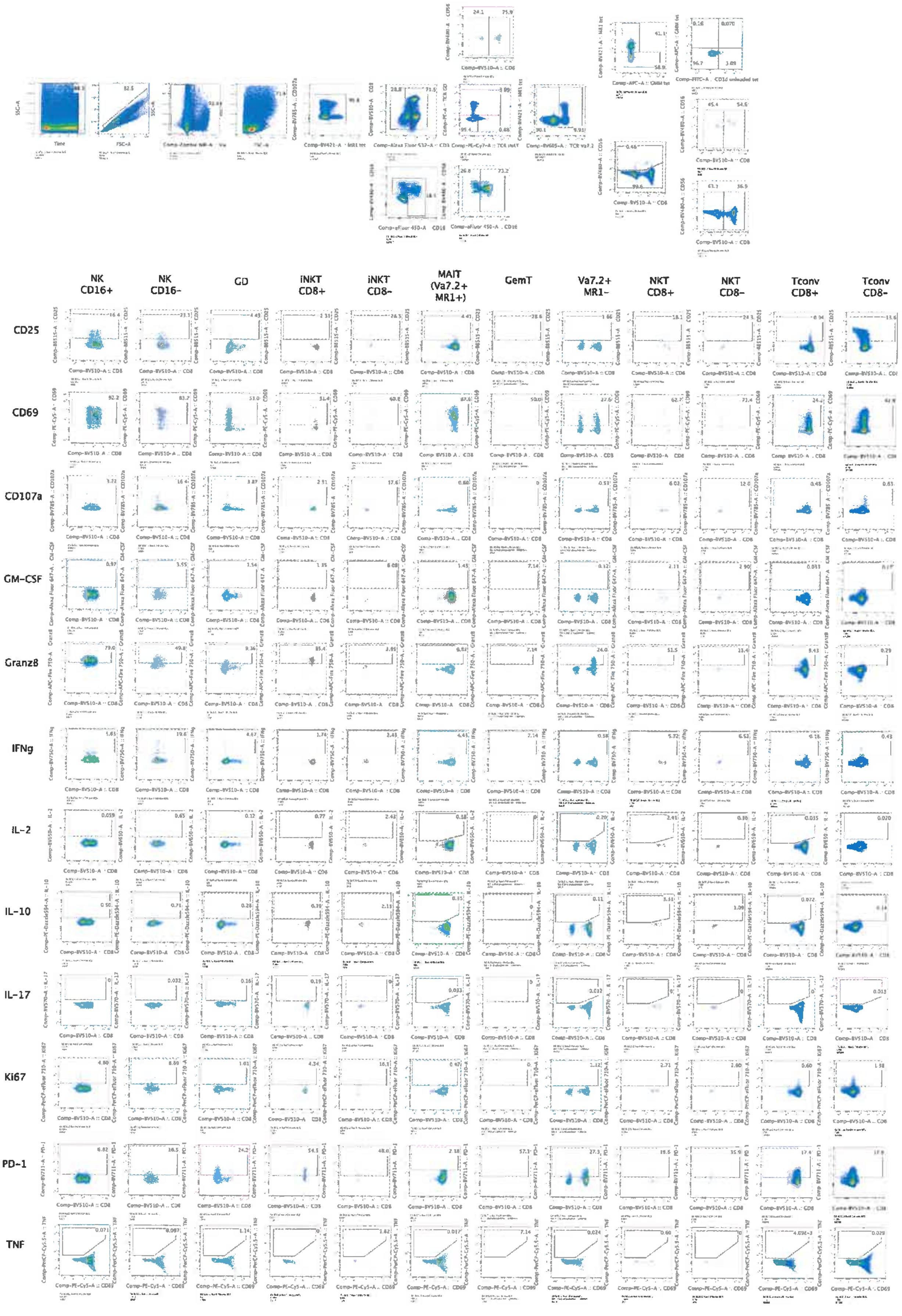

Figure S6. Gating strategy BCG *Mtb*-stimulated T cells; related to Fig 1, 2, 3, S2, S3, S5, and S6

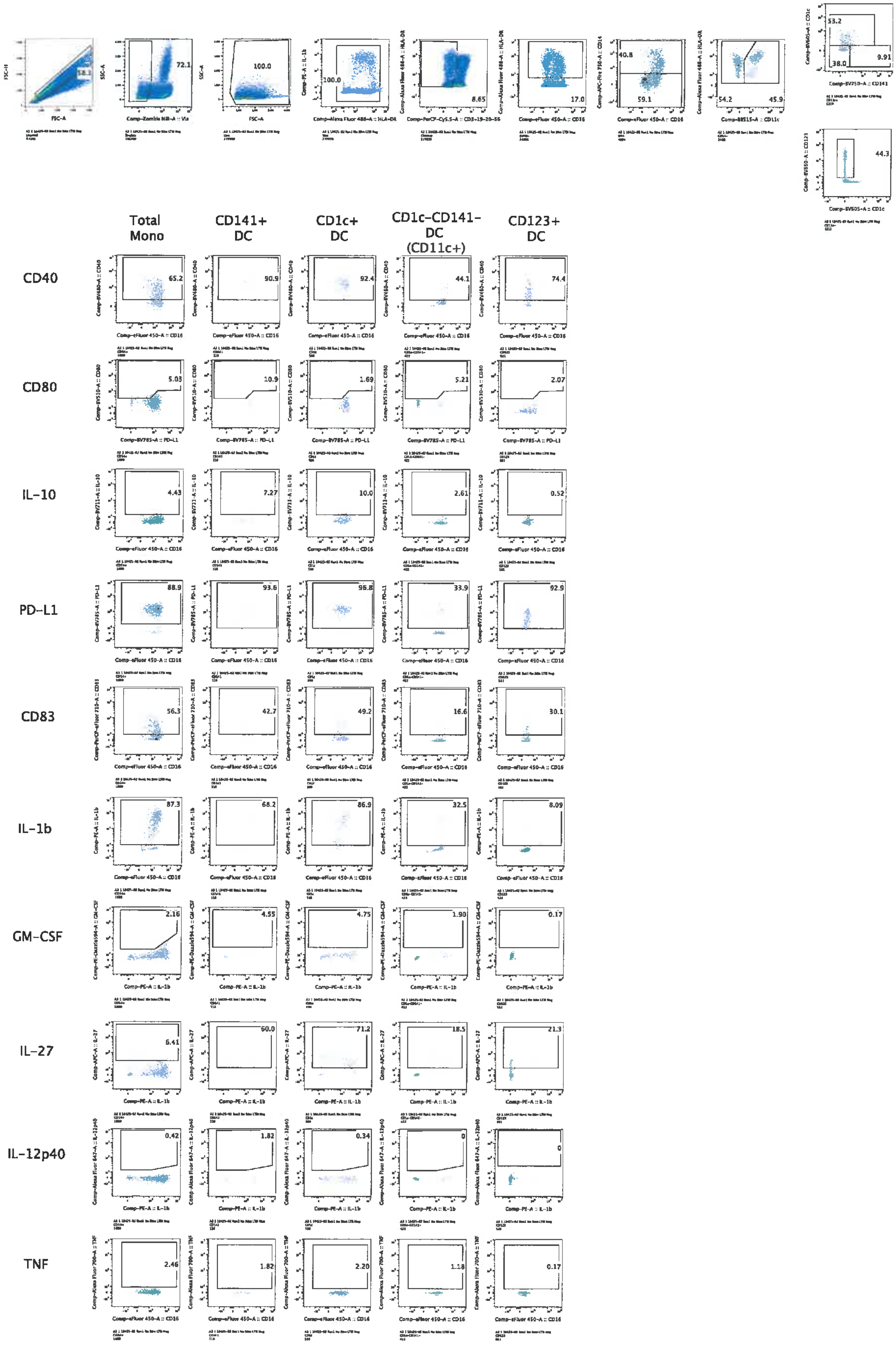

Figure S7. Gating strategy TB-resister unstimulated APC; related to Fig 1, 2, 3, S2, S3, S5, and S6

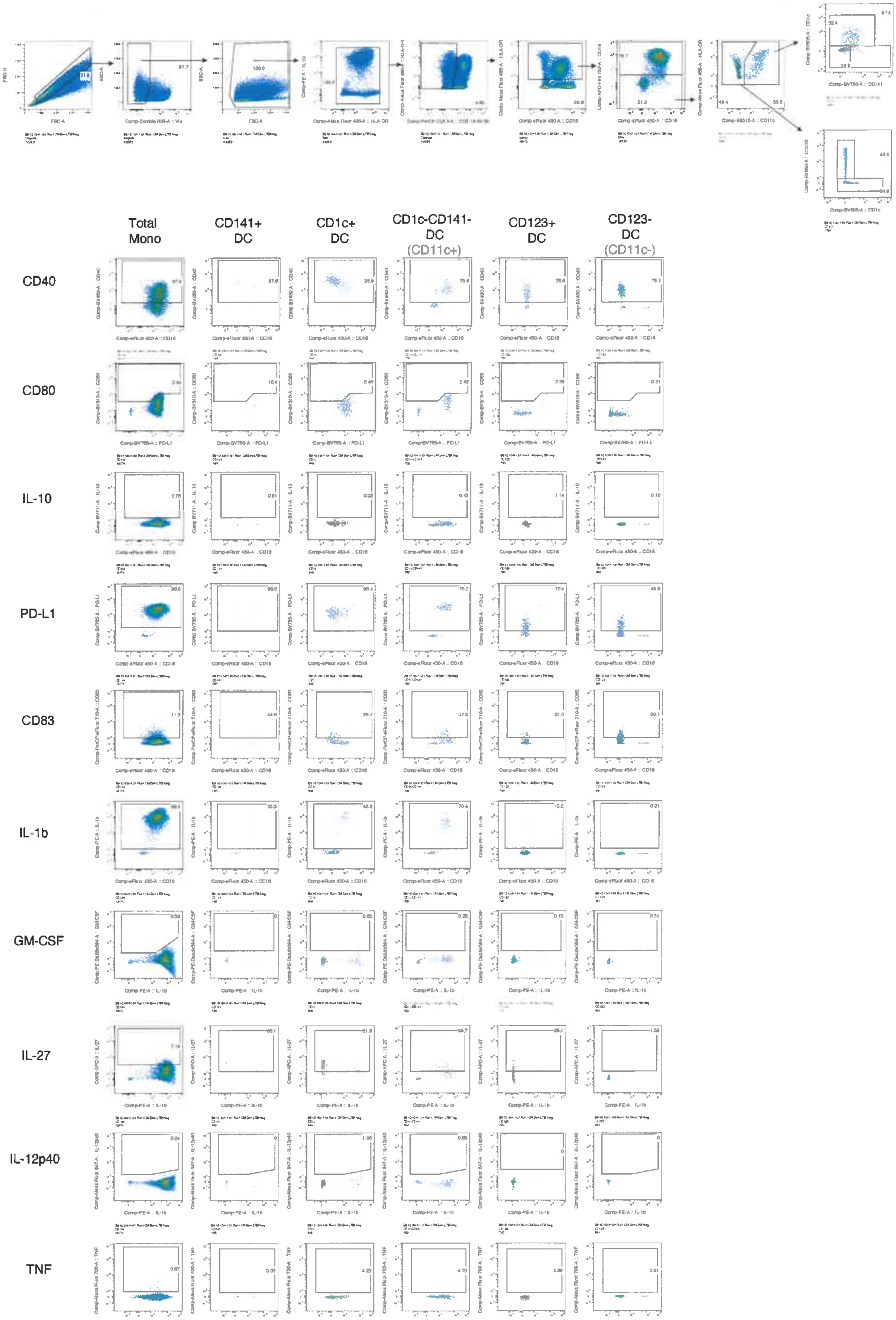

Figure S8. Gating strategy TB-resister *Mtb*-stimulated APC; related to Fig 1, 2, 3, S2, S3, S5, and S6

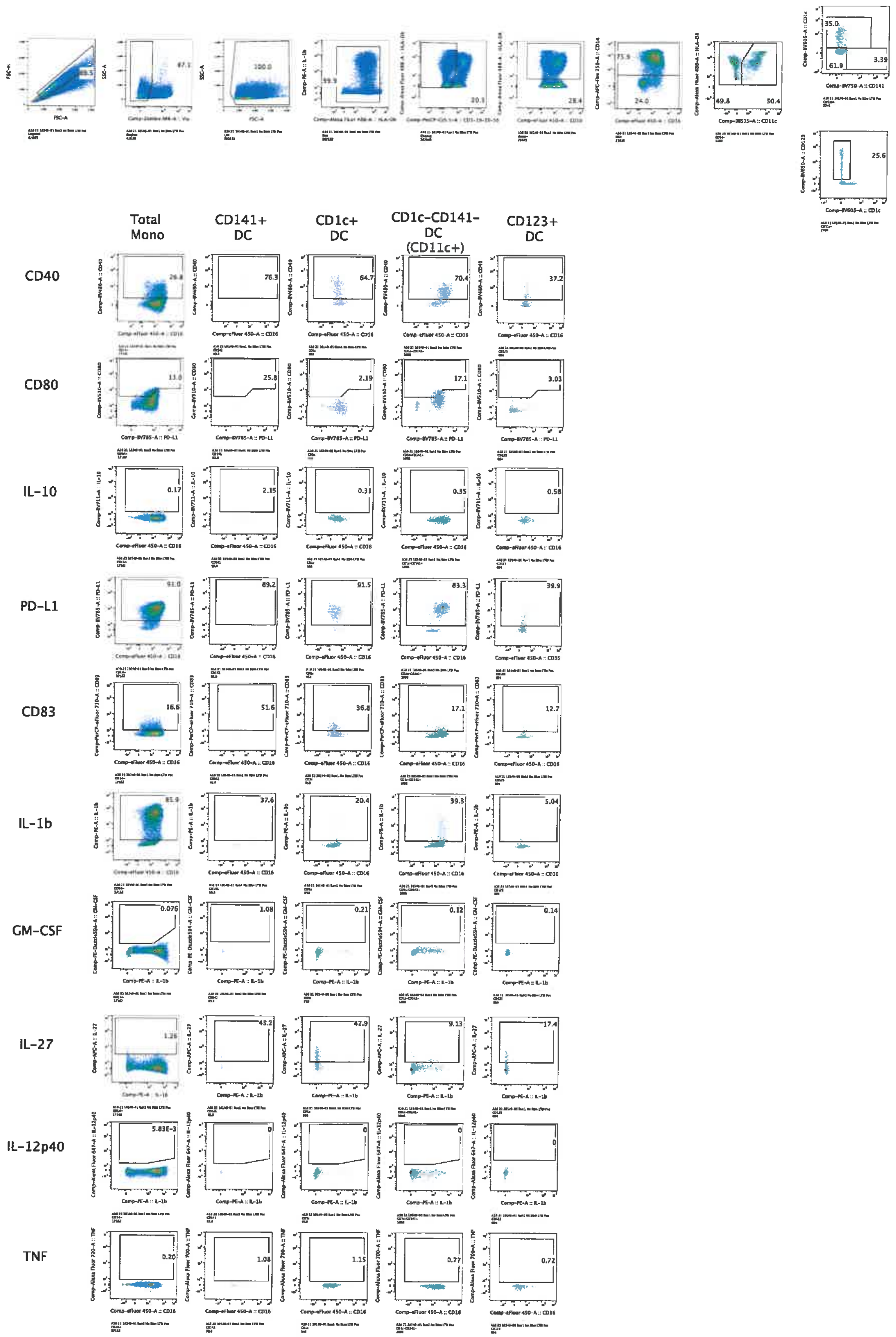

Figure S9. Gating strategy LTBI-participant unstimulated APC; related to Fig 1, 2, 3, S2, S3, S5, and S6

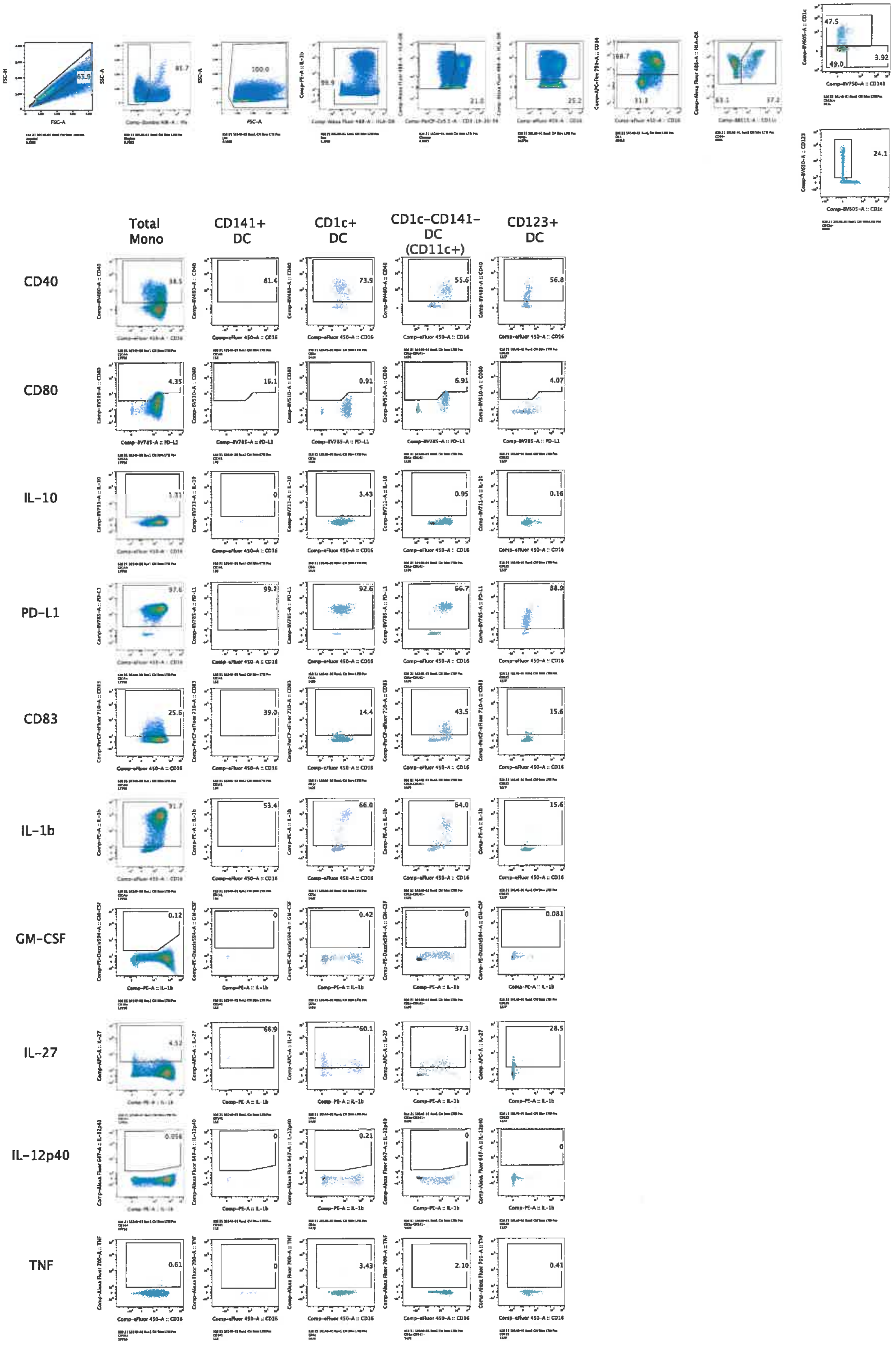

Figure S10. Gating strategy LTBI *Mtb*-stimulated APC; related to Fig 1, 2, 3, S2, S3, S5, and S6

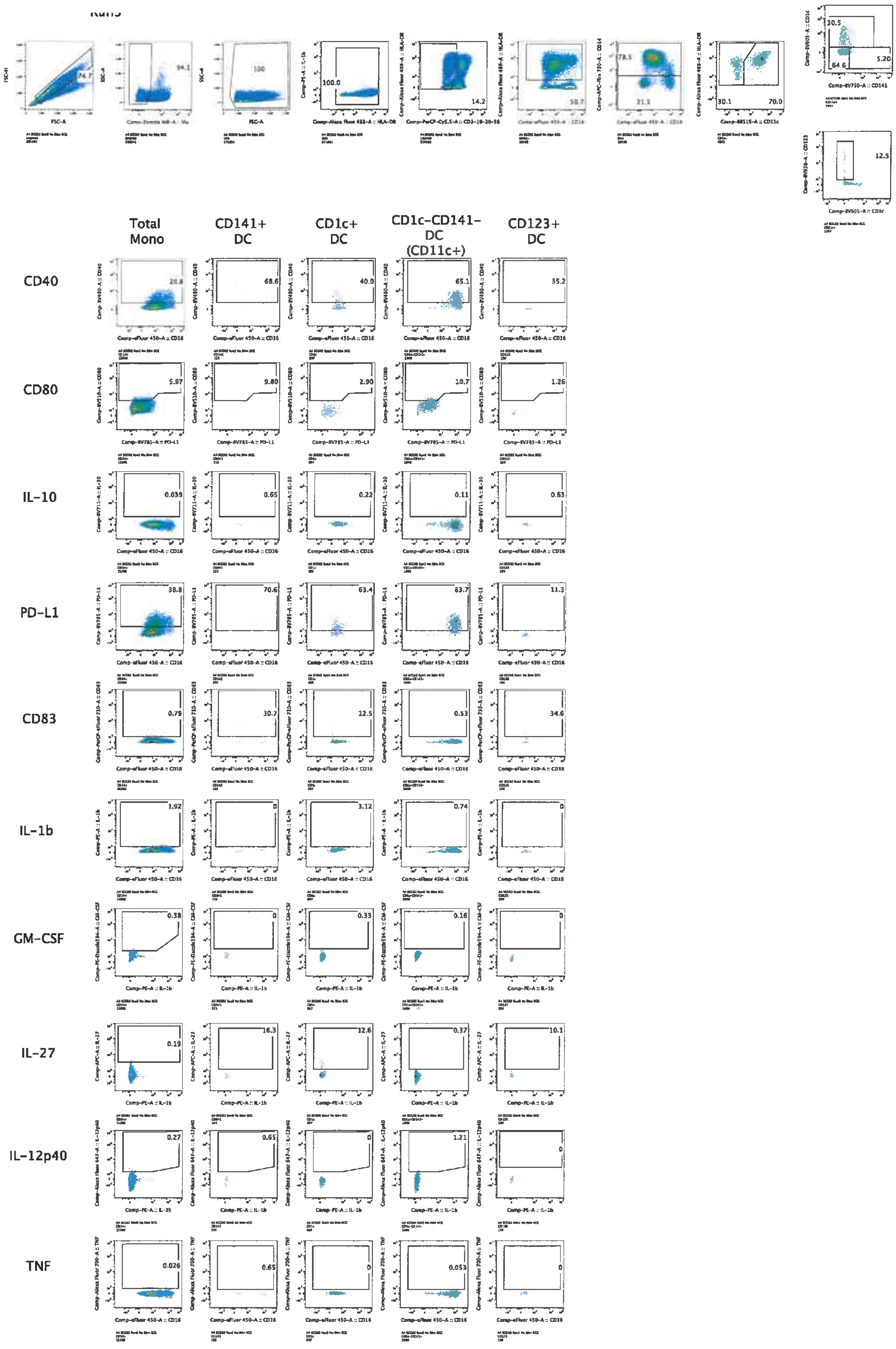

Figure S11. Gating strategy BCG-recipient unstimulated APC; related to Fig 1, 2, 3, S2, S3, S5, and S6

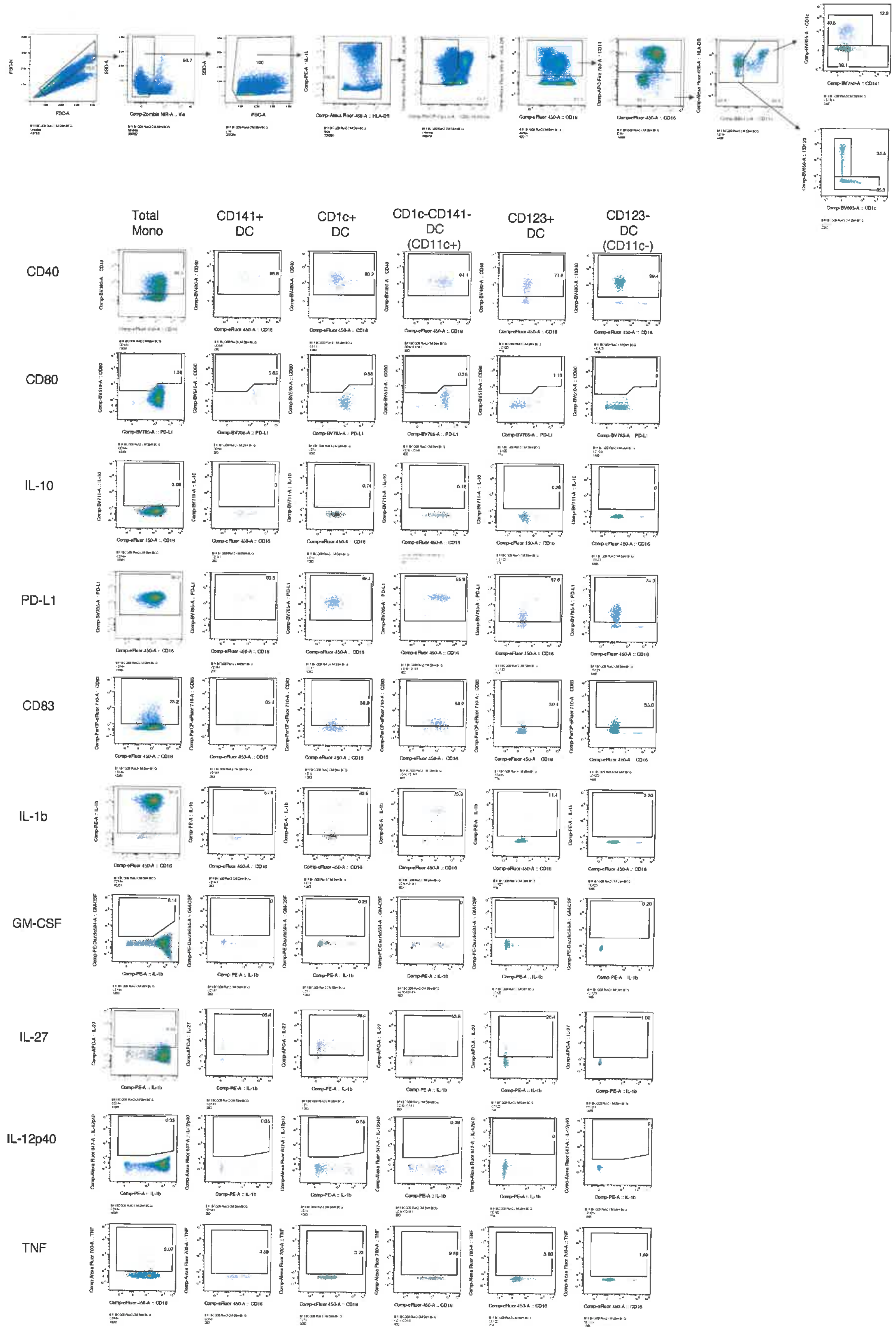

Figure S12. Gating strategy BCG-recipient *Mtb*-stimulated APC; related to Fig 1, 2, 3, S2, S3, S5, and S6

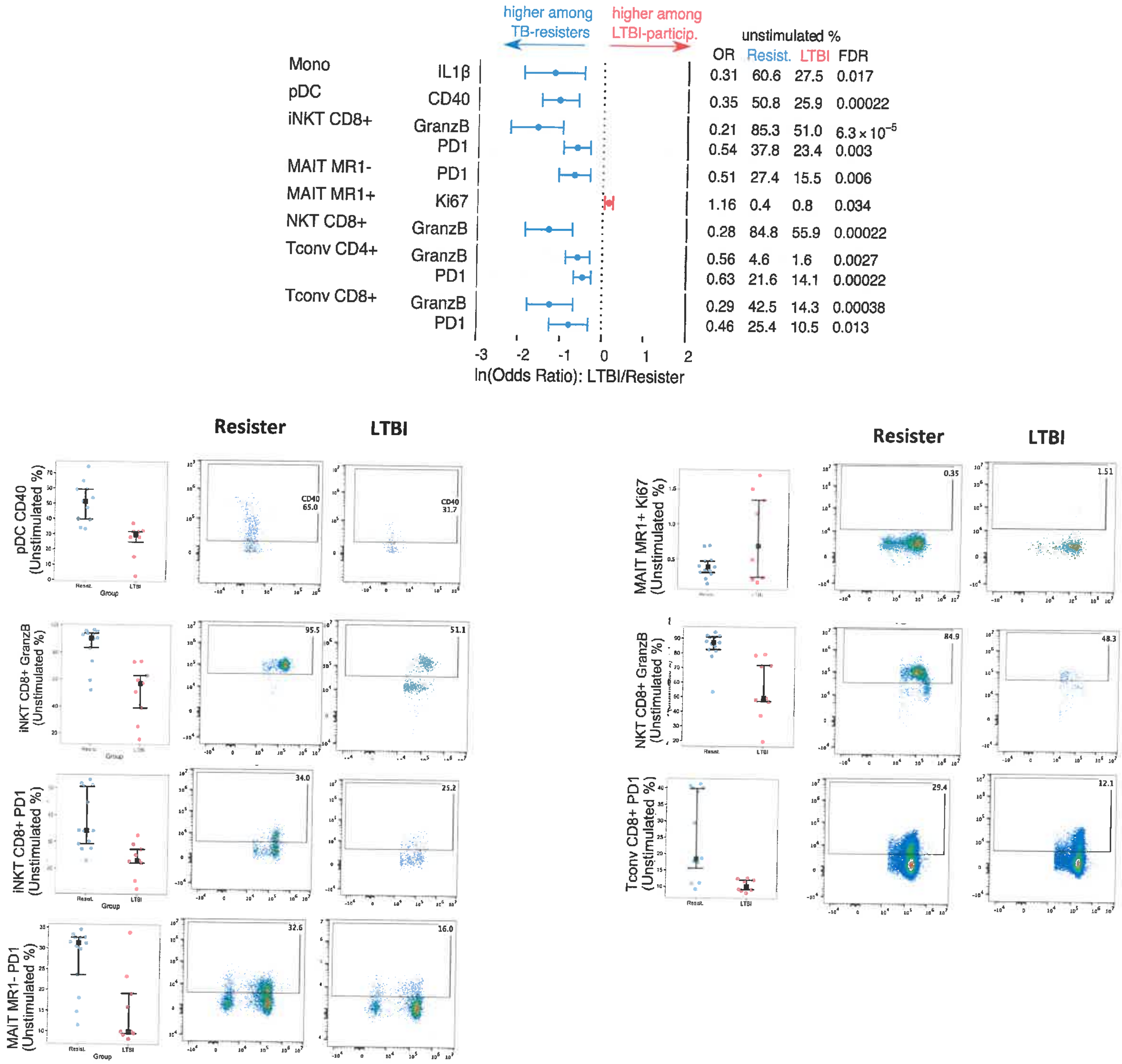

Fig S13. Functional phenotypes differentially expressed in unstimulated PBMC from TB-resisters and LTBI-participants related to Fig 1. This figure is a continuation of Fig 1. **Top:** Forest plot displays ln-transformed odds ratios (lnOR) and 95% confidence intervals (CI). The dotted line shows no average effect (lnOR = 0, corresponding to OR = 1). Features on the right side of this line are more highly expressed among LTBI-participants (OR > 1), and features on the left side are more highly expressed among TB-resisters (OR < 1). The table shows the absolute OR and the means of each parameter in TB-resisters and LTBI-participants. **Bottom** Typical scatter plots exemplifying differences in CD40+ pDC, CD8+GranzB+ iNKT, CD8+PD1+ iNKT, MR1-PD1+ MAIT, MR1+Ki67+ MAIT, CD8+GranzB+ NKT, CD8+PD1+ Tconv and typical dot plot examples, in which the ordinate shows the expression of the activation marker depicted in the corresponding scatter plot and the abscissa shows the expression of CD8.

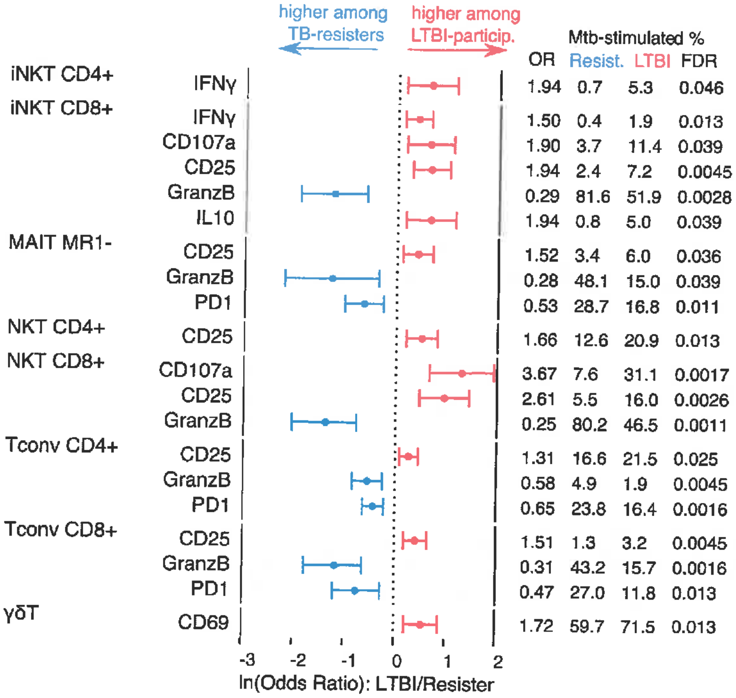

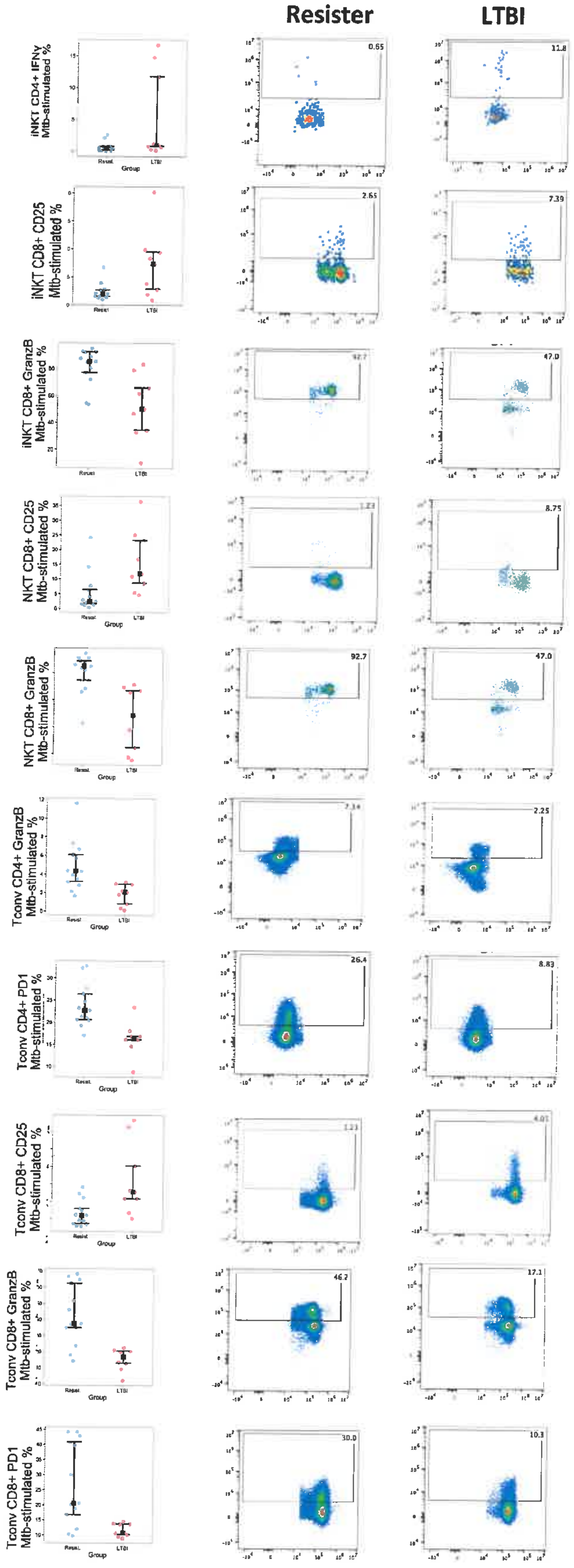

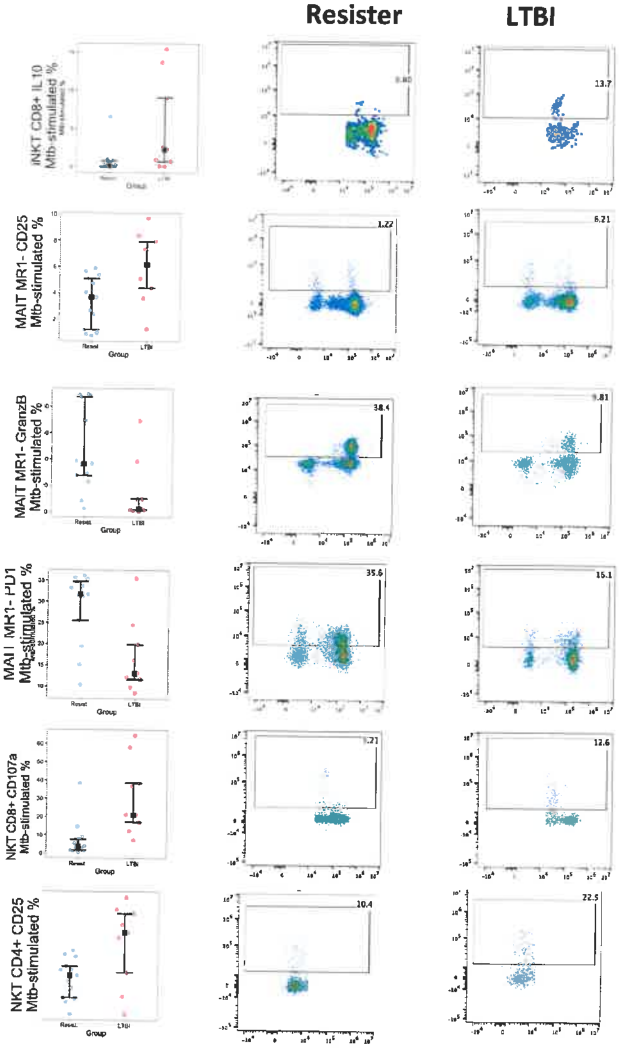

Figure S14. Functional phenotypes differentially expressed in *Mtb*-stimulated PBMC from TB-resisters and LTBI-participants related to Fig 2. This is a continuation of **Fig 2**. PBMC were analyzed after pre-optimized overnight stimulation with *Mtb* cell membrane (see methods). **Top** Forest plot displays natural log (ln)-transformed odds ratios (lnOR) and 95% confidence intervals (CI). The dotted line shows no average effect (lnOR = 0, corresponding to OR = 1). Features on the right side of this line are more highly expressed among LTBI-participants (OR > 1), and features on the left side are more highly expressed among TB-resisters (OR < 1). The table shows the absolute OR and the means of each parameter in TB-resisters and LTBI-participants. **Bottom** Scatter plots exemplifying significant differences and typical dot plot examples, in which the ordinate shows the expression of the activation marker depicted in the companion scatter plot and the abscissa shows the expression of CD8.

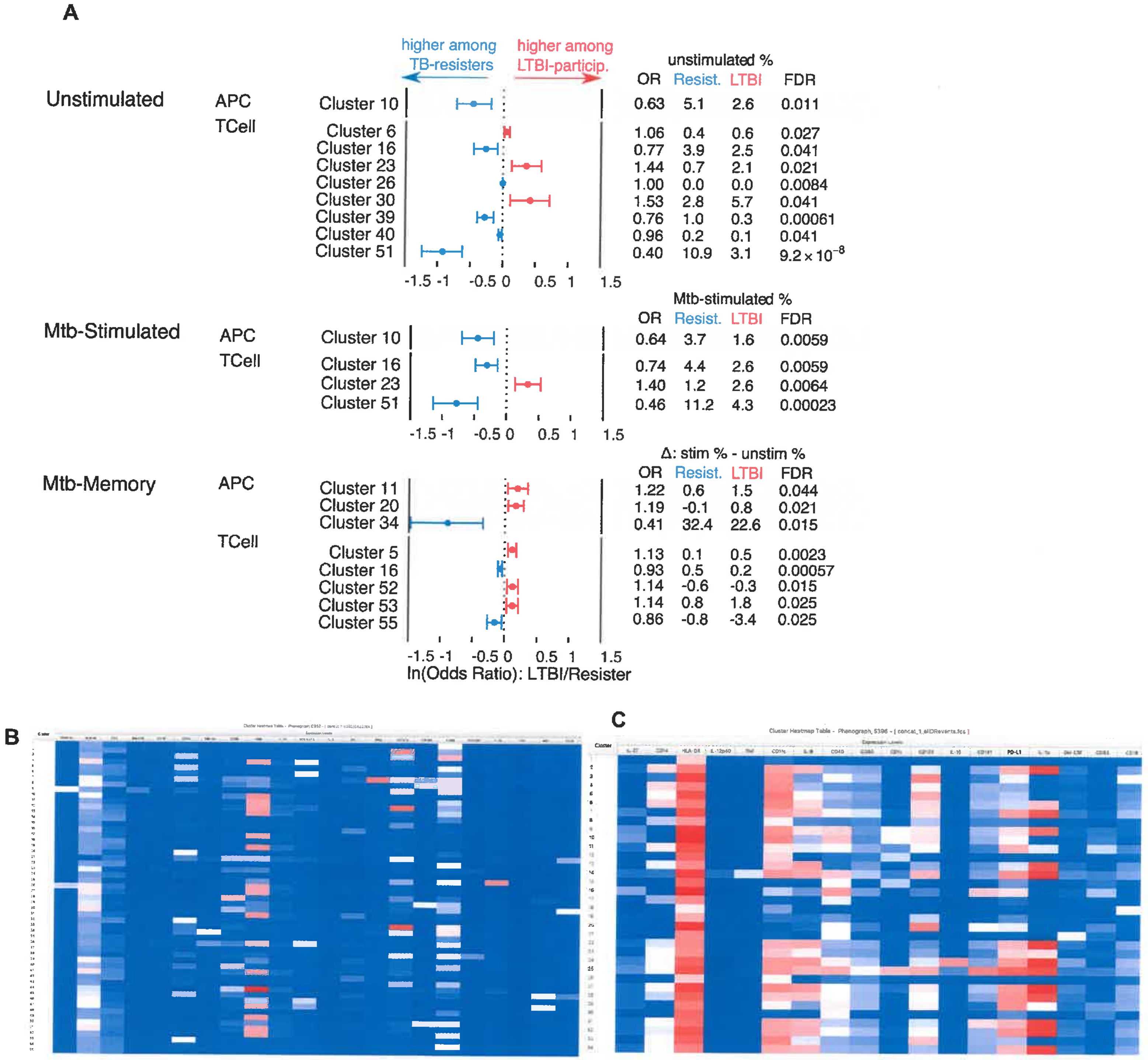

Figure S15. Cluster analysis of adaptive and innate immune responses in TB-resisters and LTBI-participants; related to Fig 5. Data were derived from 13 TB-resisters and 9 LTBI-participants for the T cell clustering and 12 TB-resisters and 8 LTBI-participants for the APC clustering. **A**: Forest trees of lnOR and 95% CI, means, and FDR-corrected p values of clusters significantly different between the two groups. **4B&C. Cluster analysis of adaptive and innate immune responses in TB-resisters and LTBI-participants; related to Fig 5. B**: Heat map of T-cell clusters identified by Phenograph. **C**: Heat map of APC clusters.

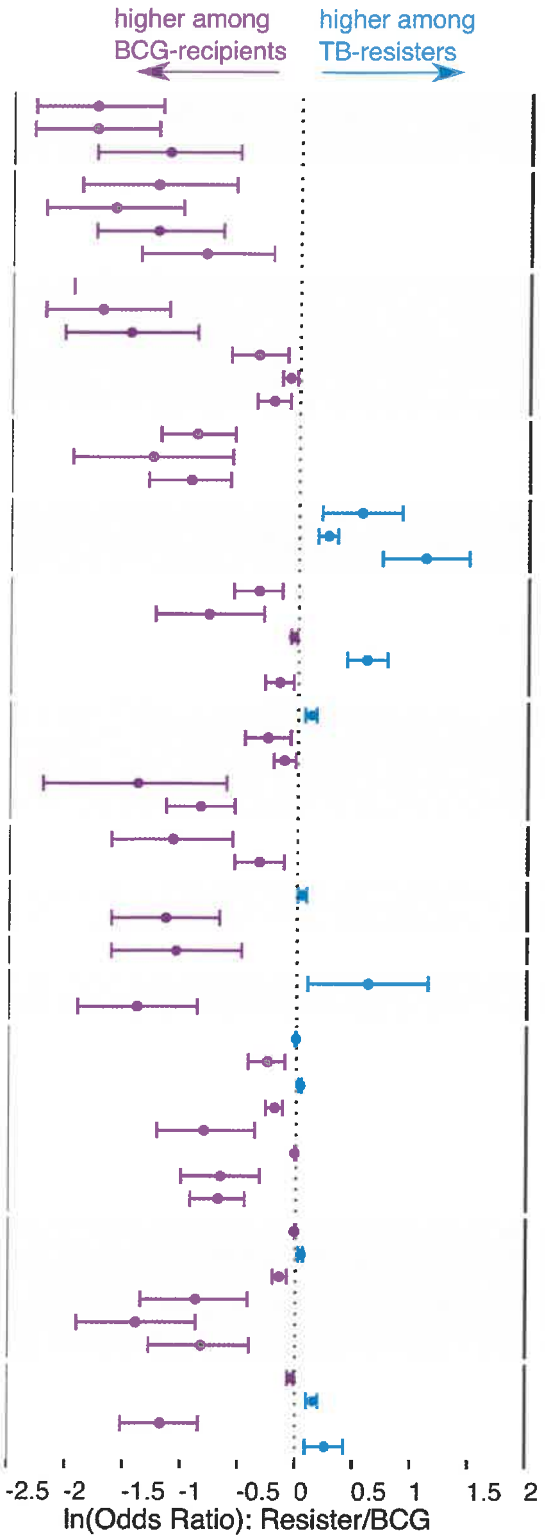

Subset	Feature	OR	unstimulated % BCG	unstimulated % Resister	FDR
cDC1	IL1β	0.17	23.2	1.9	7.1×10^{-9}
	IL8	0.17	44.1	10.0	2.1×10^{-9}
	PDL1	0.32	83.9	56.7	0.0013
cDC2	IL1β	0.29	23.1	3.0	0.001
	IL8	0.20	47.3	13.5	8.4×10^{-7}
	PDL1	0.29	85.6	60.2	7×10^{-5}
	CD40	0.44	72.8	56.0	0.017
Mono	IL1β	0.08	60.6	8.1	1.3×10^{-13}
	IL8	0.18	82.7	42.8	2.6×10^{-8}
	PDL1	0.23	75.9	40.7	4.3×10^{-6}
	CD80	0.70	9.4	6.0	0.012
	IL12p40	0.92	0.2	0.1	0.025
	TNFα	0.80	0.6	0.0	0.007
pDC	IL8	0.41	6.1	1.5	3.3×10^{-7}
	PDL1	0.28	34.4	9.1	0.0013
	CD40	0.39	50.8	29.1	8.4×10^{-7}
iNKT CD4+	CD25	1.74	6.0	11.6	0.0054
	IL10	1.30	0.1	0.7	2.6×10^{-8}
	Ki67	3.03	4.3	16.6	1.1×10^{-7}
iNKT CD8+	CD107a	0.71	2.7	1.3	0.0035
	CD69	0.46	34.7	18.2	0.0044
	GMCSF	0.96	0.1	0.0	0.012
	IL17	1.82	0.3	2.1	2.7×10^{-10}
	IL2	0.85	0.5	0.1	0.022
MAIT MR1-	IL10	1.13	0.1	0.4	5.8×10^{-6}
	Ki67	0.77	1.3	0.5	0.025
	CD107a	0.89	0.5	0.2	0.034
	GranzB	0.25	46.7	9.4	0.0016
	PD1	0.43	27.4	12.7	3.7×10^{-7}
MAIT MR1+	CD69	0.34	87.3	68.8	0.00032
	PD1	0.72	2.5	1.2	0.0095
NK	IL10	1.05	0.1	0.2	0.04
	CD69	0.32	66.2	38.6	1.2×10^{-5}
NKT CD4+	GranzB	0.35	47.8	23.8	0.0012
NKT CD8+	IL10	1.87	2.1	5.2	0.047
	GranzB	0.25	84.8	53.5	1×10^{-6}
Tconv CD4+	TNFα	1.00	0.0	0.0	0.0066
	CD25	0.78	12.7	9.9	0.0054
	IL10	1.04	0.0	0.1	1×10^{-10}
	CD107a	0.83	0.7	0.3	1.6×10^{-6}
	CD69	0.45	53.2	33.8	0.0012
	GMCSF	0.99	0.0	0.0	0.00097
	GranzB	0.52	4.6	1.6	0.00083
	PD1	0.51	21.6	11.6	1.2×10^{-7}
Tconv CD8+	TNFα	0.99	0.0	0.0	0.00043
	IL10	1.05	0.0	0.1	1.6×10^{-7}
	CD107a	0.87	0.6	0.2	8.5×10^{-5}
	CD69	0.42	45.3	24.9	0.001
	GranzB	0.25	42.5	12.9	1.2×10^{-6}
	PD1	0.44	25.4	10.9	0.00083
γδT	TNFα	0.97	0.1	0.0	0.047
	IL10	1.17	0.1	0.4	7×10^{-10}
	CD69	0.31	55.4	27.3	2.2×10^{-10}
	IL17	1.30	0.1	0.8	0.0062

Figure S16. Comparison of unstimulated PBMC subset frequencies in BCG recipients and TB-Resisters; related to Fig 6. Data were derived from 13 TB-Resisters and 14 BCG recipients. Forest plot displays ln-transformed odds ratios (lnOR) and 95% confidence intervals (CI). The dotted line shows no average effect (lnOR = 0, corresponding to OR = 1). Features on the right side of this line are more highly expressed among TB-Resisters (OR > 1), and features on the left side are more highly expressed among BCG recipients (OR < 1). The table shows the absolute OR and the means of each parameter in TB-resisters and LTBI-participants.

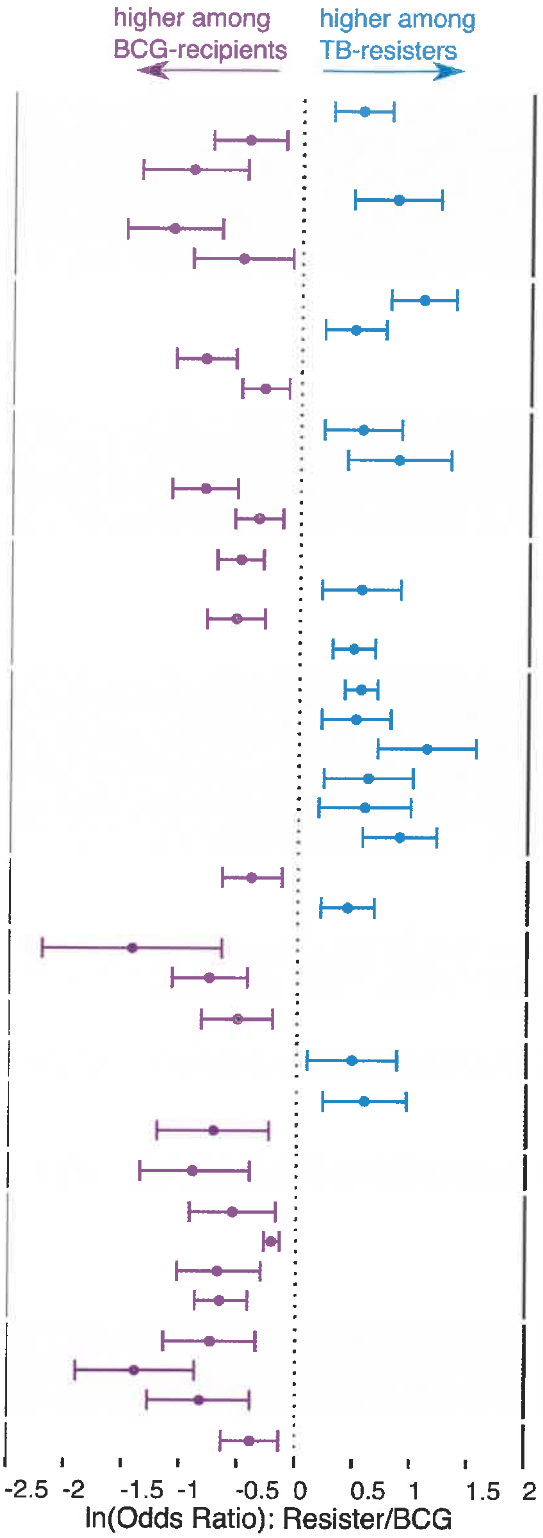

			Mtb-stimulated %		
		OR	BCG	Resister	FDR
cDC1	CD83	1.69	42.1	56.3	0.00042
	IL10	0.63	2.6	0.6	0.0089
	IL1β	0.39	64.9	40.0	0.00042
	IL27	2.29	50.6	71.3	0.00011
	IL8	0.33	74.7	48.8	1.7×10^{-6}
	PDL1	0.60	96.6	92.9	0.048
cDC2	IL27	2.90	53.0	77.7	9.6×10^{-12}
	PDL1	1.60	96.5	98.4	0.002
	CD80	0.44	5.6	1.1	2.8×10^{-8}
	GMCSF	0.73	1.6	0.7	0.0076
Mono	IL27	1.72	2.9	6.5	0.0053
	PDL1	2.36	93.7	99.3	0.0011
	CD80	0.44	5.2	1.1	3×10^{-7}
	GMCSF	0.70	1.2	0.2	0.0025
pDC	IL10	0.60	1.8	0.1	5.2×10^{-6}
	IL27	1.72	22.0	33.7	0.0066
	IL8	0.58	16.9	9.7	0.00014
	TNFα	1.61	0.6	2.1	3.1×10^{-6}
iNKT CD4+	IL10	1.72	0.2	1.8	9.6×10^{-12}
	GMCSF	1.65	0.6	2.6	0.0042
	CD25	3.06	8.8	26.2	3.1×10^{-6}
	CD69	1.84	42.5	57.9	0.0071
	IFNγ	1.79	0.7	3.7	0.013
	Ki67	2.43	3.9	11.6	1.1×10^{-6}
iNKT CD8+	CD107a	0.67	3.7	1.9	0.0081
	IL17	1.55	0.5	1.9	0.0013
MAIT MR1-	GranzB	0.24	48.1	10.1	0.0014
	PD1	0.47	28.7	14.6	3.3×10^{-5}
MAIT MR1+	GranzB	0.60	3.1	1.0	0.0041
NK	IFNγ	1.62	2.3	5.1	0.037
NKT CD4+	CD25	1.81	12.6	22.5	0.0049
	GranzB	0.49	44.6	27.4	0.0089
NKT CD8+	GranzB	0.41	80.2	61.6	0.0014
Tconv CD4+	CD69	0.58	53.3	39.5	0.011
	CD107a	0.81	1.0	0.5	9.5×10^{-9}
	GranzB	0.51	4.9	1.8	0.0013
	PD1	0.52	23.8	13.3	3.6×10^{-7}
Tconv CD8+	CD69	0.48	44.6	27.0	0.0013
	GranzB	0.25	43.2	13.5	3.1×10^{-6}
	PD1	0.44	27.0	11.7	0.0013
γδT	CD69	0.68	59.7	49.9	0.0065

Figure S17. Comparison of *Mtb*-stimulated PBMC subset frequencies in BCG recipients and TB-Resisters; related to Fig 6. Data were derived from 13 TB-Resisters and 14 BCG recipients. Forest plot displays ln-transformed odds ratios (lnOR) and 95% confidence intervals (CI). The dotted line shows no average effect (lnOR = 0, corresponding to OR = 1). Features on the right side of this line are more highly expressed among TB-Resisters (OR > 1), and features on the left side are more highly expressed among BCG recipients (OR < 1). The table shows the absolute OR and the means of each parameter in TB-resisters and LTBI-participants.

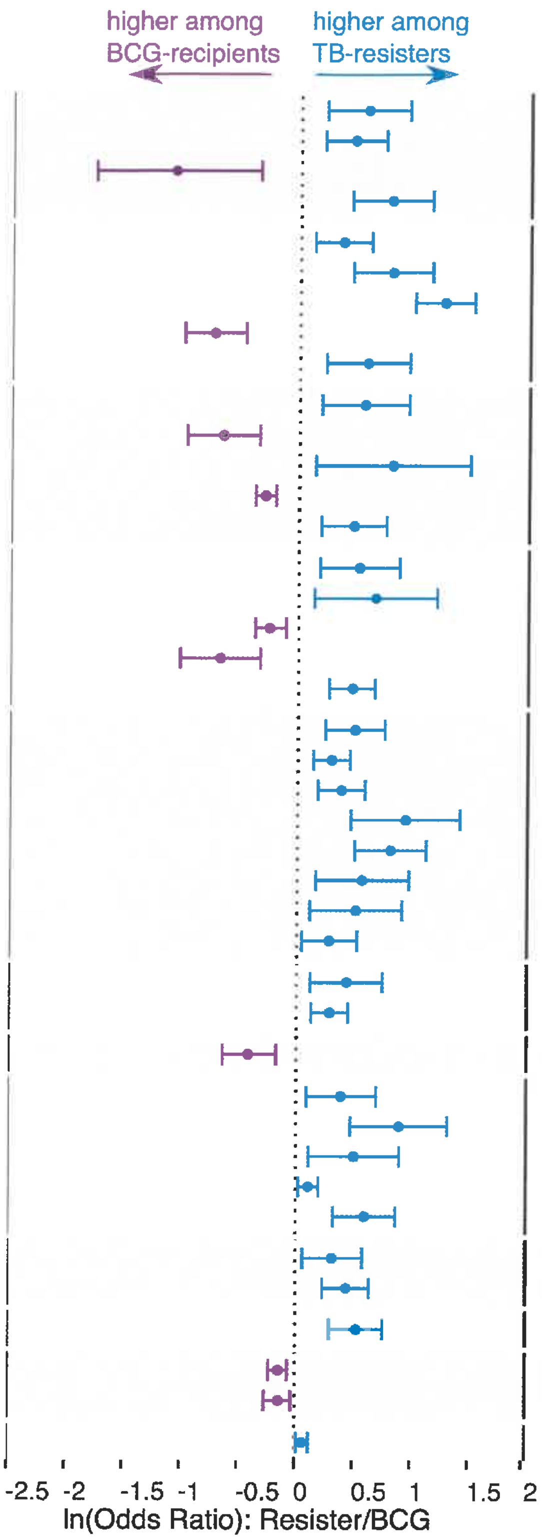

Subset	Feature	OR	Δ: stim % - unstim % BCG	Δ: stim % - unstim % Resister	FDR
cDC1	CD40	1.80	12.3	25.7	0.0055
	CD83	1.61	10.1	11.9	0.0023
	IL1β	0.34	41.7	38.1	0.013
	IL27	2.22	25.7	42.1	9.8×10^{-5}
cDC2	CD83	1.46	-2.4	12.2	0.0099
	IL1β	2.24	39.5	68.3	6.2×10^{-5}
	IL27	3.52	23.1	59.9	6.4×10^{-20}
	CD80	0.48	2.6	-0.4	4.5×10^{-6}
	PDL1	1.81	10.8	38.2	0.005
Mono	IL27	1.77	1.6	6.0	0.011
	CD80	0.52	-4.2	-4.9	0.00033
	PDL1	2.26	17.8	58.5	0.046
	GMCSF	0.75	0.7	-0.2	1×10^{-9}
	IL10	1.62	1.0	3.3	0.0036
pDC	IL27	1.70	11.3	23.8	0.0099
	PDL1	1.96	35.3	64.5	0.038
	IL10	0.78	-0.2	-0.2	0.0023
	IL8	0.51	10.9	8.2	0.0011
	TNFα	1.61	0.4	1.2	4.2×10^{-5}
iNKT CD4+	GMCSF	1.65	0.1	2.0	0.00088
	IL10	1.35	0.1	1.0	0.0016
	CD107a	1.47	1.1	4.4	0.0013
	CD25	2.56	2.9	14.6	0.00075
	CD69	2.25	-5.3	17.4	7.2×10^{-6}
	IFNγ	1.76	0.3	3.5	0.022
	Ki67	1.67	-0.5	-5.0	0.034
	PD1	1.33	-3.8	2.3	0.05
iNKT CD8+	CD69	1.55	-1.2	15.0	0.022
	PD1	1.34	-4.5	3.1	0.0023
MAIT MR1+	GranzB	0.66	1.6	0.3	0.0031
NK	CD25	1.48	6.4	10.6	0.032
	CD69	2.45	9.8	42.1	0.00024
	IFNγ	1.66	1.9	5.0	0.034
	Ki67	1.12	-0.9	-0.2	0.034
	GranzB	1.82	-3.5	9.4	0.00013
NKT CD4+	CD25	1.38	5.5	9.6	0.038
	CD69	1.56	4.0	15.9	0.00016
NKT CD8+	CD69	1.70	2.1	13.7	9×10^{-5}
Tconv CD4+	CD107a	0.87	0.2	0.2	0.0033
	GranzB	0.87	0.3	0.2	0.042
γδT	Ki67	1.07	-0.1	0.1	0.029

Figure S18. Comparison of *Mtb*-memory PBMC subset frequencies in BCG recipients and TB-Resisters; related to Fig 6. Data were derived from 13 TB-Resisters and 14 BCG recipients. Forest plot displays ln-transformed odds ratios (lnOR) and 95% confidence intervals (CI). The dotted line shows no average effect (lnOR = 0, corresponding to OR = 1). Features on the right side of this line are more highly expressed among TB-Resisters (OR > 1), and features on the left side are more highly expressed among BCG recipients (OR < 1). The table shows the absolute OR and the means of each parameter in TB-resisters and LTBI-participants.

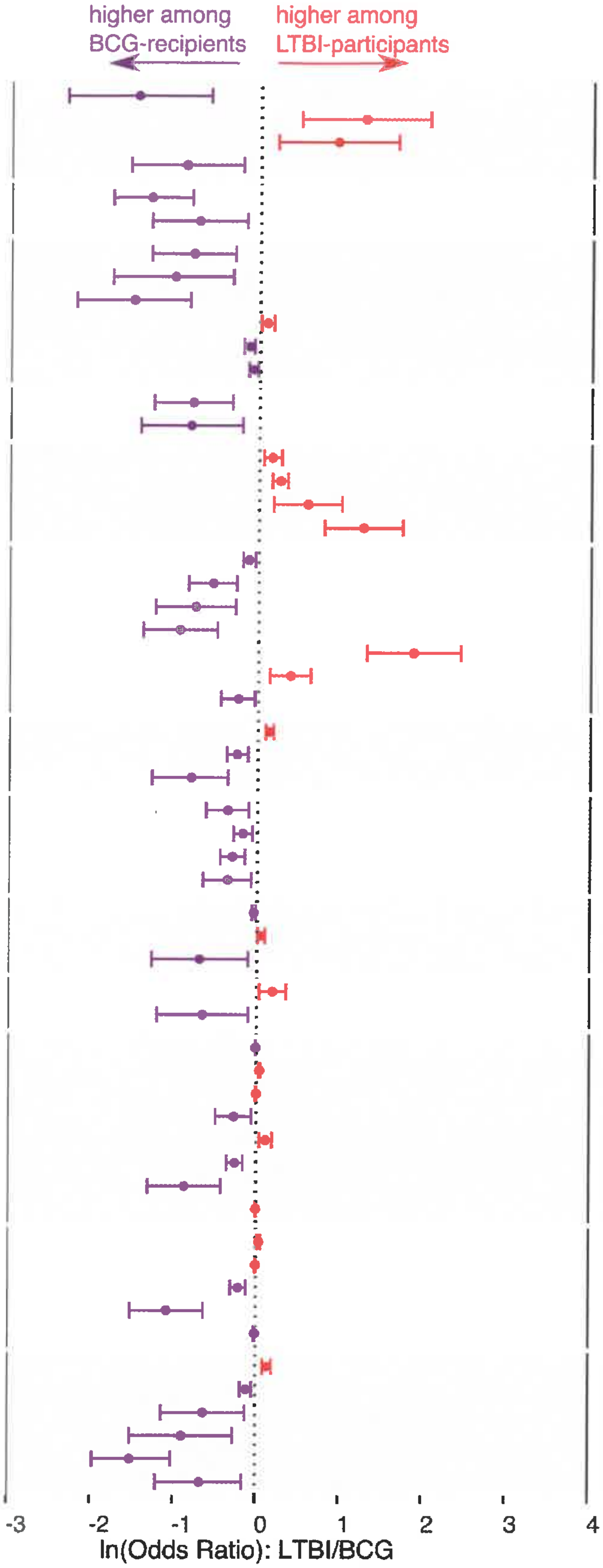

Cell type	Feature	OR	unstimulated % BCG	unstimulated % LTBI	FDR
cDC1	CD80	0.23	36.5	6.3	0.004
	CD83	3.57	17.6	44.4	0.0058
	IL27	2.56	14.0	29.2	0.029
	IL8	0.41	31.1	10.0	0.029
cDC2	IL8	0.27	33.8	13.5	1.2×10^{-6}
	IL1β	0.48	10.8	3.0	0.029
Mono	CD83	0.45	6.5	1.3	0.0065
	IL8	0.36	67.0	42.8	0.015
	IL1β	0.22	27.5	8.1	8.4×10^{-5}
	GMCSF	1.10	0.2	0.4	0.029
	IL10	0.89	0.4	0.0	0.00081
	TNFα	0.93	0.2	0.0	0.029
pDC	IL8	0.45	6.2	1.5	0.004
	PDL1	0.44	21.8	9.1	0.029
iNKT CD4+	GMCSF	1.18	0.1	0.6	0.01
	IL10	1.30	0.1	0.7	9×10^{-7}
	CD25	1.81	5.3	11.6	0.016
	Ki67	3.55	3.0	16.6	2×10^{-6}
iNKT CD8+	TNFα	0.89	0.4	0.1	0.01
	CD25	0.58	3.1	0.6	0.0014
	CD107a	0.47	8.0	1.3	0.0099
	CD69	0.39	39.0	18.2	0.00027
	GranzB	6.54	51.0	90.1	6.9×10^{-9}
	IL17	1.48	0.9	2.1	0.006
	IL2	0.79	0.8	0.1	0.046
MAIT MR1-	IL10	1.15	0.1	0.4	1.2×10^{-6}
	CD107a	0.78	0.9	0.2	0.00081
	CD69	0.45	51.5	31.4	0.0026
MAIT MR1+	CD25	0.70	1.7	0.4	0.017
	Ki67	0.84	0.8	0.3	0.011
	CD107a	0.74	1.2	0.3	0.00035
	PD1	0.70	2.7	1.2	0.036
NK	GMCSF	0.96	0.1	0.0	9.2×10^{-6}
	IL10	1.05	0.1	0.2	0.029
	CD69	0.50	54.4	38.6	0.046
NKT CD4+	IL10	1.21	0.2	0.7	0.044
	GranzB	0.52	35.7	23.8	0.046
Tconv CD4+	GMCSF	0.99	0.0	0.0	2.3×10^{-5}
	IL10	1.04	0.0	0.1	2.5×10^{-8}
	TNFα	1.00	0.0	0.0	0.029
	CD25	0.76	13.2	9.9	0.029
	Ki67	1.12	0.6	0.9	0.011
	CD107a	0.77	1.0	0.3	2×10^{-6}
	CD69	0.42	55.2	33.8	0.00076
	IL2	1.00	0.0	0.0	0.0022
Tconv CD8+	IL10	1.04	0.0	0.1	5.9×10^{-5}
	TNFα	1.00	0.0	0.0	0.02
	CD107a	0.81	0.8	0.2	3.2×10^{-5}
	CD69	0.34	50.3	24.9	2.8×10^{-5}
	IL2	0.99	0.0	0.0	0.004
γδT	IL10	1.15	0.1	0.4	1.2×10^{-6}
	TNFα	0.89	0.3	0.0	0.004
	CD25	0.53	6.0	0.8	0.036
	CD107a	0.41	10.4	0.5	0.015
	CD69	0.22	61.5	27.3	1.9×10^{-8}
	IFNγ	0.51	4.5	0.1	0.029

Figure S19. Comparison of unstimulated PBMC subset frequencies in BCG recipients and LTBI-participants; related to Fig 6. Data were derived from 11 LTBI-participants and 14 BCG recipients. Forest plot displays ln-transformed odds ratios (lnOR) and 95% confidence intervals (CI). The dotted line shows no average effect (lnOR = 0, corresponding to OR = 1). Features on the right side of this line are more highly expressed among LTBI-participants (OR > 1), and features on the left side are more highly expressed among BCG recipients (OR < 1). The table shows the absolute OR and the means of each parameter in TB-resisters and LTBI-participants.

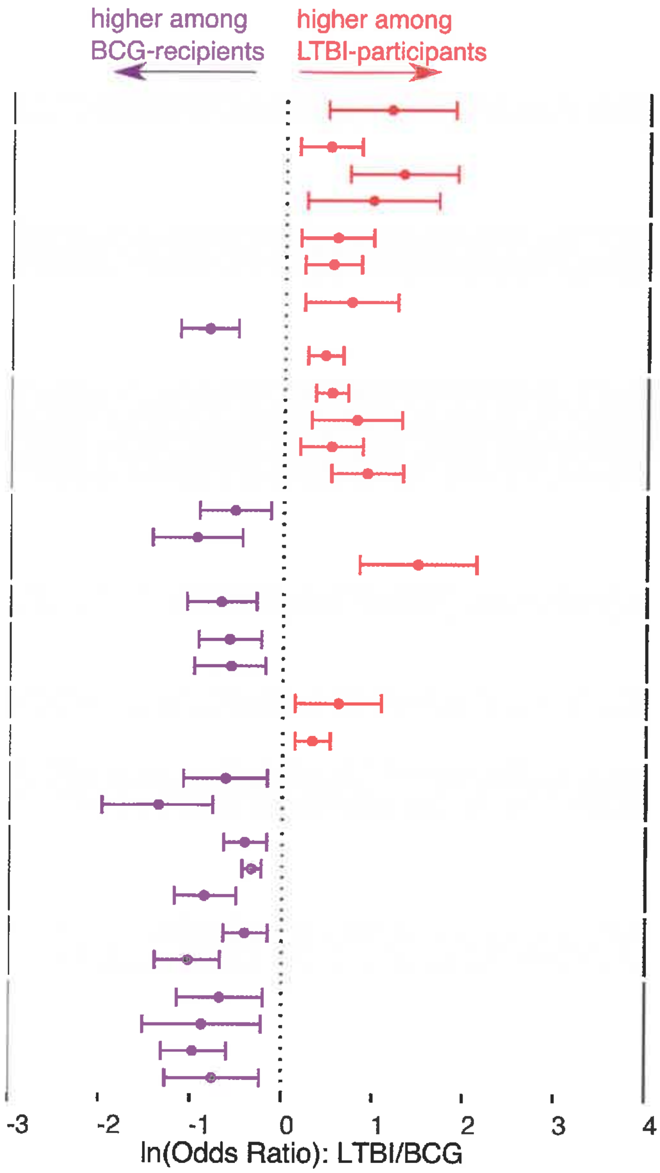

Cell type	Marker	OR	Mtb-stimulated % BCG	Mtb-stimulated % LTBI	FDR
cDC1	CD83	3.21	38.4	56.3	0.0065
cDC2	IL1β	1.64	59.5	71.2	0.02
	IL27	3.68	47.5	77.7	0.00017
	PDL1	2.63	83.5	98.4	0.032
Mono	IL27	1.78	2.8	6.5	0.02
	IL10	1.70	1.1	3.4	0.0057
pDC	IL27	2.09	20.6	33.7	0.02
	IL8	0.44	23.0	9.7	6.7×10^{-6}
	TNFα	1.57	0.5	2.1	6.6×10^{-5}
iNKT CD4+	IL10	1.69	0.2	1.8	2.9×10^{-7}
	CD25	2.23	13.4	26.2	0.0086
	GMCSF	1.69	0.4	2.6	0.013
	Ki67	2.51	3.7	11.6	6.8×10^{-5}
iNKT CD8+	CD25	0.59	7.2	2.8	0.031
	CD107a	0.39	11.4	1.9	0.0015
	GranzB	4.42	51.9	86.0	7.5×10^{-5}
MAIT MR1-	CD69	0.51	53.0	35.9	0.0045
MAIT MR1+	CD25	0.56	7.4	2.7	0.0059
	GranzB	0.57	4.1	1.0	0.019
NK	CD69	1.85	67.0	80.7	0.035
NKT CD4+	IL10	1.39	0.3	1.3	0.0059
NKT CD8+	CD25	0.54	16.0	7.6	0.032
	CD107a	0.26	31.1	6.3	7.7×10^{-5}
Tconv CD4+	CD25	0.67	21.5	15.1	0.0059
	CD107a	0.72	1.4	0.5	4.4×10^{-8}
	CD69	0.43	60.6	39.5	2.3×10^{-5}
Tconv CD8+	CD25	0.67	3.2	1.5	0.0065
	CD69	0.36	52.0	27.0	7.3×10^{-7}
γδT	CD25	0.51	9.4	2.7	0.02
	CD107a	0.42	14.6	1.5	0.032
	CD69	0.38	71.5	49.9	2.6×10^{-6}
	IFNγ	0.47	8.5	1.6	0.02

Figure S20. Comparison of *Mtb*-stimulated PBMC subset frequencies in BCG recipients and LTBI-participants; related to Fig 6. Data were derived from 11 LTBI-participants and 14 BCG recipients. Forest plot displays ln-transformed odds ratios (lnOR) and 95% confidence intervals (CI). The dotted line shows no average effect (lnOR = 0, corresponding to OR = 1). Features on the right side of this line are more highly expressed among LTBI-participants (OR > 1), and features on the left side are more highly expressed among BCG recipients (OR < 1). The table shows the absolute OR and the means of each parameter in TB-resisters and LTBI-participants.

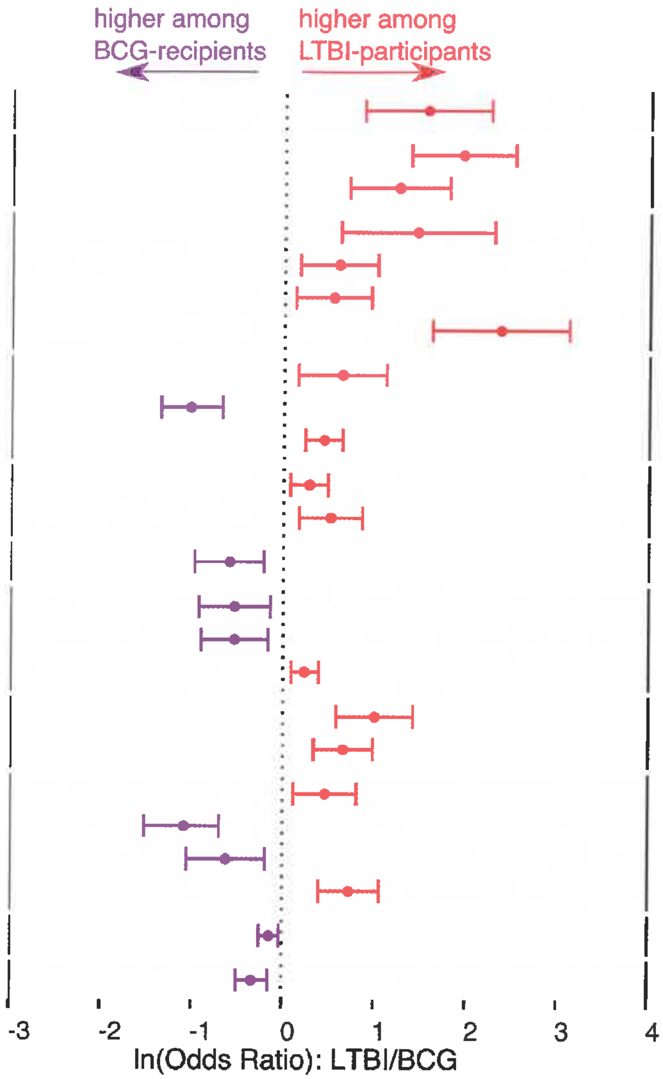

			Δ: stim % - unstim %		
		OR	BCG	LTBI	FDR
cDC1	PDL1	4.77	9.9	36.2	0.00015
cDC2	PDL1	7.03	17.7	38.2	1.6×10^{-9}
	IL27	3.51	35.8	59.9	9.9×10^{-5}
Mono	PDL1	4.29	30.4	58.5	0.0058
	IL27	1.83	2.1	6.0	0.031
	IL10	1.73	0.8	3.3	0.042
	IL8	10.72	16.7	52.2	3×10^{-8}
pDC	IL27	1.91	11.2	23.8	0.039
	IL8	0.36	16.8	8.2	2.5×10^{-7}
	TNFα	1.56	0.2	1.2	0.00018
iNKT CD4+	IL10	1.33	0.1	1.0	0.034
	CD69	1.68	3.0	17.4	0.02
iNKT CD8+	CD107a	0.56	3.4	0.6	0.018
MAIT MR1+	CD25	0.59	5.7	2.3	0.039
	GranzB	0.59	3.2	0.3	0.029
	PD1	1.27	0.4	0.6	0.01
NK	CD69	2.74	12.5	42.1	4.6×10^{-5}
	CD25	1.94	3.5	10.6	0.00055
NKT CD8+	CD69	1.60	3.6	13.7	0.039
	CD107a	0.34	12.4	-1.9	3.3×10^{-6}
	CD25	0.54	8.5	2.1	0.029
	GranzB	2.08	-9.4	8.1	0.00017
Tconv CD4+	CD107a	0.87	0.4	0.2	0.044
Tconv CD8+	CD25	0.72	1.8	0.5	0.0032

Figure S21. Comparison of *Mtb*-memory PBMC subset frequencies in BCG recipients and LTBI-participants; related to Fig 6. Data were derived from 11 LTBI-participants and 14 BCG recipients. Forest plot displays ln-transformed odds ratios (lnOR) and 95% confidence intervals (CI). The dotted line shows no average effect (lnOR = 0, corresponding to OR = 1). Features on the right side of this line are more highly expressed among LTBI-participants (OR > 1), and features on the left side are more highly expressed among BCG recipients (OR < 1). The table shows the absolute OR and the means of each parameter in TB-resisters and LTBI-participants.

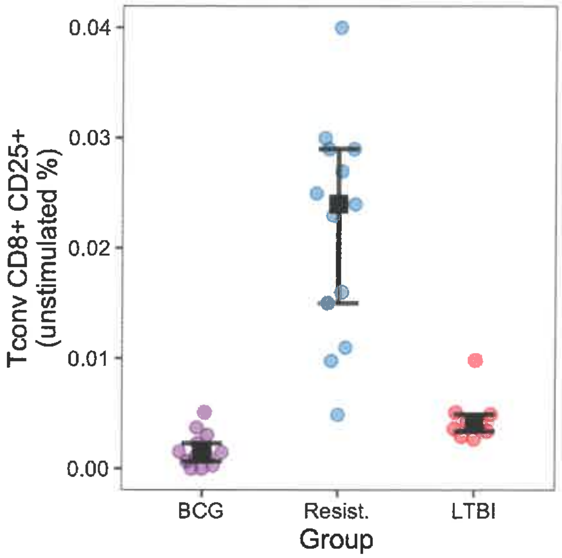

Figure S22. CD8+GMM+GranzB+ T cells in BCG recipients compared with TB-resisters and LTBI-participants; related to Fig 5. The graph shows results from 14 BCG-recipients, 13 TB-resisters and 9 LTBI-participants. Medians, upper and lower quartiles are indicated on the graph.
